# Spectroscopic and computational studies of nitrile hydratase: insights into geometric and electronic structure and the mechanism of amide synthesis†
†Electronic supplementary information (ESI) available: DFT geometry optimization procedure, simulation of EPR spectra, *g* tensor coordinate axes, MO isosurface contours for NHaseBA and NHaseAq, and optimized coordinates for computational models. See DOI: 10.1039/c5sc02012c


**DOI:** 10.1039/c5sc02012c

**Published:** 2015-07-30

**Authors:** Kenneth M. Light, Yasuaki Yamanaka, Masafumi Odaka, Edward I. Solomon

**Affiliations:** a Department of Chemistry , Stanford University , Stanford , CA 94305 , USA . Email: edward.solomon@stanford.edu; b Department of Biotechnology and Life Science , Tokyo University of Agriculture and Technology , 2-24-16 Naka-cho , Koganei , Tokyo , Japan . Email: modaka@cc.tuat.ac.jp

## Abstract

Nitrile hydratases (NHases) are mononuclear nonheme enzymes that catalyze the hydration of nitriles to amides. NHase is unusual in that it utilizes a low-spin (LS) Fe^III^ center and a unique ligand set comprised of two deprotonated backbone amides, cysteine-based sulfenic acid (RSO(H)) and sulfinic acid (RSO_2_^–^), and an unmodified cysteine *trans* to an exogenous ligand site. Electron paramagnetic resonance (EPR), magnetic circular dichroism (MCD) and low-temperature absorption (LT-Abs) spectroscopies are used to determine the geometric and electronic structures of butyrate-bound (NHaseBA) and active (NHaseAq) NHase. These data calibrate DFT models, which are then extended to explore the mechanism of nitrile hydration by NHase. In particular, the nitrile is activated by coordination to the LS Fe^III^ and the sulfenate group is found to be deprotonated and a significantly better nucleophile than water that can attack the coordinated nitrile to form a cyclic species. Attack at the sulfenate S atom of the cyclic species is favorable and leads to a lower kinetic barrier than attack by water on coordinated, uncyclized nitrile, while attack at the C of the cyclic species is unfavorable. The roles of the unique ligand set and low-spin nature of the NHase active site in function are also explored. It is found that the oxidized thiolate ligands are crucial to maintaining the LS state, which is important in the binding and activation of nitrile susbtrates. The dominant role of the backbone amidate ligands appears to be as a chelate in keeping the sulfenate properly oriented for nucleophilic attack on the coordinated substrate.

## Abstract 2

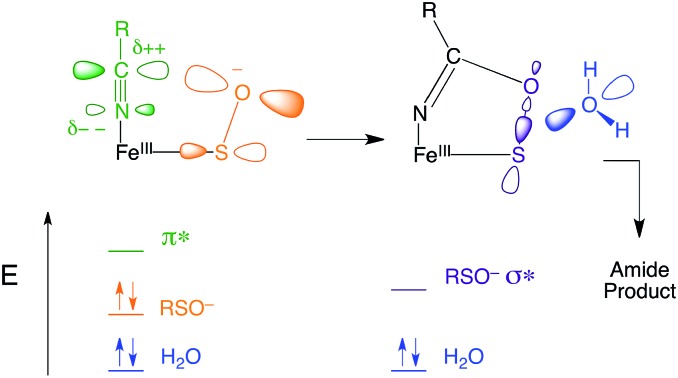
In addition to its activation of coordinated nitriles, nitrile hydratase utilizes a coordinated sulfenate ligand as a well-oriented nucleophile to form a five-membered intermediate which subsequently undergoes attack by H_2_O to ultimately form the amide product.

## Introduction

**Fig. 1:**
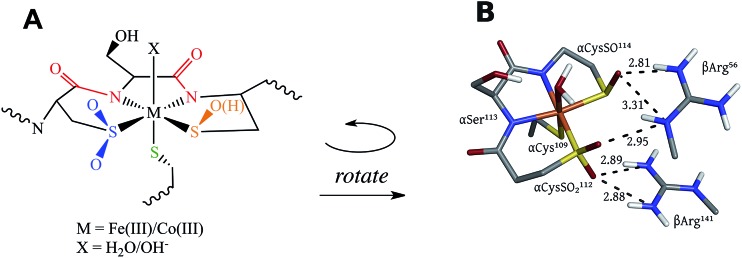
The active site structure of NHase. (A) Diagram of the NHase active site, depicting the thiolate (green), amidate (red), sulfinate (blue), sulfenate/sulfenic acid (orange) and water-derived (denoted X) ligands. (B) The Fe(iii) active site of *Rhodococcus erythropolis* N771 NHase after photolysis (PDB ID ; 2CYZ). Heavy atom distances showing H bonding are marked with dashed lines. All distances are in Å.

Nitriles produced by plants and animals are a source of carbon and nitrogen for some microorganisms. Nitrile hydratases (NHases) are enzymes found in bacteria that catalyze the hydrolysis of nitriles to amides as part of the nitrile degradation pathway.^1^ NHases have been used industrially as catalysts for the production of methacrylonitrile and nicotinamide,^2^ and have also been used in the synthesis of chiral amides^3^ and possess the potential to treat industrial wastewater.^4^ As shown in Fig. 1A, NHases possess an active site that uses either low-spin (LS) Fe^III^ or LS Co^III^ complexed to a very unusual ligand set.^5^,^6^ This set is comprised of two deprotonated backbone amides or amidates, a cysteine thiolate, cysteine-derived post-translationally modified sulfenic/sulfenate (Cys-SO(H)) and sulfinate (Cys-SO_2_^–^) groups, and an exogenous ligand (X). The protonation state of the Cys-SO(H) group in the active form of the enzyme has not been unambiguously determined, with conflicting spectroscopic evidence for the sulfenate and sulfenic acid forms in the literature.^7^,^8^

**Fig. 2:**
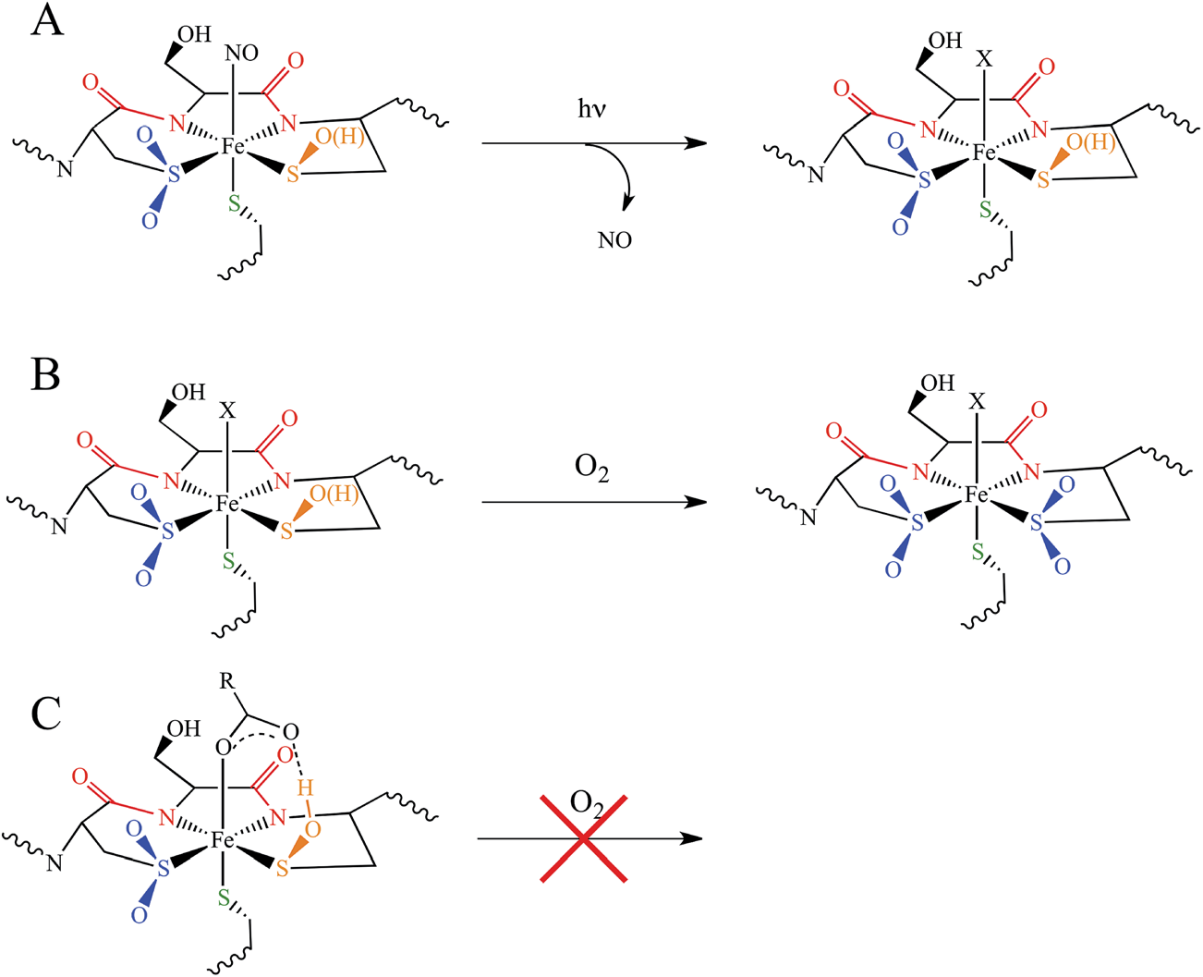
NHase active site forms. (A) Photolytic cleavage of Fe-bound NO to produce the active form. (B) Oxidation of the active form sulfenate/sulfenic acid group in aerobic conditions. (C) Protection of the active site through complexation with butyrate.

NHases are αβ heterodimers with the metal ligand residues residing in the α subunit. The Cys-SO_2_^–^ and Cys-SO(H) residues H-bond with two arginines on the β subunit, as shown in Fig. 1B.^5^,^6^ For the Fe^III^ NHase of *Rhodococcus erythropolis* N771 βArg56 (Fig. 1B) was found to be essential for catalysis. In Co^III^ NHases the sixth ligand (X in Fig. 1A) is derived from water,^6^ whereas Fe^III^ NHase produced in the dark has a NO bound to Fe that is photolytically cleaved to produce the active form containing a water-derived ligand (Fig. 2A).^9^–^11^ If left exposed to air for a sufficient period of time, the Cys-SO(H) ligand is oxidized to Cys-SO_2_^–^ and the enzyme becomes inactive (Fig. 2B).^12^ However, butyric acid may be added to act as a protecting agent, binding to Fe and inhibiting further oxidation of the Cys-SO(H) group (Fig. 2C).^13^–^15^ Butyric acid has also been found to be a competitive inhibitor, which becomes more strongly inhibiting with decreasing pH.^16^ This indicates that it is the protonated form of the acid that stabilizes the enzyme (although from EPR data and DFT calculations (*vide infra*) the proton transfers to the sulfenate group upon butyric acid coordination to NHase Fe^III^).

**Fig. 3:**
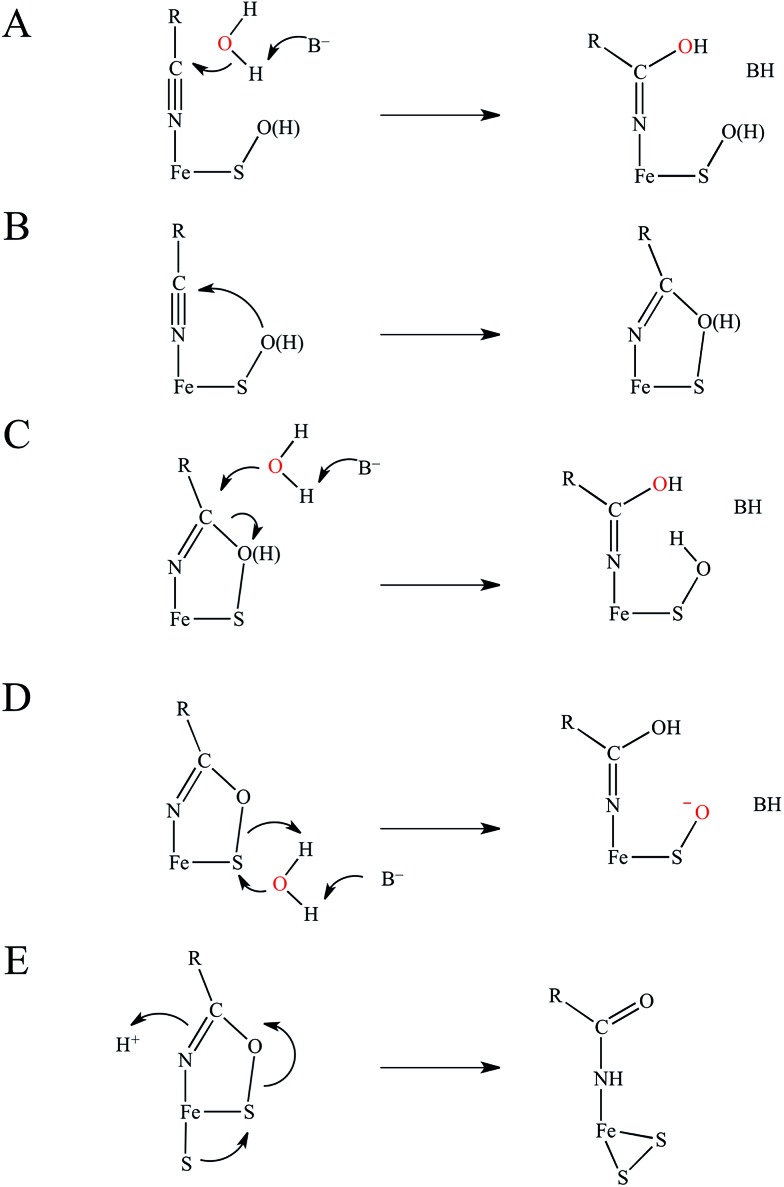
Potential mechanisms for NHase catalysis. (A) Activation of coordinated nitrile for nucleophilic attack by water. (B) Nucleophilic attack by RSO(H) and subsequent activation for attack by water on either (C) the nitrile carbon, or (D) the RSO(H) sulfur. The oxygen atom originating from water is shown in red. (E) Nucleophilic attack on the cyclic intermediate by the axial thiolate.

The catalytic mechanism by which these enzymes operate has not yet been fully elucidated. The fact that the coordinated butyrate acts as a competitive inhibitor suggests that nitrile is activated for nucleophilic attack by coordination to metal, which has provided support for the mechanism of a water attacking this coordinated nitrile with a base accepting a proton as shown in Fig. 3A.^17^–^20^ Recently, a crystallographic study by Holz and coworkers^21^ showing that alkyl boronic acids, also competitive inhibitors, bind to the active site metal of NHase and are nucleophilically attacked by the sulfenate oxygen. This led to the proposal that the RSO(H) itself is the nucleophile that attacks the coordinated nitrile C as shown in Fig. 3B, activating it for nucleophilic attack by water on either the C or S atoms (Fig. 3C and D, respectively).^21^ A related mechanism has been proposed^22^ that involves the axial thiolate acting as the initial nucleophile and the subsequent formation of a disulfide bond, as shown in Fig. 3E. The protonation states of the water-derived and Cys-SO(H) ligands in the active form of the enzyme are not well defined, and their determination is important in understanding the mechanism of nitrile hydrolysis.

It is also important to note that a second-sphere mechanism involving nucleophilic attack on an uncoordinated nitrile in the active site pocket by a coordinated hydroxide has been proposed and found to have a theoretically similar barrier to coordinated nitrile activation *via* DFT calculations.^20^,^23^ However, such a mechanism is not in agreement with the crystallographic results involving boronic acids described above.^21^

**Fig. 4:**
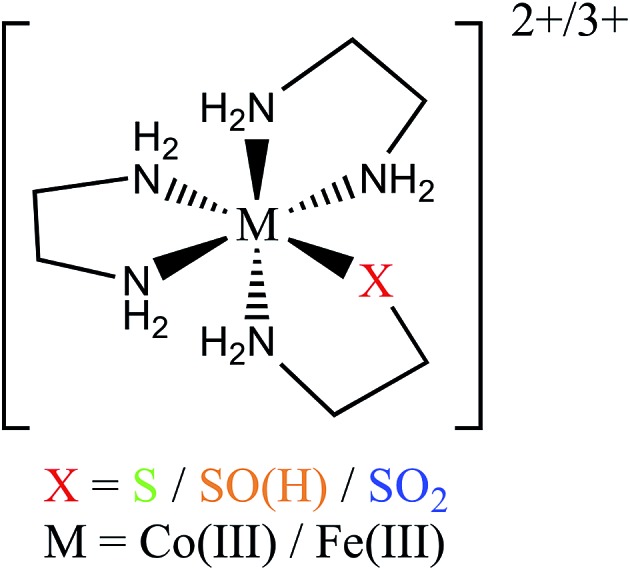
General structure of the [(en)_2_M(XCH_2_CH_2_NH_2_)–*N*,*S*]^2+/3+^ complexes.

In this study we use electronic paramagnetic resonance (EPR), absorption, and magnetic circular dichroism (MCD) spectroscopies to determine the geometric and electronic structures of the paramagnetic LS Fe^III^ NHase from *Rhodococcus erythropolis* N771 in its butyrate-bound (NHaseBA) and active (NHaseAq) forms. EPR spectra of the oxidized, inactive form of NHase (NHaseOX) further allow us to characterize the protonated and deprotonated forms of NHaseAq. Due to the relatively complex nature of the NHase ligand set with regard to possible ligand-to-metal charge transfer (LMCT) transitions, band assignment is assisted through a density functional theory (DFT) computational investigation of a series of LS Co^III^ complexes [(en)_2_Co(XCH_2_CH_2_NH_2_)–*N*,*S*]^2+/3+^ (Fig. 4) and their hypothetical LS Fe^III^ counterparts, where X is a thiolate, sulfenate, sulfenic acid, or sulfinate group.^24^–^26^ These results provide insight into the relative energy ordering of the LMCT transitions for the different sulfur ligands. We use the subsequently assigned experimental data to calibrate DFT models of the butyrate bound and active forms of NHase and to extend these computational models to examine potential mechanisms for nitrile hydrolysis. These results provide insight into the electronic structure of the unusual active site of NHases and the mechanistic strategy developed by this class of enzymes.

## Results and discussion

### EPR spectroscopy of NHaseBA, NHaseAq, and NHaseOx

**Fig. 5:**
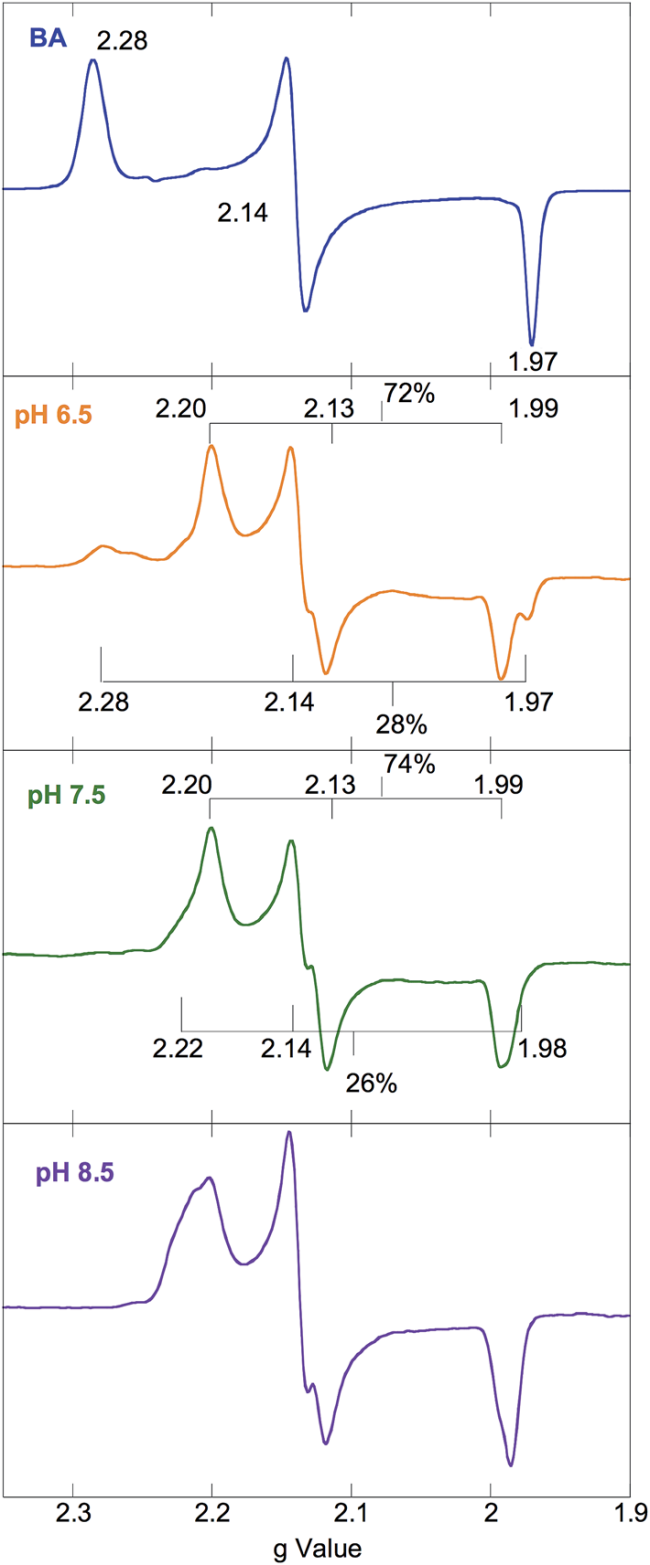
77 K X band EPR spectra of NHaseBA (blue) and NHaseAq at pH 6.5 (orange), 7.5 (green) and 8.5 (purple).

EPR powder pattern spectra of LS Fe^III^ systems generally exhibit 3 features around *g*_eff_ ≈ 2.0.^27^ The *g* values are sensitive to the ligand environment, and can allow the determination of the number of species in a given sample, as well as their relative abundance and a quantitative analysis of the ground state. The EPR spectra of NHaseBA at pH 7.5 and NHaseAq at pH 6.5, 7.5, and 8.5 are shown in Fig. 5. NHaseBA (Fig. 5, blue) is a clean single species with *g* values of 2.28, 2.14 and 1.97. NHaseAq at pH 7.5, on the other hand, is a mixture of 2 species (Fig. 5, green). At pH 8.5 the minor species component has increased in relative contribution (Fig. 5, purple). Simulations of the spectra (see ESI†) indicate that at pH 7.5 NHaseAq is a 74%/26% ± 5% mixture, with the major species having *g* values of 2.20, 2.13, and 1.99, and the minor species having *g* values of 2.22, 2.14 and 1.98. These are indicated in Fig. 5. On going from pH 7.5 to 8.5 the ratio changes to 52%/48% ± 5%. Buffer exchanging the sample back to pH 7.5 leads to the original EPR spectrum (data not shown). These results indicate that the NHaseAq active site possesses a deprotonatable ligand with a p*K*_a_ ≈ 8.5. On going from pH 7.5 to 6.5 (Fig. 5, orange), the EPR spectrum of NHaseAq shows that the high-pH minor species (above) has disappeared, and has been replaced by a third species with *g* values of 2.28, 2.14 and 1.97. The pH 6.5 sample is comprised of approximately 28% of this low-pH minor form and 72% of the major form, indicating a second deprotonatable ligand with a p*K*_a_ ≈ 6.1. This value is identical to the p*K*_a_ determined for the sulfenate of the alkyl hydroperoxide reductase AhpC.^28^ Again, increasing the pH back to 7.5 restores the pH 7.5 spectrum, showing that the process is reversible (data now shown).

**Fig. 6:**
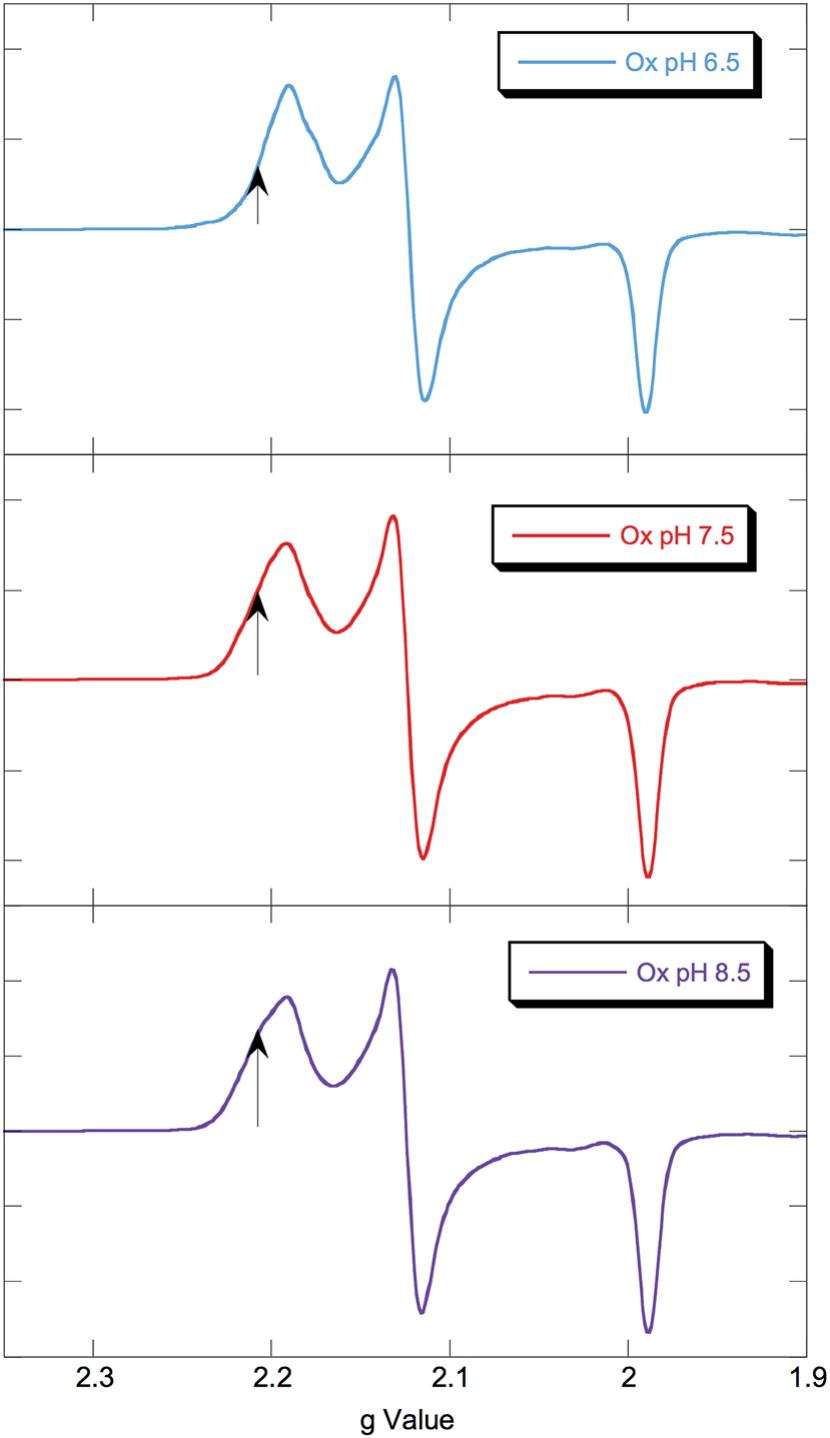
77 K X band EPR spectra of NHaseOx at pH 6.5 (cyan), 7.5 (red), and 8.5 (purple). Signals from minor species at low field marked with arrows.

EPR spectra of NHaseOx at pH 6.5, 7.5 and 8.5 are shown in Fig. 6. Spectra taken at 7.5 (red) and 8.5 (purple) indicate there is an acid–base equilibrium present for NHaseOx similar to that of NHaseAq with a p*K*_a_ > 7 (a shoulder to the low field side of *g* = 2.2 (arrow) increases in intensity on going from pH 7.5 to 8.5). However, on going from pH 7.5 to 6.5 (cyan), no third species is observed. This implies that the moiety in NHaseAq with a p*K*_a_ of 6.1 is the Cys-SO(H) group, and that at the active form present under functional conditions possesses a deprotonated sulfenate ligand. As sulfinic acids generally have p*K*_a_ values of approximately 2,^29^ this means that the moiety in NHaseAq with a p*K*_a_ of 8.5 is the water-derived ligand, and that under functional conditions NHaseAq also possess a coordinated water ligand (X in Fig. 1A).

### NIR MCD spectra of NHaseBA and NHaseAq: low energy d–d transitions

**Fig. 7:**
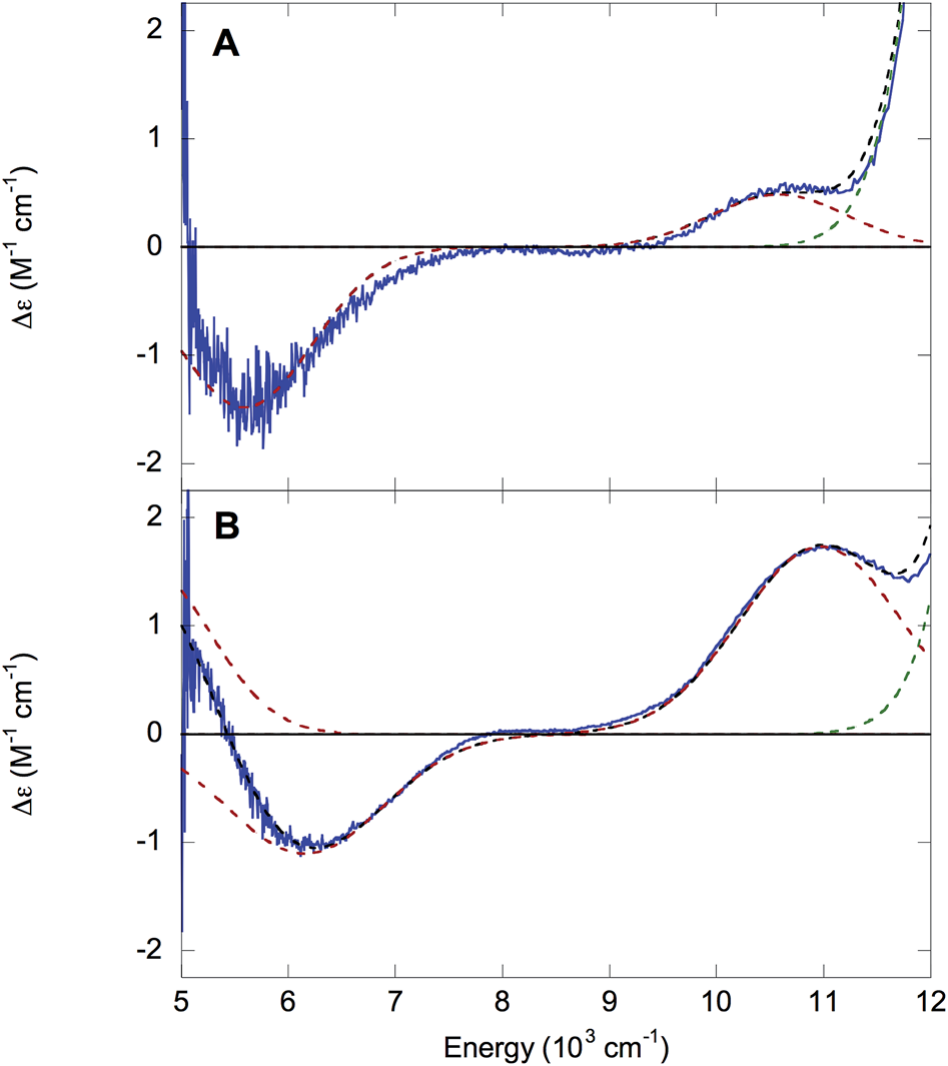
NIR MCD of NHase. 1.5 K, 7 T NIR MCD spectra of NHaseBA (A) and NHaseAq (B) at pD 7.5. Experimental data are in blue, the fit is in black dashes, red dashed curves denote d–d transitions and the green dashed curve denotes the thiolate-to-dπ LMCT.

Octahedral LS Fe^III^ species possess a (t_2g_)^5^ ground configuration giving a threefold orbitally degenerate ^2^T_2g_ ground state, which in the rhombic ligand environment of a protein site splits in energy and leads to two t_2g_ → t_2g_ (dπ → dπ) transitions at ≤5000 cm^–1^. The orbitally degenerate doublet excited states also split to produce a manifold of t_2g_ → e_g_ (dπ → dσ) transitions from ≈15 000 to 30 000 cm^–1^, although the higher-energy ligand field (LF) transitions are frequently obscured by the intense LMCT transitions and may be difficult to detect.^27^ The 1.5 K, 7 T NIR MCD spectra of NHaseBA and NHaseAq at pD 7.5 are shown in Fig. 7A and B, respectively. The spectrum of NHaseBA has a band at ≈5600 cm^–1^ corresponding to the highest energy dπ–dπ transition, as well as a band at ≈10 600 cm^–1^, which corresponds to the lowest energy dπ–dσ transition.^30^ The shoulder of the ≈13 600 cm^–1^ Cys-Sπ → dπ LMCT is also visible as a tail in the higher energy region of this spectrum (*vide infra*). The spectrum of NHaseAq shows two bands at low energy, one with a *ν̃*_max_ < 5000 cm^–1^ (Gaussian fit at ≈4700 cm^–1^) and another with *ν̃*_max_ ≈ 6300 cm^–1^. Thus the dπ–dπ transitions of NHaseAq are raised in energy relative to those of NHaseBA. The lowest energy dπ → dσ transition of NHaseAq is also higher than that of NHaseBA at ≈11 000 cm^–1^.

### Assignment of t_2g_ orbital splittings

As the ground states of six-coordinate LS Fe^III^ active sites are derived from the ^2^T_2g_ states of *O*_h_, in addition to the low symmetry splitting of the dπ orbitals considered above they have in-state orbital angular momentum and thus undergo in-state spin–orbit coupling (SOC). This in-state coupling has a significant effect on the experimental EPR *g* values of a complex. A system of equations developed by Taylor includes this SOC and allows for the determination of the energy splitting of the t_2g_ orbitals as well as the order and sign of the experimental *g* values.^31^ The t_2g_ orbital energies of a given site are defined by the tetragonal splitting, *Δ*, of the d_*xy*_ orbital from the {d_*xz*_, d_*yz*_} pair, and the rhombic splitting, *V*, of the d_*xz*_ and d_*yz*_ orbitals. The quantity |*V*/2*Δ*| is a measure of the rhombicity of the LS Fe^III^ site, with a value of zero indicating a purely axial system and a value of 1/3 being the rhombic limit in which the t_2g_ orbital splittings are equal. The assigned *g* values, energy splitting parameters, and predicted transitions for NHaseBA and NHaseAq are given in Table 1. The values of *a*, *b*, and *c* are the coefficients of the d_*yz*_, d_*xz*_, and d_*xy*_ orbitals, respectively, in the half-occupied dπ ground state. The predicted dπ–dπ transition energies of NHaseBA (≈2800 cm^–1^ and 5300 cm^–1^) and NHaseAq (≈3900 cm^–1^ and 6200 cm^–1^) compare well with the experimental values (≈5600 cm^–1^ for NHaseBA, the lowest energy dπ → dπ transition being below the detection limit of the instrumentation, and ≈4700 cm^–1^ and 6200 cm^–1^ for NHaseAq), indicating that the Taylor method provides an accurate description of the t_2g_ dπ orbital energies. The negative values of *Δ* for NHaseBA and NHaseAq indicate that d_*xy*_ is the half-occupied orbital for both of these forms of the enzyme. This is reflected in the coefficients where *c* is dominant. The |*V*/2*Δ*| value for NHaseBA indicates that this form has a strongly rhombic Fe^III^ site, whereas the value of NHaseAq indicates that its site is closer to axial.

**Table 1:** Predicted *g* values, dπ coefficients, dπ splitting parameters and dπ–dπ transitions for NHaseBA and NHaseAq from the Taylor method. Splitting parameter and transition values are in cm^–1^

Form	*g*_*x*_	*g*_*y*_	*g*_*z*_	*a*	*b*	*c*	*Δ*	|*V*|	|*V*/2*Δ*|	Trans. 1	Trans. 2
BA	–2.14	2.29	–1.97	0.07	0.04	1.00	–4030	2560	0.32	2750	5300
Aq	–2.13	2.21	–1.99	0.05	0.03	1.00	–5010	2320	0.23	3850	6170

### LS Co^III^ and Fe^III^ models: bonding interactions and their associated LMCT transitions

**Fig. 8:**
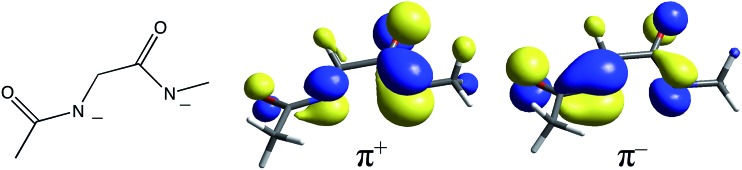
The diamidate moiety in NHase (left) and its in-phase (center) and out-of-phase (right) π MO combinations.

The unusual ligation of NHase allows a number of different possible CT transitions, whose nature and energy order need to be clarified. One may initially anticipate thiolate π → dπ*, amidate π → dπ*, and thiolate/sulfenate/sulfenic/sulfinate σ → dσ* CT transitions as potentially occurring within the accessible spectral range for UV-Vis absorption (≈11 000–33 000 cm^–1^). It has been previously shown through resonance Raman spectroscopy^32^ that the thiolate π → dπ* CT transition for butyrate-free Fe NHases occurs at ≈14 700 cm^–1^ (680 nm) and shifts to ≈14 100 cm^–1^ (710 nm) upon butyrate binding.^16^ The LS Fe^III^ cyanide-bound form of superoxide reductase (SOR) also exhibits a thiolate π → dπ at ∼15 200 cm^–1^.^33^ Resonance Raman spectroscopy identified amidate π → dπ CT transitions in ferric bleomycin (FeBLM) and activated bleomycin (ABLM) at ≈26 400 cm^–1^ and ≈27 300 cm^–1^, respectively.^30^ NHase has two amidates, whose proximity leads to in-phase (+) and out-of-phase (–) combinations of ligand donor orbitals π^+^ and π^–^ as shown in Fig. 8. Therefore, it is reasonable for two amidate π–dπ* CT transitions to contribute to the spectrum of NHase in the ≈26 000 cm^–1^ energy region.

There has been an interesting study of the UV-Vis absorption spectra of a series of LS Co^III^ complexes [(en)_2_Co(XCH_2_CH_2_NH_2_)–*N*,*S*]^2+/3+^, where X is S^–^, *S*-coordinated SO^–^, *S*-coordinated SOH, and *S*-coordinated SO_2_^–^.^24^–^26^ This series provides a complete set of spectroscopic data for the thiolate-based ligands of interest for which the computational extension to the hypothetical LS Fe^III^ forms is straightforward. The observed LMCT's are given in Table 2. For LS Co^III^ (d^6^) these can only be Lσ → dσ* CT transitions as there is no electron hole in the dπ manifold. The thiolate and sulfinate complexes exhibit LMCT transitions at ≈35 000 cm^–1^, with the latter lower in energy by 800 cm^–1^. The sulfenate complex, however, exhibits a lower energy LMCT transition at 27 400 cm^–1^, in addition to a transition at 35 200 cm^–1^. Addition of protons diminishes the intensity of the lower energy transition, while addition of BF_3_ eliminates it entirely.^26^,^34^

**Table 2:** Experimental Co^III^, TD-DFT Co^III^ and TD-DFT Fe^III^ LMCT transitions for the [(en)_2_Co(XCH_2_CH_2_NH_2_)–*N*,*S*]^2+/3+^ complexes. All transition energies are in cm^–1^

Ligand	Expt'l trans. (*ε*)	Co^III^ TD-DFT	Fe^III^ TD-DFT
Thiolate^*a*^	35 500 (13 800)	39 300 (RSσ–dσ)	19 600 (RSπ–dπ),
			40 600 (RSσ–dσ)
Sulfenate^*a*^ ^,^^*b*^	27 400 (6700)	29 800 (RSOσ_IP_–dσ)	32 700 (RSOσ_IP_–dσ)
	35 200 (3700)	34 900 (RSOσ_OOP_–dσ)	38 000 (RSOσ_OOP_–dσ)
Prot. sulfenate^*a*^	≈35 200^*c*^	39 200 (RSOHσ_IP_–dσ)	42 300 (RSOHσ_IP_–dσ)
		42 800 (RSOHσ_OOP_–dσ)	49 500 (RSOHσ_OOP_–dσ)
Sulfinate^*d*^	34 700 (14 200)	36 600 (RSO_2_σ–dσ)	37 800 (RSO_2_σ–dσ)

^*a*^From ref. 26.

^*b*^Due to the instability of the sulfenato complex the uncertainty on the reported *ε* values is high.

^*c*^No *ε* value given.

^*d*^From ref. 25.

**Fig. 9:**
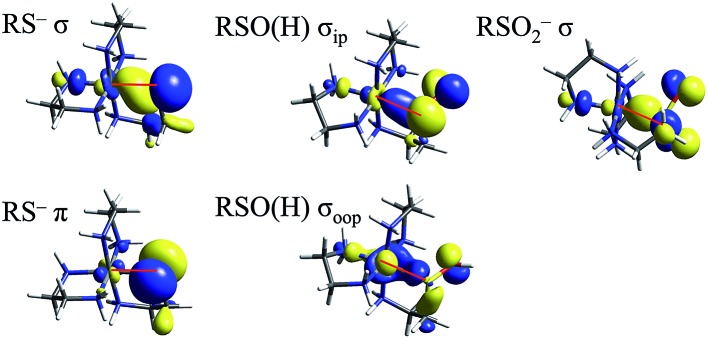
Molecular orbitals of thiolate, sulfenate, and sulfinate ligands involved in model complex LMCT transitions. Red lines mark the Fe–S bond in each picture.

TD-DFT calculations were performed on the LS Co^III^ complexes and their LS Fe^III^ counterparts, with the predicted transition energies listed in Table 2. Contours of the ligand donor molecular orbitals involved in these transitions are shown in Fig. 9. The thiolate ligand has two S 3p orbitals for bonding to metal (the third 3p orbital is involved in bonding to C), one aligned along the metal–S bond axis (RS^–^ σ) positioned for σ bonding and one orthogonal to the bond axis (RS^–^ π) oriented for π bonding. The RSO^–^ and RSOH ligands are S-bonded to the metal and have occupied S–O π* orbitals aligned in the Fe–S–O plane (RSO(H) σ_IP_) and out of the Fe–S–O plane (RSO(H) σ_OOP_) that are oriented for σ overlap with the metal. The sulfinate ligand has an occupied S–(O_2_^–^) π* orbital which is oriented to interact with the metal in a σ fashion (RSO_2_^–^ σ). The calculated Co^III^ transition energies agree qualitatively with the experimental results. The thiolate σ → dσ* transition predicted by TD-DFT is higher in energy than the corresponding sulfinate by 2700 cm^–1^, *vs.* 800 cm^–1^ in the experimental data. The TD-DFT sulfenate σ_IP_ → dσ* transition is lower in energy than the thiolate σ and sulfinate transitions at 29 800 cm^–1^, comparable to the experimental value of 27 400 cm^–1^.

A sulfenate σ_OOP_ → dσ* transition is predicted at 34 900 cm^–1^, but this transition will not be observed in an NHase active site with a deprotonated sulfenate group. The protonated sulfenate complex possesses a calculated RSOH σ_IP_ → dσ* at 39 200 cm^–1^, comparable to the analogous transition in the thiolate complex. A calculated RSOH σ_OOP_ → dσ* transition occurs at a significantly higher energy of 42 800 cm^–1^, which is beyond the protein cutoff for spectroscopy on an enzyme (≈33 000 cm^–1^).

In order to extend the above findings to the LS Fe^III^ site of NHase, Fe^III^ was computationally substituted for Co^III^ in the starting structures of the 4 model complexes above and re-optimized (with an *S* = 1/2 ground state). These hypothetical Fe^III^ complexes have TD-DFT transitions that are comparable to their Co^III^ counterparts, with the Fe^III^ transitions at higher energy due to the lower *Z*_eff_ of Fe^III^ leading to a higher energy d orbital manifold relative to Co^III^. The Fe^III^ thiolate complex also has a dπ* hole due to the LS d^5^ configuration leading to a RS^–^ π → dπ* transition at 19 600 cm^–1^ from TD-DFT, or about 5000 cm^–1^ higher in energy than the corresponding transitions of NHaseBA, NHaseAq and cyanide-bound SOR. Scaling the other Fe^III^ TD-DFT calculated transition energies by a similar amount gives predicted deprotonated sulfenate σ_IP_ → dσ* transition at ≈28 000 cm^–1^ and sulfenate/thiolate/sulfinate σ transitions predicted at 33 000–36 000 cm^–1^ for NHaseBA and NHaseAq. The relative energy order for the LMCT transitions in NHase is therefore predicted to be Cys-S^–^ π → dπ* (experimentally assigned by resonance Raman at ≈15 000 cm^–1^) < amidate π → dπ* ≈ Cys-SO^–^ σ_IP_ → dσ* < Cys-SO_2_^–^ σ_IP_ → dσ* < Cys-S^–^ σ_IP_ → dσ* < Cys-SOH σ_IP_ → dσ*. It should be noted that the Cys-SO^–^ σ_IP_ MO is the highest in energy among the occupied S-based ligands, and has considerable O character (48% O *vs.* 32% S in the Fe^III^ model), indicating that it has the potential to be a good nucleophile (*vide infra*).

### UV-Vis low-temperature absorption and MCD spectroscopy of NHaseBA and NHaseAq

**Fig. 10:**
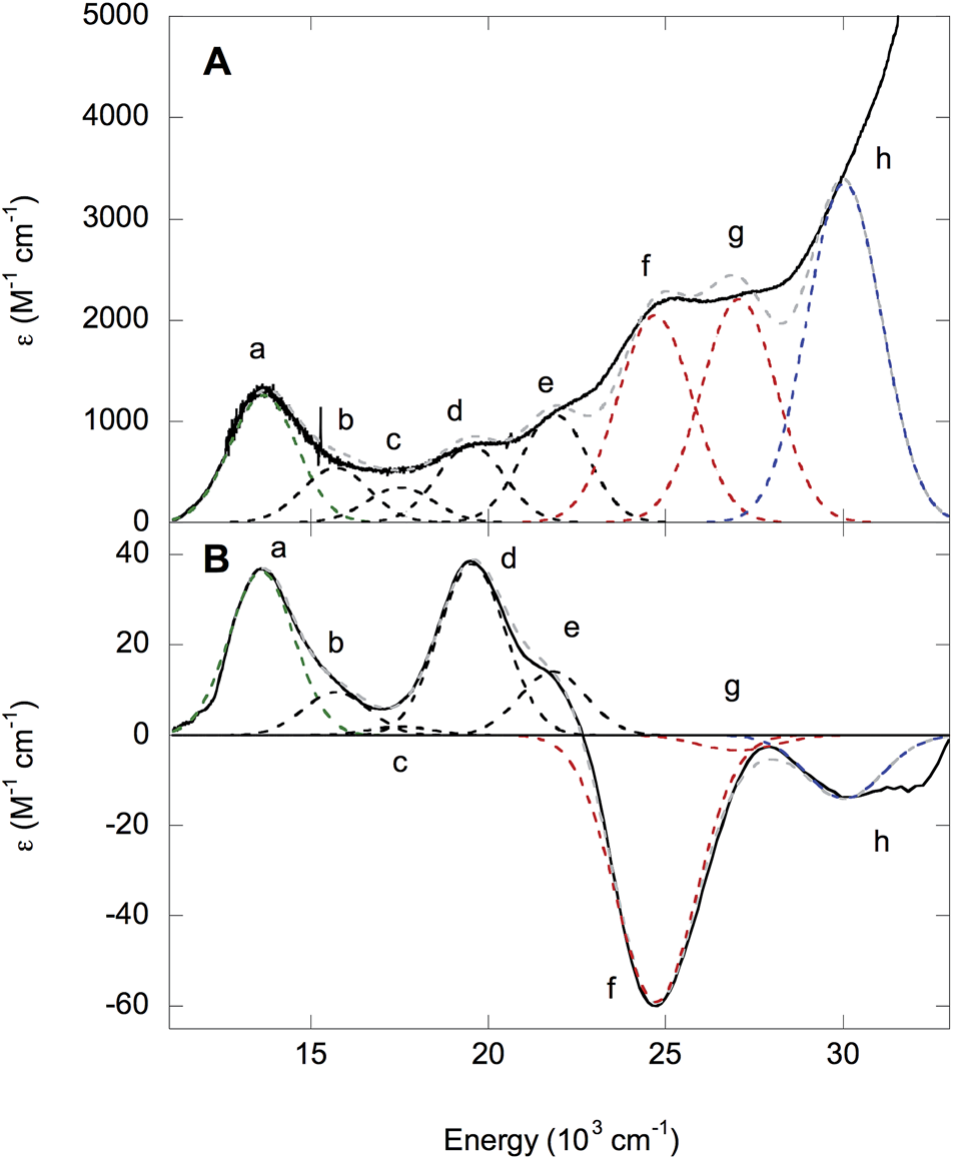
UV-Vis spectra of NHaseBA. (A) Absorption spectrum of NHaseBA at 5 K. (B) 5 K 7 T MCD spectrum of NHase BA. Experimental data are in black, the total fit is in dashed gray, and dashed Gaussian curves denote Cys-S^–^ π → dπ (a, green), dπ → dσ (b–e, black), amidate π → dπ (f–g, red) and Cys-SO_2_^–^ σ → dσ (h, blue) transitions.

The 5 K absorption spectrum and 5 K, 7 T MCD spectrum of NHaseBA are given in Fig. 10A and B, respectively. A list of band energies, *ε* and Δ*ε* values, and *C*/*D* ratios is given in Table 3. Temperature dependence indicates the MCD transitions are all C terms of a paramagnetic complex. The *C*/*D* ratio of a transition is proportional to its Δ*ε*/*ε* value, and is generally higher for d–d than CT transitions.^35^ The Cys-S^–^ π → dπ* LMCT transition occurs at 13 600 cm^–1^ (band a) from resonance Raman spectroscopy.^32^ The transitions at 15 700, 17 500, 19 500 and 21 800 cm^–1^ (bands b, c, d, and e, respectively) are determined by their relative lower *ε* values (and higher Δ*ε* in MCD) as dπ → dσ* ligand field transitions. As NHaseBA has a protonated sulfenate group, the intense in absorption, and therefore CT in nature, transitions at 24 700 and 27 000 cm^–1^ (bands f and g) are ascribed to amidate π → dπ* transitions, while the 30 000 cm^–1^ (band h) transition is assigned to the Cys-SO_2_^–^ σ → dσ* CT transition.

**Table 3:** Energies, absorption *ε* values, MCD Δ*ε* values, and *C*/*D* ratios for the UV-Vis transitions of NHaseBA

Band	Energy (cm^–1^)	*ε* (M^–1^ cm^–1^)	Δ*ε* (M^–1^ cm^–1^)	*C*/*D* × 10^4^
a	13 600	1250	36	306
b	15 700	500	10	213
c	17 500	350	2	61
d	19 500	760	38	532
e	21 800	1060	14	140
f	24 700	2050	–59	306
g	27 000	2200	–3	15
h	30 000	3350	–14	44

The LS nature of the Fe^III^ site implies strong ligand bonds and relatively high covalency, which can lead to significant ligand character in the d-based molecular orbitals and increased absorption intensity for the d–d transitions through LF excited state mixing with CT transitions. The CT transitions of NHaseBA generally have low *C*/*D* ratios, except for the Cys-S^–^ π → dπ* (band a in Fig. 10) and amidate π^+^ → dπ* (band f in Fig. 10) transitions which have significant MCD intensities of opposite sign. This can be explained by the two transitions interacting through SOC to form a pseudo-A term in MCD. The pseudo-A term requires 2 perpendicularly polarized transitions to SOC in a third mutually orthogonal direction.^36^ From the DFT results given in the ESI,† the Cys-S^–^ π → dπ* CT transition is *y*-polarized with the Cys-S^–^ π donor MO having d_*xz*_ character, while the amidate π^+^ → dπ* transition is *x*-polarized with the amidate π^+^ donor MO having d_*yz*_ character. The d_*xz*_ and d_*yz*_ characters of these two donor MOs couple by the *L*_*z*_ component of the angular momentum operator and therefore spin–orbit couple to produce the pseudo-A-term.

**Fig. 11:**
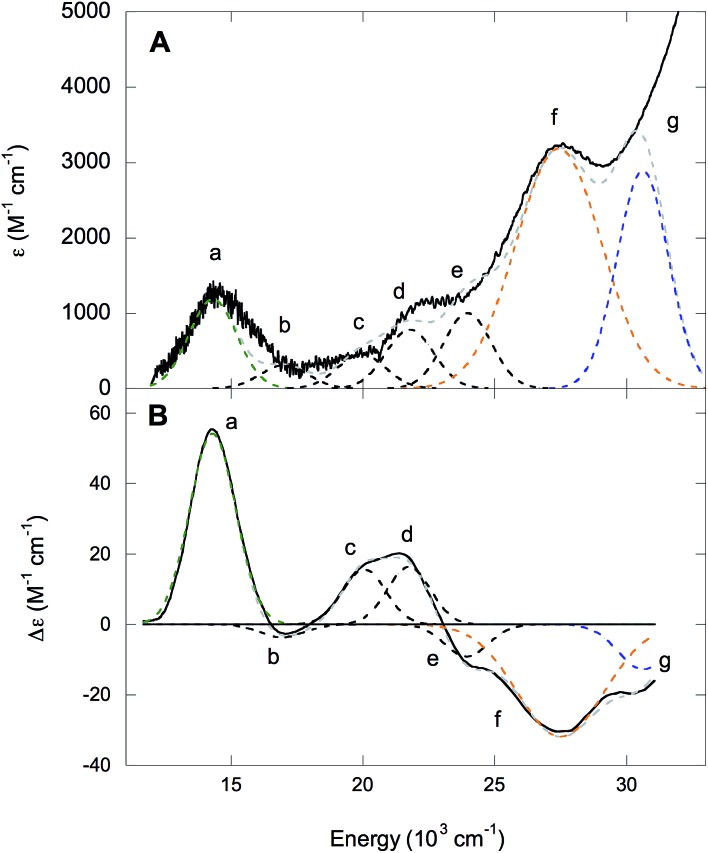
UV-Vis spectra of NHaseAq. (A) Absorption spectrum of NHaseAq at 5 K. (B) 5 K 7 T MCD spectrum of NHaseAq. Experimental data are in black, the total fit is in dashed gray, and dashed Gaussian curves denote Cys-S^–^ π → dπ (a, green), dπ → dσ (b–e, black), amidate π → dπ/Cys-SO^–^ σ → dσ (f, orange) and Cys-SO_2_^–^ σ → dσ (g, blue) transitions.

The absorption and MCD spectra of NHaseAq are given in Fig. 11. A list of band energies, *ε* and Δ*ε* values, and *C*/*D* ratios is given in Table 4. The spectra in Fig. 11 are similar to those in Fig. 10, but with more absorption intensity in the 25 000–30 000 cm^–1^ region associated with the butyrate being replaced by H_2_O and Cys-SOH deprotonated to Cys-SO^–^. NHaseAq possesses a Cys-S^–^ π → dπ* LMCT transition at 14 300 cm^–1^ (band a) which is shifted up from that of NHaseBA by 700 cm^–1^ and is similar to the results previously found in the room temperature absorption spectra for other NHases.^14^ The dπ → dσ* LF transitions of NHaseAq (bands b–e) are also shifted up in energy relative to that of NHaseBA, consistent with the LF effects on the dπ splitting. The amidate π → dπ* CT transitions (band f) are also shifted higher in energy relative to NHaseBA at ≈27 500 cm^–1^. Instead of two features, however, one broad feature is observed with higher intensity than the two amidate π → dπ* CT transitions of NHaseBA (≈2100 M^–1^ cm^–1^ for each NHaseBA transition *vs.* 3200 M^–1^ cm^–1^ for the NHaseAq transition). It is possible that the Cys-SO^–^ σ → dσ* transition (predicted to be in this energy region from the model complex DFT calculations, *vide supra*) overlaps with the two amidate π → dπ* transitions to form this broad, intense feature. The transition in the NHaseAq spectra at ≈30 600 cm^–1^ (band g) is similar in energy and intensity to band h in NHaseBA and is reasonably assigned to the Cys-SO_2_^–^ σ → dσ* CT transition.

**Table 4:** Energies, absorption *ε* values, MCD Δ*ε* values, and *C*/*D* ratios for the UV-Vis transitions of NHaseAq

Band	Energy (cm^–1^)	*ε* (M^–1^ cm^–1^)	Δ*ε* (M^–1^ cm^–1^)	*C*/*D* × 10^4^
a	14 300	1200	54	479
b	16 900	310	–4	137
c	20 100	450	16	378
d	21 700	780	16	218
e	23 900	1000	–9	96
f	27 500	3180	–32	107
b	30 600	2890	–13	48

### DFT and TD-DFT calculations: EPR parameters, dπ orbital splittings and ground state wave functions

**Fig. 12:**
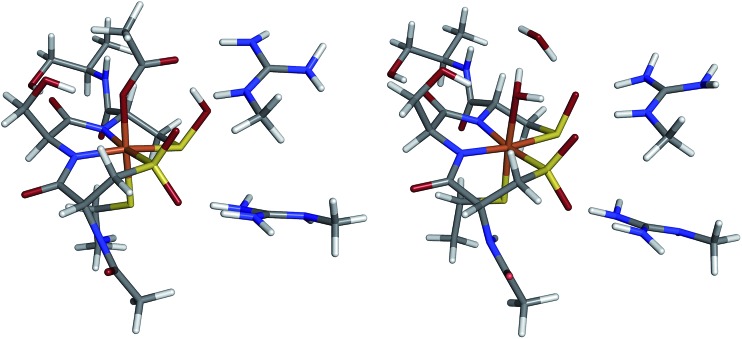
Geometry-optimized structures of NHaseBA (left) and NHaseAq (right).

The DFT optimized structures of NHaseBA and NHaseAq are shown in Fig. 12 (note that both are based on crystal structures). Optimization of an NHaseBA structure with a sulfenate and protonated butyric acid ligand leads to the proton moving to the sulfenate group. NHaseAq contains an exogenous water that is H bonding with both the sulfenate oxygen and the coordinated water ligand. The B3LYP, BP86, and BP86 with 10% Hartree–Fock (HF) functionals were tested by optimizing the NHaseBA and NHaseAq structures in the *S* = 1/2, *S* = 3/2, and *S* = 5/2 states to determine if each functional correctly predicted the ground state spin (*S* = 1/2). The values of Δ*H*(1/2 → 3/2) and Δ*H*(1/2 → 5/2) for the different functionals are given in Table 5. The B3LYP functional predicts the *S* = 1/2 and *S* = 3/2 states are virtually isoenthalpic for both NHaseBA and NHaseAq, rendering this functional unsuitable for modeling the NHase active site. While the BP86 with 10% HF functional predicts an *S* = 1/2 ground state for both NHaseBA and NHaseAq, it predicts Cys-S^–^ π → dπ CT transitions of 16 400 cm^–1^ for both NHaseBA and NHaseAq, whereas the pure BP86 functional predicts the NHaseAq transition to be 500 cm^–1^ higher in energy than the NHaseBA transition (*vide infra*), which is very close to the 700 cm^–1^ difference observed experimentally. The pure GGA BP86 functional was therefore used for all subsequent calculations.

**Table 5:** Δ*H*(1/2 → 3/2) and Δ*H*(1/2 → 5/2) values for the NHaseBA and NHaseAq models geometry-optimized using the BP86, BP86 + 10% HF, and B3LYP functionals. All values are in kcal mol^–1^

Form	Functional	Δ*H*(1/2 → 3/2)	Δ*H*(1/2 → 5/2)
NHaseBA	BP86	8.9	24.7
	BP86 + 10% HF	5.4	16.1
	B3LYP	0.9	4.0
NHaseAq	BP86	11.4	28.5
	BP86 + 10% HF	8.7	20.3
	B3LYP	1.2	6.7

The ORCA program was used to calculate the EPR *g* values for the NHaseBA and NHaseAq models as listed in Table 6. Significant deviations from the largest experimental *g* value for each form of NHase are calculated consistent with past results that DFT tends to underestimate the largest *g* value.^37^ The calculated *g* tensor directions for both the NHaseBA and NHaseAq models indicate that the *x* direction is approximately along the amidate N–Fe–sulfenate S bond axis, the *y* direction is pointing along the thiolate S–Fe–butyrate/water O bond axis and the *z* direction is effectively pointing along the amidate N–Fe–sulfinate S bond axis (see ESI†).

**Table 6:** Experimental and ORCA *g* magnitudes for NHaseBA and NHaseAq

Form		|*g*_*x*_|	|*g*_*y*_|	|*g*_*z*_|
NHaseBA	Exp.	2.14	2.29	1.97
	ORCA	2.07	2.11	2.01
NHaseAq	Exp.	2.13	2.21	1.99
	ORCA	2.07	2.10	2.01

**Fig. 13:**
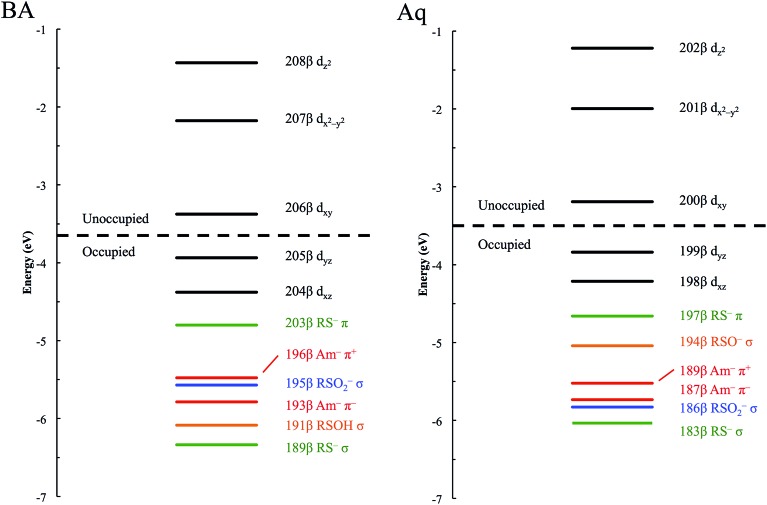
β MO energy diagram for NHaseBA (left) and NHaseAq (right). Metal d MOs are in black, thiolate MOs in green, sulfinate MOs in blue, sulfenate MOs in orange, and amidate MOs in red.

**Fig. 14:**
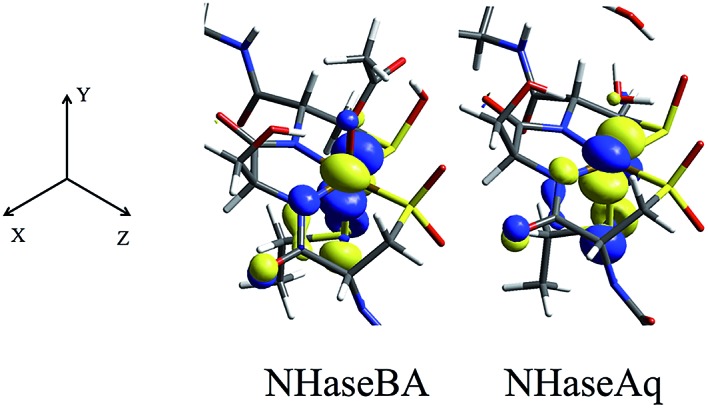
NHase β LUMOs. Isosurface contour plots of the DFT-calculated, d_*xy*_ β MOs of NHaseBA (center) and NHaseAq (right), along with the coordinate system for both models (left).

The minority spin β molecular orbital energy diagrams for NHaseBA and NHaseAq are given in Fig. 13 left and right, respectively. Contour plots for all MOs in Fig. 13 are supplied in the ESI.† For both forms of the enzyme the calculated half-occupied t_2g_ orbital is d_*xy*_ (perpendicular to the amidate N–sulfenate S axis) 206β for NHaseBA and 200β for NHaseAq in Fig. 14 (left and right, respectively). For both MOs d_*xy*_ interacts most strongly with the Cys-S^–^ π orbital, but also interacts with the amidate π orbitals *trans* to the sulfenate group. The thiolate ligand of NHase is therefore the strongest π donor ligand in this enzyme. The implications of this strong donor bonding interaction will be considered further in the Discussion. From Fig. 13, the unoccupied σ* orbitals are d_*x*^2^–*y*^2^_ and d_*z*^2^_, with the d_*z*^2^_ orbital highest in energy. These two dσ orbitals are split by ≈6000 cm^–1^ in NHaseBA and ≈6300 cm^–1^ in NHaseAq. The sulfinate and its *trans* amidate both overlap with d_*z*^2^_ and are therefore the strongest σ donors in the active site.

### DFT and TD-DFT calculations: dπ → dσ* LF and CT transitions

From Fig. 13, closest in energy to the Fe d orbital manifold for both active site models is the Cys-S^–^ π orbital, as would be expected from its π bonding nature and the relatively low electronegativity of the S valence 3p orbitals. For the NHaseBA model, the next highest-energy MO is the diamidate in-phase, π^+^, combination, with the out-of-phase, π^–^, combination at lower energy. The Cys-SO_2_^–^ σ orbital (Fig. 9) is sandwiched between the π^+^ and π^–^ MOs. For NHaseBA, below π^–^ lies the Cys-SOH σ and the Cys-S^–^ σ MOs are lowest in energy.

For the NHaseAq model, the next MO below the thiolate π is the Cys-SO^–^ σ donor orbital. The removal of the proton from the coordinated sulfenic acid significantly raises the energy of this orbital, reflected in the low energy Cys-SO^–^ σ → dσ* CT transition of the Co^III^ sulfenate complex that was eliminated upon protonation of the sulfenate group (*vide supra*). The high energy of this Cys-SO^–^ MO has implications for this ligand's capacity as a nucleophile (*vide infra*). Below the Cys-SO^–^ σ lie the amidate π^+^ and π^–^ MOs followed by the Cys-SO_2_^–^ σ and Cys-S^–^ σ MOs. Thus the calculated energy ordering of the different S ligands reasonably parallels the order determined experimentally for the model complexes.

**Fig. 15:**
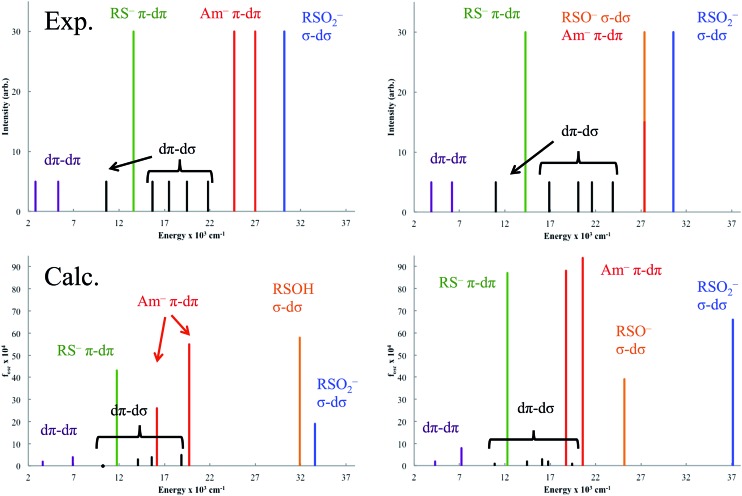
Experimental (top) and TD-DFT (bottom) transitions for NHaseBA (left) and NHaseAq (right). Lines are colored purple for dπ → dπ, black for dπ → dσ, green for Cys-S^–^ π → dπ, red for amidate π → dπ, orange for Cys-SOH/-SO^–^ σ → dσ and blue for Cys-SO_2_^–^ σ → dσ. The transition at ≈27 000 cm^–1^ in the upper-right plot is marked half red, half orange to denote the overlap of the amidate dπ → dπ and Cys-SO^–^ σ → dσ transitions.

The experimentally-determined and TD-DFT-predicted transitions for NHaseBA and NHaseAq are given in Fig. 15 left and right, respectively. The predicted dπ → dπ* transitions for NHaseBA are at 3600 and 6900 cm^–1^, similar in energy to the experimentally determined (from Taylor analysis and NIR MCD data) values of 2800 and 5300 cm^–1^. The same is true for NHaseAq, with calculated values of 4300 and 7200 cm^–1^ compared to experimental values of 3900 and 6200 cm^–1^. The calculations correctly predict the increase in the dπ → dπ LF transition energies upon replacing the butyrate ligand with water, and the values of |*V*/2*Δ*| determined from these TD-DFT transitions (0.31 for NHaseBA and 0.25 for NHaseAq) agree well with the experimental values (0.32 and 0.23, respectively). The calculated dπ → dσ* LF energies for NHaseBA range from 10 200 to 18 900 cm^–1^, which compares reasonably well to the experimental range of 10 600 to 21 800 cm^–1^. The corresponding dπ → dσ* transition energies for the NHaseAq model range from 10 900 to 19 400 cm^–1^, similar to the experimental transitions of 11 000 to 23 900 cm^–1^. The calculated dπ → dσ* transitions shift up in energy upon replacing butyrate with water, as is observed experimentally, which indicates an increase in 10Dq, reflecting replacement of the π donor butyrate in NHaseBA by water.

The calculated Cys-S^–^ π → dπ CT transitions are at 11 800 cm^–1^ for NHaseBA and 12 300 cm^–1^ for NHaseAq. These values are in the range of those determined experimentally (13 600 cm^–1^ for NHaseBA and 14 300 cm^–1^ for NHaseAq), and the increase in transition energy upon substituting a water ligand for butyrate is correctly predicted. The calculated amidate π → dπ* transition energies for NHaseBA (16 200 and 19 700 cm^–1^) and NHaseAq (18 800 and 20 600 cm^–1^) are significantly lower than their experimental counterparts (≈26 000 cm^–1^ for NHaseBA and ≈27 000 cm^–1^ NHaseAq), perhaps reflecting a large self-interaction error for the anionic ligands.^38^ The models do, however, correctly predict that the amidate-based transitions of NHaseAq are shifted up in energy relative to those of NHaseBA. For NHaseBA the calculated Cys-SOH σ → dσ and Cys-SO_2_^–^ σ → dσ CT transitions are 31 900 and 33 600 cm^–1^, respectively. From the model studies it was predicted that the Cys-SOH-based transition would lie to higher energy, but in this system the Cys-SO_2_^–^ transition is to the higher energy d_*z*^2^_ orbital while the Cys-SOH transition is to the lower energy d_*x*^2^–*y*^2^_ orbital. In the TD-DFT calculation of NHaseAq, the Cys-SO^–^-based transition at 25 200 cm^–1^ is 6000 cm^–1^ lower in energy relative to that of Cys-SOH in NHaseBA. This is consistent with the experimental Cys-SOH σ → dσ* CT transition of NHaseBA being unobserved and the broad, intense transition of NHaseAq at 27 500 cm^–1^ arising from the overlap of the amidate π → dπ* and Cys-SO^–^ σ → dσ* CT transitions. The lower energy CT transition for sulfenate *vs.* sulfenic acid indicates an increase in the sulfenate frontier MO energy with deprotonation and a consequent increase in its nucleophilic character. While TD-DFT calculations of the CT excited states are shifted relative to experiment, their ordering and energy shifts between enzyme forms are consistent with experiment. Also, the functional used predicts the correct LS ground state and from the Taylor analysis above the ground state parameters are well described. The functional (BP86) and basis set employed above were thus used to evaluate possible mechanisms of nitrile hydration by this unusual LS Fe^III^ active site.

### Reaction coordinate calculations

**Fig. 16:**
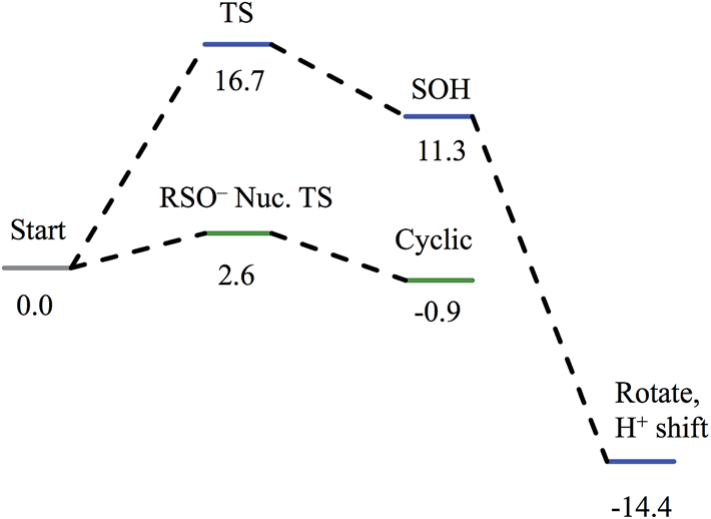
Reaction coordinates for attack on coordinated nitrile by water (blue) and attack on coordinated nitrile by sulfenate (green). All energies are in kcal mol^–1^.

**Fig. 17:**
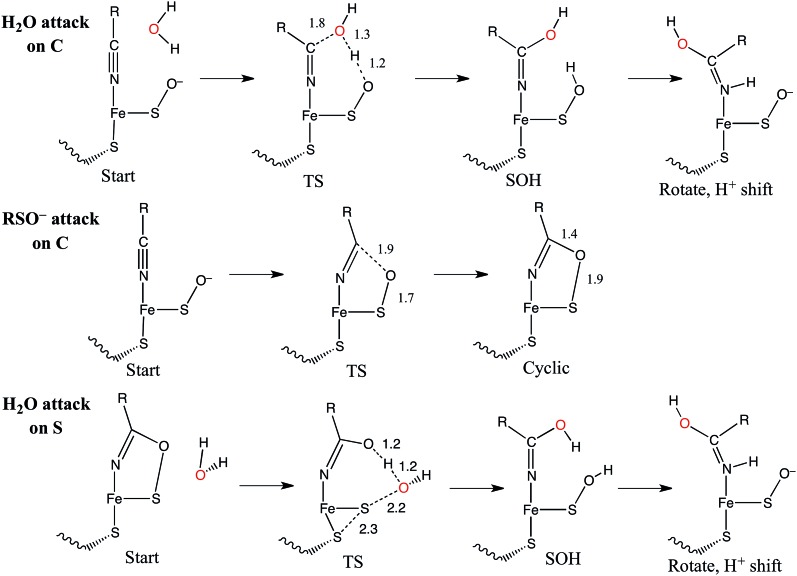
Structures listed for the reaction coordinates of Fig. 16 and 18. All distances are in Å.

Nucleophilic attack on coordinated acetonitrile by water with the sulfenate group acting as a proton acceptor was explored as described previously.^20^ This is shown in blue in the energy diagram in Fig. 16, with corresponding structures in Fig. 17, top. At the transition state, the proton is approximately equidistant between the water and sulfenate oxygens. The electronic energy of the transition state lies 16.7 kcal mol^–1^ above the ground state, similar to the value of 20.2 kcal mol^–1^ obtained previously.^20^ Complete transfer of the proton to the sulfenate group leads to a coordinated amidate tautomer structure 11.3 kcal mol^–1^ above the starting structure, labeled as ‘SOH’ in Fig. 16. Rotation of the amidate tautomer around the Fe–N axis leads to the abstraction of the proton of sulfenic acid by amidate tautomer N, generating the coordinated amide tautomer at –14.4 kcal mol^–1^ relative to the starting coordinated nitrile structure. Tautomerization/dissociation of the product amide and regeneration of the active form of NHase leads to a net energy change of –10.4 kcal mol^–1^. For the reaction of two water molecules with free acetonitrile, one water acting as nucleophile and the other as proton acceptor,^20^ the electronic energy of the transition state is 27.4 kcal mol^–1^, or approximately 11 kcal mol^–1^ higher than for water attack on NHase LS Fe^III^-coordinated nitrile. Thus the combination of activation of nitrile by coordination to metal and the better nucleophilic character of sulfenate relative to water lowers the energy barrier by ≈11 kcal mol^–1^.

Alternatively, the sulfenate group can act as the nucleophile towards the coordinated nitrile to form a cyclic intermediate.^21^ The reaction coordinate and associated structures for this process are also shown in Fig. 16 and 17, center, respectively. The process of direct attack by sulfenate on nitrile (shown in green in Fig. 16) has a barrier of only 2.6 kcal mol^–1^, significantly lower than the barrier for nucleophilic attack on nitrile by water (16.7 kcal mol^–1^, blue in Fig. 16). The cyclic species formed by attack of the sulfenate is effectively isoenergetic with the starting structure.

**Fig. 18:**
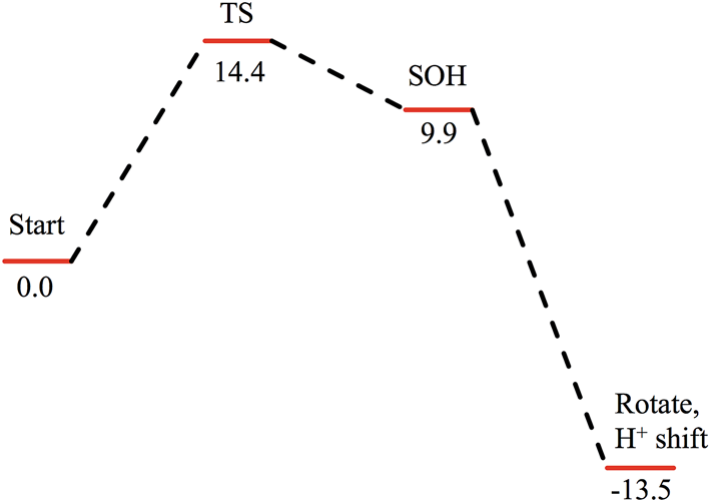
Reaction coordinate for nucleophilic attack by water on the S atom of the cyclic structure. Species defined at bottom of Fig. 17, with zero energy referenced to the cyclic intermediate at –0/9 kcal mol^–1^ in Fig. 16. All energies are given in kcal mol^–1^.

**Fig. 19:**
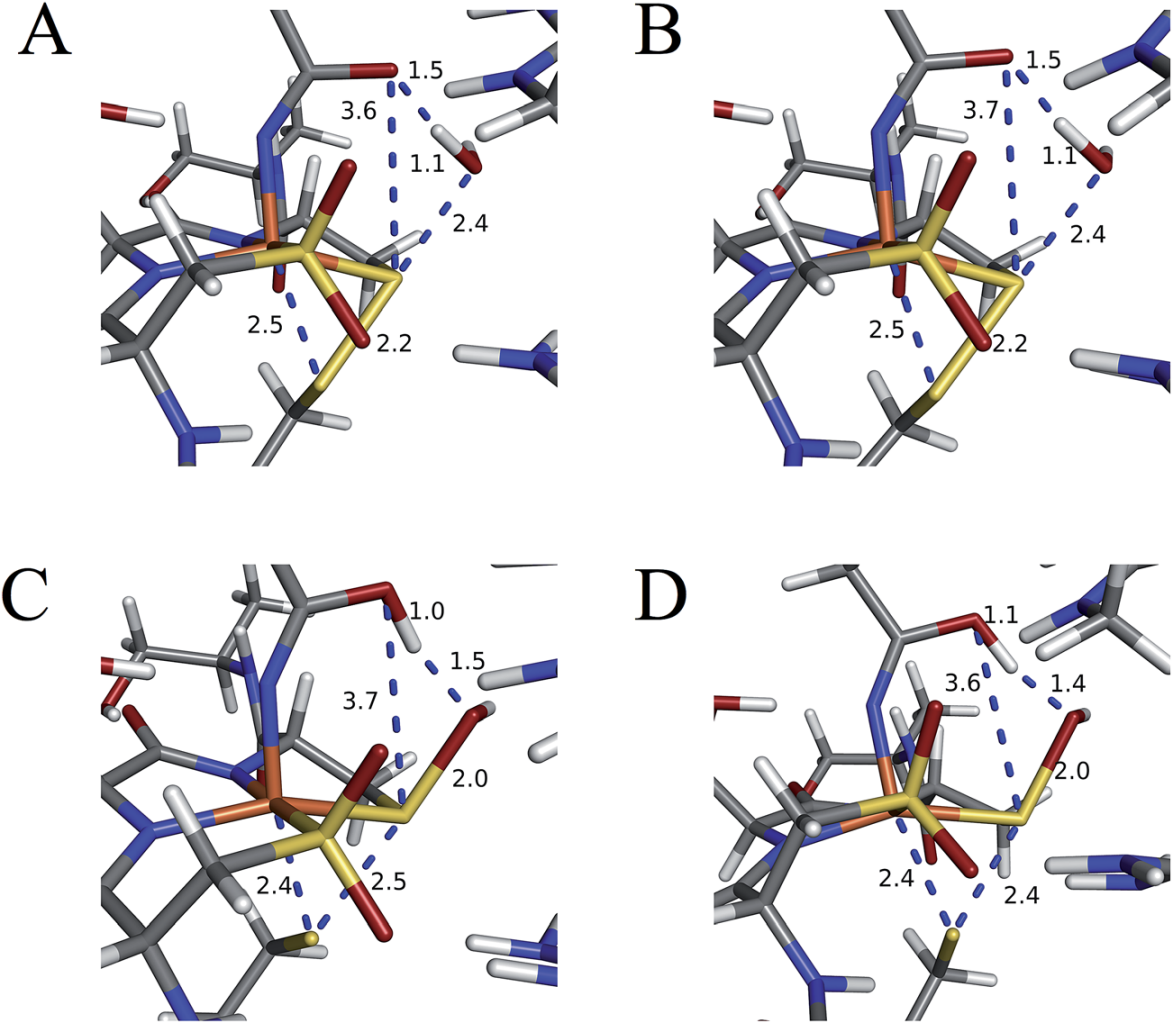
Structures of (A) linear transit species with a water O–cyclic intermediate S distance of 2.4 Å, (B) IRC step towards reactants with this same distance, (C) SOH species, and (D) IRC step towards products. All distances are in Å.

Subsequent attack by water on the cyclic species can occur at the (formerly) nitrile carbon or at the (formerly) sulfenate sulfur. Attempts to produce a transition state for attack at the nitrile carbon led only to cleavage of the sulfenate–O bond, with no transfer of a proton from attacking water to the O now bound to C. Also, the energy was approximately 28 kcal mol^–1^ higher than the starting structure for a water O–nitrile C distance of 1.8 Å. Nucleophilic attack by water on the C of the cyclic species was therefore deemed unfeasible. However, nucleophilic attack by water on the S of the cyclic structure did lead to cleavage of the sulfenate S–O bond and generation of a coordinated amidate tautomer (SOH) similar to the nucleophilic attack by water on (uncyclized) coordinated nitrile. The reaction coordinate for this attack at S is given in Fig. 18, and structures of the transition state and end point before and after amidate tautomer rotation are given in Fig. 17, bottom. Sulfenate S–O bond-breaking occurs relatively early in the reaction coordinate: from a linear transit study, at a water O–cyclic intermediate S distance of 2.6 Å the cyclic intermediate O–cyclic intermediate S distance has increased to 2.5 Å, and at the transition state this latter distance has increased to 3.6 Å, as the attacking water is forming a bond to the cyclic intermediate S and transferring a proton to the cyclic intermediate O. Points along the IRC (compared with the SOH product and the structure for a water O–cyclic intermediate S distance of 2.4 Å from the linear transit, Fig. 19) indicate that the transition state proceeds towards the reactants and products. The barrier for attack at the S of the cyclized species is 14.4 kcal mol^–1^, or 2.3 kcal mol^–1^ lower than that of attack at coordinated, uncyclized C in Fig. 16. At the transition state (one imaginary frequency, confirmed to be on the IRC), the proton transfer is similar to that of an attack on uncyclized nitrile. Interestingly, in the transition state for attack on the S of the cyclized species, the sulfenate S–axial thiolate S distance has shortened to 2.3 Å, indicating some bonding between these atoms (a Mayer bond order of 0.53 between the sulfenate S–axial thiolate S was calculated, whereas the S–S bond in dimethyl disulfide has a calculated Mayer bond order of 1.61). This is similar to a recent computational study proposing a mechanism involving nucleophilic attack by the axial thiolate upon the sulfur of the cyclic intermediate, resulting in the formation of a disulfide bond.^22^ In the computed mechanism at the bottom of Fig. 17 and 18 the S–S interaction aids in stabilizing the transition state and lowering the barrier for this reaction step involving H_2_O attack on the cyclic S. After amidate tautomer rotation and proton shifting the S–S distance increases to 3.1 Å and the bonding interaction is eliminated. The nature of the activation of the coordinated nitrile and the cyclic structure are explored below.

## Conclusions

**Fig. 20:**
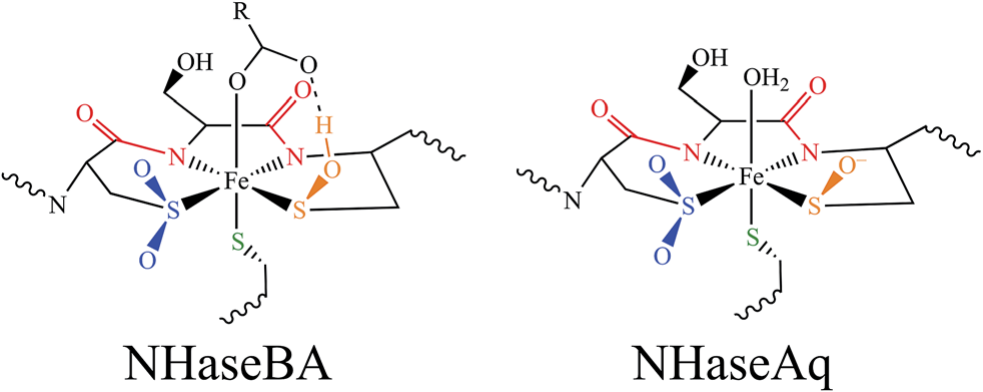
Spectroscopically determined active site structures of NHaseBA (left) and NHaseAq (right).

The EPR and UV-Vis MCD spectra of NHaseBA and NHaseAq in this study have shown that the LS Fe^III^ site of NHase, which has a protonated sulfenate group when butyrate is coordinated to metal, has a deprotonated sulfenate group and coordinated water ligand in the active form as shown in Fig. 20. For both forms, the active site (including the positively charged βArg56 and βArg141 that are H-bonding to the sulfenate and sulfinate ligands) has zero net charge therefore the NHase active site maintains charge neutrality and the coordinated sulfenate can easily change protonation state at functional pHs (here determined to have a p*K*_a_ of 6.1 and deprotonated in the active form of the site). From the EPR, UV-Vis LT Abs and UV-Vis/NIR MCD spectroscopic data coupled to DFT calculations the highest energy, half-occupied dπ orbital for both NHase forms is strongly π bonding to the cysteine thiolate ligand, and the sulfenate ligand possesses a high-energy occupied σ_IP_ MO with significant O character (Fig. 9), implying significant nucleophilic capability for this ligand. These spectrally evaluated structural and electronic properties of the NHase active site provide insight into its reactivity.

**Fig. 21:**
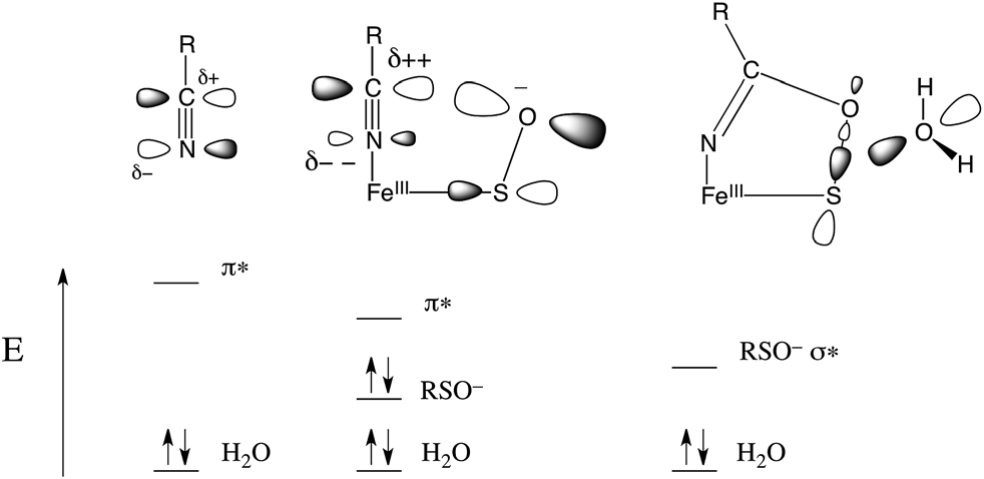
Donor/acceptor orbitals for free nitrile and water (left), coordinated nitrile and water (center) and the coordinated cyclic species and water (right).

Using EPR, NIR MCD, and UV-Vis LT-Abs and MCD spectroscopic data, computational models of NHaseBA and NHaseAq were developed which qualitatively reproduce the spectroscopic features and geometric and electronic structures of the two enzyme forms. These findings show that the computational models provide a reasonable basis for evaluating possible NHase catalytic mechanisms. The five negatively charged protein-derived ligands combined with the nearby positively charged βArg56 and βArg141 residues lead to a relatively high p*K*_a_ for the bound H_2_O, allowing its displacement and nitrile substitution. Upon coordination to the LS Fe^III^ the nitrile is activated for nucleophilic attack: (a) the C≡N bond becomes more polarized such that the C atom has an increased electrostatic interaction with the nucleophile, (b) the nitrile frontier π* LUMO has more C character, leading to better overlap with the HOMO of the nucleophile, and (c) the energy of the nitrile π* MO is decreased, leading to better covalent interaction. DFT calculations on free acetonitrile and acetonitrile bound to the Fe^III^ of NHase show that upon coordination to LS Fe^III^ the positive charge on the nitrile C increases from +0.12 to +0.21, the C character in the acetonitrile π* LUMO increases from 53% to 59% and the energy decreases by ≈0.2 eV. This activation is shown in Fig. 21, left and center.

The NHase active site also possesses a good internal nucleophile in the coordinated sulfenate ligand. This is shown in Fig. 21, center. The occupied sulfenate S–O σ_IP_ MO may act as a donor and is higher in energy than the water MO by ≈1.6 eV. It is negatively charged, and is well-oriented for overlap with the nitrile π* acceptor orbital. Sulfenate can therefore attack the nitrile C to form the cyclic species shown in Fig. 21, right. This cyclic species has an unoccupied sulfenate S–O σ* FMO which is ≈3 eV lower than the π* orbital of the uncyclized coordinated nitrile, leading to favorable attack by water at S and a lower reaction barrier than for attack by water on the coordinated, uncyclized nitrile.

Alternatively, the axial thiolate could act as a nucleophile as previously predicted.^22^ In this reported mechanism, the barriers for formation of the cyclic intermediate and the disulfide intermediate are the largest and of similar energy, indicating that both should be observed over the course of enzyme turnover. Holz and coworkers reported stopped flow data for an Fe NHase enzyme with substoichiometric nitrile reactant, which showed intermediates with a blue shift in the Cys-S^–^ π → dπ CT transition of approximately 1100 cm^–1^.^39^ TD-DFT calculations on the cyclic intermediate both in this work and on the structure in ref. 22 showed Cys-S^–^ π → dπ CT transitions approximately 4000 cm^–1^ higher in energy than NHaseAq, whereas the disulfide intermediate of ref. 22 showed no transition in the ≈12 000–18 000 cm^–1^ region. As the ≈14 300–15 400 cm^–1^ band does not disappear over the course of the stopped-flow experiment, the presence of a disulfide intermediate does not appear likely.

The ligand set and spin state of NHase are unusual relative to the other mononuclear non-heme iron enzymes. Most are ferrous enzymes that activate O_2_ and utilize histidine, glutamate, and aspartate residues to coordinate the metal and facilitate the redox reaction. In the intradiol dioxygenases an Fe^III^ with Tyr ligands is active, but this is high spin and this spin state and its change along the reaction coordinate are important in activating the singlet substrate for the spin-forbidden reaction with ^3^O_2_.^40^ In order to determine the contribution of the low spin state on the nitrile-bound form of NHase, geometry optimizations were performed on models of this complex with *S* = 3/2 and 5/2 ground states. Whereas the nitrile N–Fe bond length in the *S* = 1/2 spin state is ≈1.9 Å, this length is ≈2.6 Å in the *S* = 3/2 state and ≈2.4 Å in the *S* = 5/2 state, indicating that the bonds are very weak to nonexistent. Indeed, the nitrile N–Fe bond dissociation energies for the *S* = 1/2, *S* = 3/2, and *S* = 5/2 forms are +6.6, –2.3, and –7.0 kcal mol^–1^ respectively, indicating that the low spin state of NHase is required to assist in the coordination and activation of nitrile substrates.

In order to explore the effects of the NHase ligands on the spin state and activity of the enzyme, DFT calculations were performed on active site models with unoxidized Cys residues, as well as with two His or two acetate ligands replacing the backbone amidates of the WT site. The active site model with all unoxidized thiolates was found to not have an *S* = 1/2 ground state; the *S* = 3/2 ground state was lower in energy by 14.2 kcal mol^–1^. Geometry optimization of this *S* = 3/2, structure leads to dissociation of the exogenous ligand and a 5C form that would not be catalytic. Alternatively, the amidate ligands of NHase are not critical to maintaining the low-spin active site. The *in silico* results with the amidates replaced with weaker donors indicate better activation of coordinated nitriles for nucleophilic attack (*i.e.* these form stronger Fe^III^–nitrile bonds). Nature may have selected this deprotonated amide ligand set for its rigidity and chelate ring for orienting the sulfenate ligand for attack on the C of the coordinated nitrile.

Finally, consistent with the EPR *g* value analysis and LF MCD, the DFT calculations indicate that the axial Cys thiolate is the strongest π donor in the NHase coordination sphere and controls the orientation of the half-occupied dπ orbital. This strong π donor *trans* to coordinated nitrile would not assist in its activation, but may serve to increase both H_2_O and product lability from the LS Fe^III^ site, as was observed by Kovacs *et al.* for NHase model complexes.^41^ This increased lability would be especially important for NHase forms that utilize low-spin Co^III^, which generally undergoes very slow ligand exchange. The presence of a weak interaction between the thiolate and sulfenate S atoms in the transition state for nucleophilic attack on the S of the cyclic species (Fig. 17, bottom, TS) also indicates that the axial thiolate may serve to lower the energy barrier for nitrile hydrolysis.

In summary, our spectroscopic results have provided new insight into the geometric and electronic structure of NHase, which activates nitriles by coordination to a LS Fe^III^ and contains a sulfenate group that acts as a good nucleophile oriented well for this attack. These spectroscopically-calibrated computational results show that the cyclic intermediate that would be formed in this reaction is activated for nucleophilic attack by water at the S atom leading to formation of the amide product and regeneration of the active site sulfenate. Nature has selected an unusual set of ligands for this enzyme to ensure that the low-spin state necessary for nitrile binding is maintained, and that a rigid chelate ring is present, which properly orients the frontier MO of the sulfenate group for nucleophilic attack on the bound substrate.

## Supplementary Material

Supplementary information
