# Supplementary material for: Spectroscopic and computational studies of nitrile hydratase: insights into geometric and electronic structure and the mechanism of amide synthesis
Source: Chem Sci. 2015 Jul 30;6(11):6280–94. doi: 10.1039/c5sc02012c (PMC4618400; doi:10.1039/c5sc02012c)
Supplement: Supplementary file 1 [file SC-006-C5SC02012C-s001.pdf]

This journal is © The Royal Society of Chemistry 2015

Electronic Supplementary Information

Materials and Methods

Sample Preparation.

NO-bound NHase was prepared as described previously.[1] Samples were exchanged into HEPES buffer (100 mM, pH 7.5) containing 40 mM butyric acid in the dark to concentrations of 2-4 mM. Samples were placed on ice and exposed to light from a 400 watt tungsten lamp at a distance of 30.5 cm for 15 minutes. NHaseAq was prepared by exchanging NHaseBA samples into butyric-acid-free buffer in an anaerobic glovebox under an atmosphere of N_2. Oxidized NHase (NHaseOx) was prepared from NHaseAq by allowing it to sit out in air until the EPR spectrum remained constant with time. NHaseAq and NHaseOx samples at pH 8.5 and 7.5 were prepared in 100 mM HEPES buffer, while samples at pH 6.5 contained 100 mM MES buffer. Samples of NHaseBA and NHaseAq were exchanged into D_2O solutions of HEPES buffer (100 mM, pD 7.5, plus 40 mM butyric acid for NHaseBA) and then saturated with sucrose (as a glassing agent) to Fe concentrations of 1-2 mM for the collection of low-temperature UV-vis absorption, near-infrared (NIR) MCD, and UV-Vis MCD spectra. EPR spectra of NHase samples taken with and without sucrose were identical, indicating that sucrose did not perturb the active site.

Spectroscopic Methods.

X-band EPR spectra were collected on a Bruker EMX spectrometer using a Bruker ER 041XG/ER microwave bridge and an ER4116DM cavity. Sample temperatures were maintained at 77 K with a liquid nitrogen finger dewar. NHaseAq spectra were simulated using the SimPOW6 program.[2] NIR (600–2000 nm) MCD spectra were collecteded on a JASCO J-730 spectropolarimeter with a liquid N_2-cooled InSb detector and an Oxford Instruments SM4000-7T superconducting magnet. Vis-UV (300–900 nm) MCD data were collected on a JASCO J-810 spectrapolarimeter with an extended S-20 photomultiplier tube and an Oxford Instruments SM4000-7T superconducting magnet. MCD spectra were corrected for the natural CD and zero-field baseline effects by averaging the magnitudes of the positive and negative field data. Low-temperature UV-Vis (250-900 nm) absorption

spectra were collected on a Cary 17 spectrophotometer using a Janis Research Super Vari-Temp liquid helium cryostat.

Computational Methods.

The active sites of NHaseBA and NHaseAq were modeled using the crystal structures with PDB IDs 2CZ1 and 2CYZ, respectively. The active sites were truncated and key carbon atomic coordinates frozen as described in the supporting information. For NHaseBA structures, the butyrate ligand was substituted with acetate. The Co^{III} complexes $[(en)_2Co(SCH_2CH_2NH_2)\text{-}N,S]^{2+}$, $[(en)_2Co(S(O)CH_2CH_2NH_2)\text{-}N,S]^{2+}$ and $[(en)_2Co(S(O)_2CH_2CH_2NH_2)\text{-}N,S]^{2+}$ were modeled using Cambridge Structural Database entries MEAENC, SEANCO, and SEAENC, respectively. The $[(en)_2Co(S(OH)CH_2CH_2NH_2)\text{-}N,S]^{3+}$ complex was modeled by adding a proton to the sulfenate group of SEANCO. LS Fe^{III} models of these complexes were created by substituting Fe for Co. DFT and time dependent DFT (TD-DFT) calculations were performed on all computational models using the Gaussian 09 software package[3] under tight convergence criteria. Geometry optimizations and single point calculations of the NHase active site models were performed with the spin-unrestricted BP86, BP86 with 10% Hartree-Fock mixing, and B3LYP functionals for the S=1/2, S=3/2, and S=5/2 states. TD-DFT calculations were performed with the BP86 functional. Optimizations, single point calculations and TD-DFT calculations for the Co model complexes were performed with the B3LYP functional (calculations using the BP86 functional predicted the R-SO$^-$ $\sigma\rightarrow d\sigma^*$ transition to be below the $d\pi\rightarrow d\sigma$ transitions, which is not experimentally observed, *vide infra*). For the active site models, the triple–ξ basis set 6–311G* was used to describe the Fe and S atoms, the O atoms of the sulfenate/sulfenic groups, the N, C and O atoms of the deprotonated amide groups, and the atoms of the acetate carboxylate group in NHaseBA or the O of coordinated water in NHaseAq. All other atoms were modeled using the double–ξ basis set 6–31G*. For comparisons of energies (e.g., comparing spin states), the triple–ξ basis set 6–311G* was used for all atoms, and for energies of reaction coordinate species the D3 version of Grimme's dispersion was included.[4] Frequency calculations were performed for all transition states, which were found to have one large imaginary

frequencies and smaller imaginary frequencies of less than ≈ -40 cm^{-1} in value associated with atoms frozen during geometry optimization. For the Co complex models, the triple–ξ basis set 6–311G* was used to describe the Co, Fe, S, O, and N atoms, whereas all C and H atoms were modeled with the double–ξ basis set 6–31G*. EPR g values for the NHaseBA and NHaseAq optimized structures were calculated with the ORCA 2.9 computational package[5] using the same functional and basis sets as used in the Gaussian09 calculations. Solvation effects were included in all calculations through the use of the polarized continuum model (PCM)[6] with a dielectric constant ε = 4.0 for active site models and ε = 80.0 (water) for the Co complex models. All molecular orbitals were visualized with the LUMO program[7] at an isovalue of 0.05. Mulliken population analyses were performed with the QMForge program.[8]

NHase computation Model Constraints

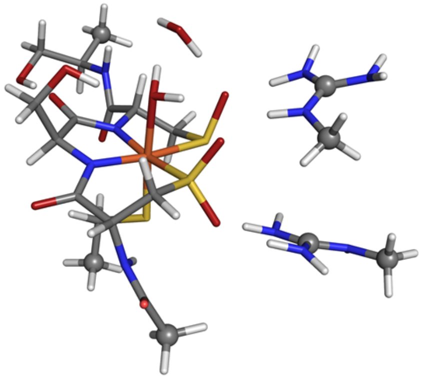

Figure S1. Example computation structure. C atoms whose coordinates were frozen during geometry optimization are shown as spheres.

Modeling of EPR values

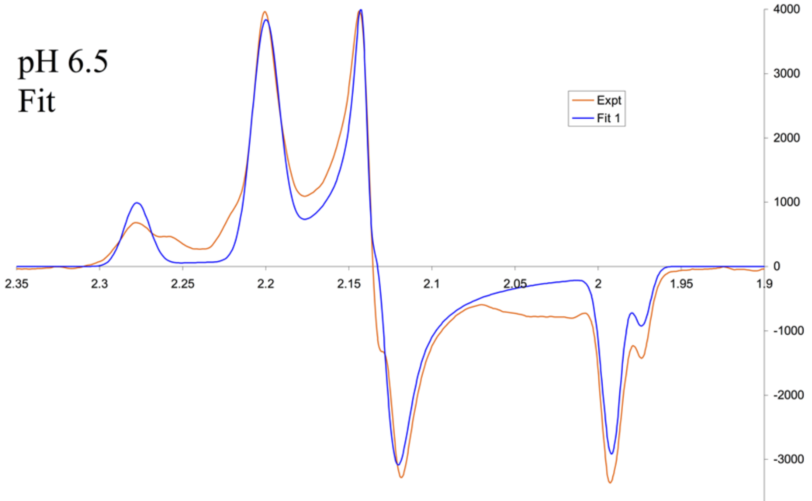

Fit1	Abund %	g1	g2	g3
Mid	72	2.20028	2.12952	1.99192
Low	28	2.27774	2.13953	1.97357

Figure S2. Experimental and fitted EPR spectra of NHaseAq at pH 6.5.

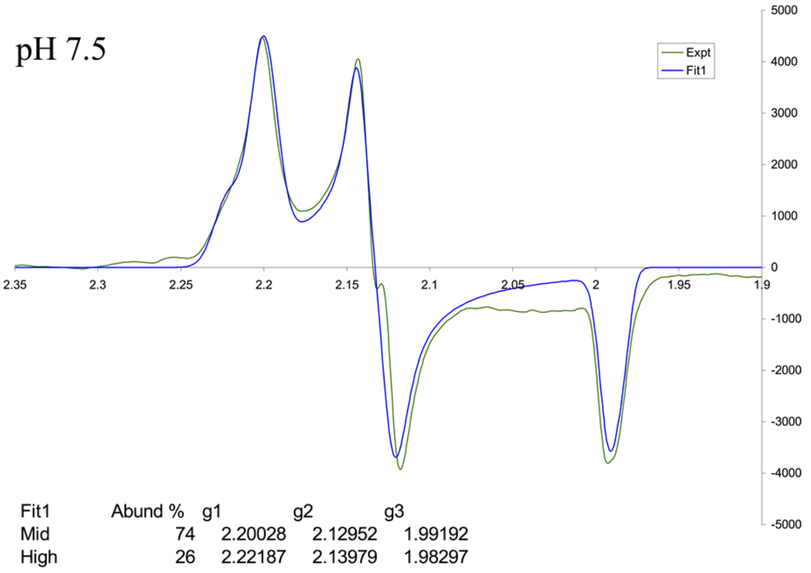

Figure S3. Experimental and fitted EPR spectra of NHaseAq at pH 7.5.

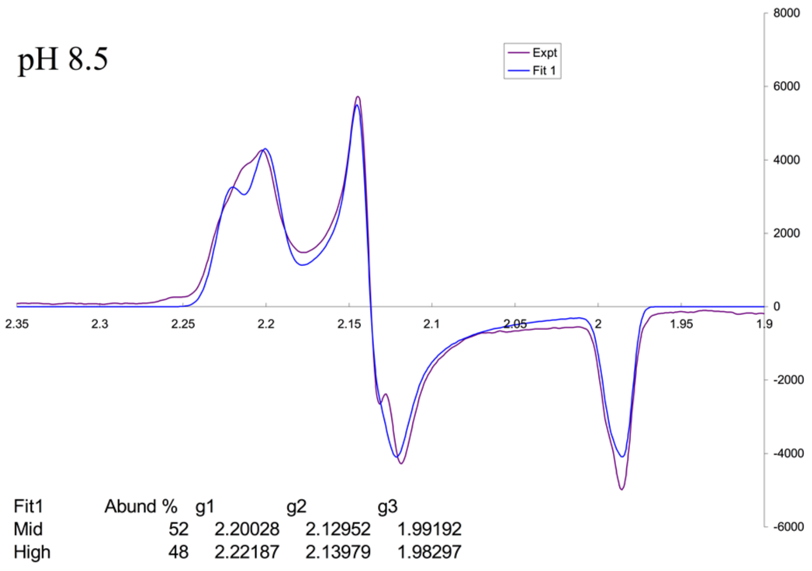

Figure S4. Experimental and fitted EPR spectra of NHaseAq at pH 8.5.

Calculated Molecular Axes

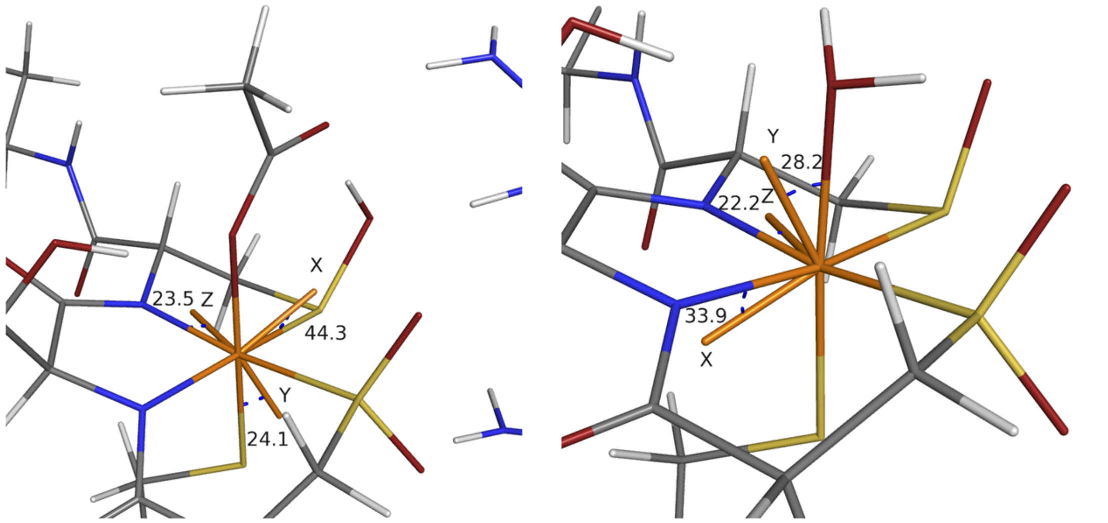

Figure S5. Calculated g tensor axes and angles to nearest bond for NHaseBA (left) and NHaseAq (right). All angles are in degrees.

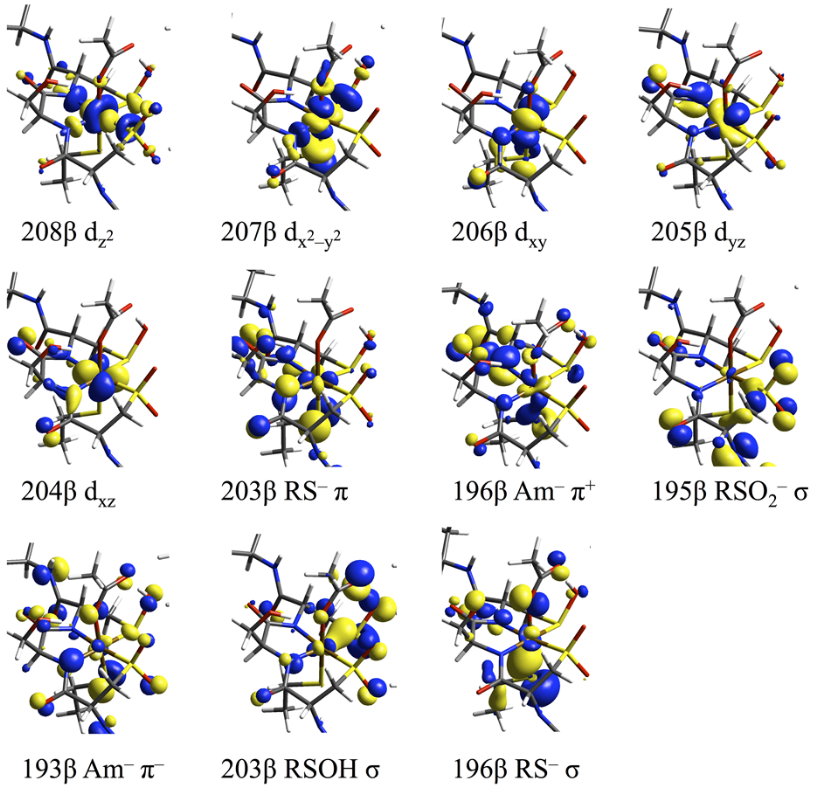

Figure S6. MO contours for NHaseBA.

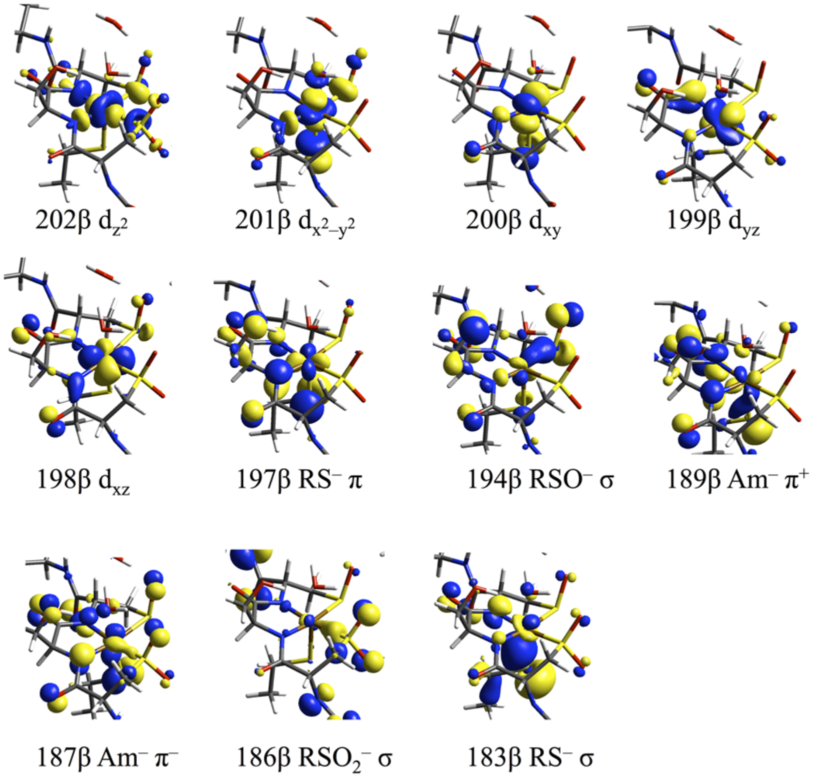

Figure S7. MO contours for NHaseAq.

Table S1. Optimized coordinates for $[(en)_2Co(SCH_2CH_2NH_2)$-*N,S*$]^{2+}$.

Co	0.125472	-0.003368	0.038862
C	-2.389853	-1.073509	1.113935
C	-2.802777	-0.970783	-0.342526
C	0.649532	2.728327	-0.628459
C	0.549825	2.694763	0.886497
C	2.333029	-1.766609	0.570071
C	2.110648	-1.823568	-0.932083
H	-2.641811	-0.153788	1.650156
H	-2.885412	-1.912441.618028	
H	0.856711	1.258252	-2.079993
H	2.081338	1.2891 -0.998815	
H	-2.666249	-1.931672	-0.851762
H	-3.858477	-0.693588	-0.418911
H	-0.157506	1.296311	2.248843
H	-1.280263	1.778388	1.150048
H	1.372571	3.476691	-0.968975
H	-0.322631	2.953447	-1.075768
H	2.560403	0.255791	0.914684
H	1.686985	-0.486433	2.073301
H	1.539276	2.561878	1.33764
H	0.117211	3.619588	1.282408

H	0.102357	-2.302259	-1.077797
H	0.557282	-1.190127	-2.172882
H	1.765098	-2.556557	1.073835
H	3.390689	-1.893765	0.823951
H	-0.699873	-2.239744	0.957576
H	-0.618454	-1.168225	2.181298
H	2.745776	-1.093287	-1.445587
H	2.339001	-2.815693	-1.335352
N	1.055636	1.360048	-1.073878
N	-0.290351.51713	1.250418	
N	1.821132	-0.449334	1.051759
N	0.684938	-1.459531	-1.187227
N	-0.905372	-1.256944	1.194132
S	-1.789755	0.332984	-1.161641

Table S2. Optimized coordinates for $[(en)_2Co(SOCH_2CH_2NH_2)$-*N,S*$]^{2+}$.

S	-1.704743	-0.400491	-1.014344
N	1.303263	-1.104691	-1.241289
N	0.518548	1.64166	-1.093496
N	-0.101653	-1.538843	1.237422
C	3.156613	-0.249896	0.085681
C	-0.321702	2.648721	0.949595
C	-1.97868-2.039744	-0.226591	
H	3.309368	-1.838394	-1.388568
H	1.492787	-0.556976	-2.09619
H	3.430611	0.520601	-0.644284
H	2.304006	1.279546	1.185673
H	0.07144	3.729414	-0.900062
H	1.4992	1.953049	-1.114707
H	0.667292	2.869778	1.364972
H	-1.682451.105545	1.085384	
H	-1.640514	-2.933615	1.728211
H	0.471909	-2.358904	0.991197
H	-1.404231	-2.779552	-0.800827
Co	0.275536	0.020035	0.048021
O	-2.760422	0.537241	-0.344276
N	2.076892	0.302827	0.954564
N	-0.670722	1.236703	1.27433
C	2.61261	-1.478231	-0.624296
C	-0.320013	2.76413	-0.564309
C	-1.532637	-1.969219	1.218804
H	2.437639	-2.293202	0.086618
H	0.843866	-1.959025	-1.590526
H	4.049151	-0.494073	0.671132
H	2.092313	-0.180605	1.865035
H	-1.332766	2.636443	-0.953216
H	0.278792	1.480408	-2.083845
H	-0.509468	1.061305	2.276192
H	-2.123422	-1.227003	1.763465
H	0.169034	-1.331293	2.211945
H	-3.043618	-2.271212	-0.326195
H	-1.042594	3.338598	1.401723

Table S3. Optimized coordinates for $[(en)_2Co(SOHCH_2CH_2NH_2)\text{-}N,S]^{3+}$.

S -1.675856 -0.434201 -1.051614
N 1.273241 -1.17572-1.215021
N 0.60986 1.603428 -1.090234
N -0.143086 -1.513375 1.268898
C 3.15259 -0.298231 0.048867
C -0.216907 2.693689 0.9102
C -2.059324 -1.991228 -0.17775
H 3.283312 -1.873299 -1.442209
H 1.41821 -0.672438 -2.106964
H 3.39953 0.484538 -0.676993
H 2.31075 1.184483 1.226137
H 0.166814 3.69344 -0.982121
H 1.594634 1.909033 -1.085349
H 0.774796 2.946174 1.300766
H -1.560381 1.187711 1.314365
H -1.721209 -2.852753 1.775125
H 0.40314 -2.361318 1.051544
H -1.534583 -2.777006 -0.740267
Co 0.308372 0.01215 0.063166
O -2.711364 0.615934 -0.286981
N 2.071579 0.221992 0.9422
N -0.537303 1.288579 1.314317
C 2.613525 -1.529795 -0.647466
C -0.230129 2.745388 -0.605802
C -1.588389 -1.894682 1.25978
H 2.476613 -2.353023 0.062774
H 0.798764 -2.047625 -1.501531
H 4.05265 -0.521605 0.630659
H 2.083961 -0.311854 1.828155
H -1.243042.611881 -0.992742
H 0.405773 1.4276 -2.088603
H -0.243163 1.136295 2.292829
H -2.15283-1.134071.806455
H 0.124663 -1.289353 2.242941
H -3.136489 -2.166268 -0.265602
H -0.942023.38828 1.346188
H -3.529556 0.757707 -0.847456

Table S4. Optimized coordinates for $[(en)_2Co(SO_2CH_2CH_2NH_2)\text{-}N,S]^{2+}$.

Co -0.374093 0.014817 -0.145113
S 1.702343 -0.199982 0.599409
O 1.772284 -0.386686 2.07875
O 2.611063 0.842403 0.021657
N 0.1728 1.416182 -1.431696
N -0.681185 1.522919 1.116253
N 0.076619 -1.431645 -1.452119
N -1.038108 -1.297475 1.191674
N -2.286752 0.06254 -0.800356
C -0.063522 2.771947 0.56697
C -0.279396 2.751619 -0.9354
C 2.174005 -1.770288 -0.216694
C 1.54138 -1.724133 -1.596627

C	-2.53448-1.255017	1.224731	
C	-3.025252	-1.090085	-0.202822
H	3.267337	-1.815943	-0.248883
H	1.784942	-2.593366	0.393962
H	1.673871	-2.677352	-2.119156
H	1.989715	-0.934493	-2.206971
H	-0.513051	3.656641	1.028668
H	1.003062	2.754797	0.803978
H	0.275659	3.553406	-1.433255
H	-1.340509	2.867252	-1.181305
H	-2.932575	-2.164721.685653	
H	-2.835276	-0.400775	1.840916
H	-4.106867	-0.922587	-0.236202
H	-2.801663	-1.983531	-0.796296
H	-0.750396	-2.267218	0.983999
H	-2.770232	0.932859	-0.530459
H	-0.668241	-1.123481	2.13841
H	-2.370673	0.029275	-1.828057
H	-0.390152	-2.318628	-1.205744
H	-0.299565	-1.201261	-2.386575
H	1.197058	1.443659	-1.525507
H	-0.195406	1.26132 -2.383009	
H	-1.687922	1.698517	1.258719
H	-0.312904	1.347643	2.06412

Table S5. Optimized coordinates for $[(en)_2Fe(SCH_2CH_2NH_2)$-*N,S*$]^{2+}$.

Fe	0.102012	-0.007446	0.033962
C	-2.553574	-0.809757	1.096863
C	-2.904207	-0.638222	-0.371514
C	1.003421	2.655357	-0.646985
C	0.961629	2.634812	0.871555
C	2.106207	-2.065954	0.562918
C	1.848425	-2.120712	-0.932388
H	-2.721039	0.125368	1.639729
H	-3.15801-1.595398	1.566795	
H	0.995905	1.183664	-2.108744
H	2.22812 1.045031	-1.049981	
H	-2.832055	-1.593891	-0.903846
H	-3.926246	-0.264046	-0.482269
H	0.149394	1.332325	2.272351
H	-0.960101	1.974187	1.253781
H	1.796867	3.314542	-1.014891
H	0.049239	3.001039	-1.05568
H	2.613076	-0.0844 0.86057	
H	1.665859	-0.684024	2.04652
H	1.943282	2.370783	1.280633
H	0.675312	3.610351	1.278411
H	-0.203263	-2.369782	-1.030237
H	0.36159 -1.342109	-2.161705	
H	1.446216	-2.760357	1.094244
H	3.141538	-2.332022	0.801339
H	-1.005133	-2.156301	0.995584
H	-0.829774	-1.086341	2.20714

H	2.551572	-1.473834	-1.468557
H	1.956001	-3.138458	-1.322355
N	1.219511	1.250433	-1.105379
N	-0.010688	1.58055 1.284311	
N	1.788915	-0.681451	1.022633
N	0.46783 -1.600916	-1.170951	
N	-1.100901	-1.153855	1.214247
S	-1.744672	0.588801	-1.11116

Table S6. Optimized coordinates for $[(en)_2Fe(SOCH_2CH_2NH_2)\text{-}\mathit{N,S}]^{2+}$.

S	-1.742187	-0.358302	-1.022729
N	1.323671	-1.203981	-1.204973
N	0.631864	1.666223	-1.058554
N	-0.182892	-1.580978	1.241067
C	3.187065	-0.260522	0.071013
C	-0.302955	2.688382	0.932989
C	-2.054946	-2.004217	-0.261054
H	3.361835	-1.83774-1.408757	
H	1.456347	-0.727524	-2.111358
H	3.39635 0.530753	-0.657815	
H	2.358026	1.186447	1.304407
H	0.168805	3.753783	-0.907514
H	1.613826	1.9728 -1.017488	
H	0.673279	2.891713	1.386938
H	-1.703394	1.173913	1.033885
H	-1.770807	-2.939284	1.68219
H	0.374748	-2.417531.016362	
H	-1.486351	-2.741961	-0.843194
Fe	0.284617	0.006963	0.063411
O	-2.786679	0.60317 -0.363768	
N	2.123443	0.232739	0.993278
N	-0.693461	1.287005	1.25354
C	2.670735	-1.505167	-0.627338
C	-0.233104	2.789685	-0.580336
C	-1.625663	-1.970196	1.191253
H	2.551421	-2.325197	0.089931
H	0.874081	-2.095135	-1.46024
H	4.114741	-0.471941	0.613676
H	2.145765	-0.329813	1.858362
H	-1.227316	2.654923	-1.013574
H	0.457337	1.51082 -2.064087	
H	-0.569279	1.113145	2.261909
H	-2.202951	-1.219589	1.739042
H	0.065683	-1.371623	2.221821
H	-3.122091	-2.217915	-0.37673
H	-1.026833	3.398535	1.347672

Table S7. Optimized coordinates for $[(en)_2Fe(SOHCH_2CH_2NH_2)\text{-}\mathit{N,S}]^{3+}$.

S	-1.709166	-0.445985	-1.062826
N	1.337016	-1.203558	-1.199138
N	0.662155	1.654908	-1.051747
N	-0.152142	-1.569115	1.252907

C	3.199877	-0.255301	0.04457
C	-0.289315	2.714812	0.910044
C	-2.088863	-2.002663	-0.186928
H	3.369406	-1.834196	-1.437155
H	1.458125	-0.722236	-2.107897
H	3.411476	0.534034	-0.685281
H	2.343998	1.187855	1.260765
H	0.127744	3.72414	-0.970133
H	1.634613	1.992414	-0.997327
H	0.680455	2.991171	1.338944
H	-1.616581	1.185453	1.295862
H	-1.750075	-2.890337	1.753188
H	0.386188	-2.418792	1.019031
H	-1.573365	-2.785658	-0.761914
Fe	0.321789	0.007441	0.071304
O	-2.746606	0.612064	-0.310885
N	2.117742	0.229356	0.954059
N	-0.595518	1.3038	1.307897
C	2.693389	-1.506886	-0.640815
C	-0.244139	2.761311	-0.605687
C	-1.604032	-1.928539	1.24825
H	2.585345	-2.328462	0.076373
H	0.883461	-2.09459-1.457212	
H	4.115984	-0.450171	0.611234
H	2.147519	-0.324868	1.828351
H	-1.234747	2.584849	-1.032578
H	0.513074	1.483784	-2.061878
H	-0.314321.164398	2.292987	
H	-2.152838	-1.165463	1.807854
H	0.125822	-1.366597	2.230059
H	-3.167615	-2.173862	-0.264494
H	-1.0469	3.394513	1.312514
H	-3.560058	0.754265	-0.87734

Table S8. Optimized coordinates for [(en)$_2$Fe(SO$_2$HCH$_2$CH$_2$NH$_2$)-*N,S*]$^{2+}$.

Fe	-0.391233	0.012865	-0.150886
S	1.737222	-0.202243	0.603249
O	1.789324	-0.369175	2.08866
O	2.664966	0.824635	0.022666
N	0.235829	1.437964	-1.428963
N	-0.738819	1.573948	1.092439
N	0.101763	-1.501453	-1.414206
N	-1.120721	-1.307802	1.194515
N	-2.325458	0.065853	-0.82832
C	-0.068798	2.801373	0.555844
C	-0.231215	2.776252	-0.953415
C	2.208241	-1.789522	-0.18254
C	1.570312	-1.77302-1.561324	
C	-2.61548-1.236907	1.204024	
C	-3.081922	-1.076888	-0.232423
H	3.301391	-1.836343	-0.218522
H	1.820047	-2.600166	0.44526
H	1.715591	-2.731119	-2.071339

H	2.002612	-0.984839	-2.185138
H	-0.509103	3.701825	0.995581
H	0.988433	2.758077	0.83075
H	0.342867	3.57559 -1.433423	
H	-1.283044	2.892688	-1.236255
H	-3.039351	-2.133817	1.667126
H	-2.908804	-0.370522	1.806773
H	-4.161419	-0.900614	-0.283795
H	-2.856423	-1.976048	-0.816535
H	-0.839296	-2.283456	1.00742
H	-2.807889	0.943995	-0.583071
H	-0.763025	-1.124028	2.145408
H	-2.388287	0.018059	-1.85781
H	-0.353381	-2.387765	-1.143254
H	-0.284866	-1.299669	-2.352213
H	1.263501	1.456168	-1.486825
H	-0.099165	1.282344	-2.393431
H	-1.745957	1.771742	1.199701
H	-0.407254	1.398324	2.054777

Table S9. Optimized coordinates for NHaseBA, BP86, S=1/2.

Fe	-0.290263	-0.523735	0.170775
S	-0.004477	-1.649513	-1.741937
S	0.172531	1.113885	-1.434405
S	1.799542	-0.7075 0.850506	
O	0.453114	2.691872	-0.858424
O	2.279659	0.474118	1.697033
O	2.844795	-1.0584 -0.249831	
N	-0.937515	-2.048385	1.145457
C	-0.351744	-3.271483	1.363412
O	-0.997807	-4.310194	1.603503
N	-2.163287	-0.210735	-0.204525
C	-3.033574	-0.853619	0.588186
O	-4.248651	-0.571517	0.754536
O	-0.628119	0.632571	1.836849
C	-0.464711	1.805073	2.317175
O	-0.125386	2.832075	1.639961
C	-0.703702	1.966642	3.809662
C	6.963877	1.861161	-2.250876
N	5.578738	1.643492	-2.596133
C	4.815399	0.766542	-1.915491
N	5.286704	0.095894	-0.846266
N	3.540215	0.555244	-2.29939
C	4.437631	2.534969	1.470239
N	3.115837	3.117312	1.355537
C	2.910822	4.406465	1.082889
N	3.927089	5.200874	0.652104
N	1.687249	4.951787	1.272955
N	-4.838855	1.904669	-1.030827
C	-6.261876	1.97454 -1.380868	
C	-6.683008	3.446348	-1.514661
C	-7.102224	1.215205	-0.32329
O	-6.822086	-0.170163	-0.263713

C	-2.563126	1.026347	-0.879533
C	-1.569624	1.305069	-2.012773
C	-3.973125	0.965663	-1.524943
O	-4.217987	0.218203	-2.485402
C	-2.412446	-2.049286	1.32653
C	-2.788507	-2.033309	2.824515
O	-2.222168	-0.932454	3.538288
N	1.726482	-3.799127	0.089006
C	1.197492	-3.380575	1.392082
C	1.912475	-2.144893	1.971964
C	3.540211	-4.402533	-1.441984
C	2.989596	-4.330255	-0.016244
O	3.647009	-4.707672	0.969808
C	-1.401876	-3.22332-3.547768	
C	-1.59887-2.444339	-2.243494	
H	5.253387	1.928969	-3.518539
H	4.593228	-0.505567	-0.353957
H	6.281138	-0.096323	-0.745995
H	2.976371	-0.059206	-1.66774
H	3.047055	1.270726	-2.832569
H	2.328984	2.451448	1.434961
H	3.861826	6.203386	0.821292
H	4.871208	4.822653	0.620985
H	1.423819	5.765745	0.718684
H	0.922212	4.314863	1.563542
H	-4.582479	2.320427	-0.132719
H	1.346794	-3.297912	-0.727956
H	-6.359761.447313	-2.345641	
H	-7.743066	3.523387	-1.815676
H	-6.068728	3.963192	-2.271396
H	-6.567578	3.982132	-0.553125
H	-6.959662	1.71914 0.663535	
H	-8.173591.316401	-0.583589	
H	-5.881896	-0.279220.039283	
H	1.403908	-4.211948	2.088205
H	1.452157	-1.836093	2.925124
H	2.987367	-2.347382	2.116776
H	4.174267	-5.297728	-1.542765
H	4.167969	-3.511266	-1.621673
H	2.746912	-4.42243-2.208231	
H	-2.354359	-3.701989	-3.841246
H	-0.641168	-4.017069	-3.43988
H	-1.088578	-2.55683-4.371313	
H	-2.372821	-1.665667	-2.351888
H	-1.908929	-3.116467	-1.425167
H	-2.525161.869212	-0.15527	
H	-1.726271	0.589253	-2.835543
H	-1.649618	2.332416	-2.404953
H	-2.846554	-2.977480.902664	
H	-3.891715	-1.971124	2.89118
H	-2.463588	-2.995154	3.269488
H	-0.838867	3.02666 4.073001	
H	0.173871	1.571713	4.353602
H	-1.581305	1.375868	4.119065

H	7.339155	2.724969	-2.819366
H	7.607767	0.991256	-2.487401
H	7.059689	2.096469	-1.176786
H	4.299777	1.501895	1.814707
H	5.053913	3.088472	2.203159
H	4.965774	2.500589	0.499431
H	-1.502548	-0.558433	2.970322
H	0.142864	2.726742	0.119964

Table S10. Optimized coordinates for NHaseBA, BP86, S=3/2.

Fe	-0.310196	-0.552522	0.162295
S	0.211951	-1.676371	-1.97206
S	0.131095	1.16332	-1.387858
S	1.837147	-0.634296	1.02323
O	0.302692	2.72675	-0.748211
O	2.247572	0.533277	1.913875
O	2.880512	-0.921543	-0.086014
N	-0.90798	-2.054205	1.188793
C	-0.293844	-3.260965	1.435321
O	-0.919086	-4.303718	1.702107
N	-2.146343	-0.239643	-0.164173
C	-3.025437	-0.925527	0.616952
O	-4.243993	-0.679393	0.727059
O	-0.659231	0.82942	2.009492
C	-0.500972	1.998066	2.472101
O	-0.175773	3.012159	1.753816
C	-0.695516	2.212375	3.966811
C	6.949143	1.844395	-2.318124
N	5.58439	1.557433	-2.706352
C	4.810724	0.732758	-1.975393
N	5.289984	0.139001	-0.863243
N	3.532466	0.498979	-2.332814
C	4.374656	2.588683	1.356134
N	3.046687	3.164843	1.266177
C	2.829522	4.429748	0.901203
N	3.82024	5.16684	0.329783
N	1.633594	5.014448	1.137324
N	-4.892991	1.780002	-1.107822
C	-6.301788	1.822385	-1.517247
C	-6.72311	3.279451	-1.769842
C	-7.175195	1.127655	-0.44349
O	-6.884056	-0.249774	-0.284757
C	-2.599963	0.96897	-0.872761
C	-1.604258	1.263461	-1.997696
C	-3.997192	0.837038	-1.536117
O	-4.204931	0.037976	-2.461884
C	-2.376033	-2.072015	1.397229
C	-2.736568	-1.976389	2.899311
O	-2.365109	-0.744862	3.510034
N	1.799225	-3.706206	0.138289
C	1.25741	-3.339929	1.449253
C	1.952011	-2.116136	2.08451
C	3.593213	-4.436797	-1.372856

C	3.025981	-4.317522	0.043807
O	3.641721	-4.746698	1.036583
C	-1.339624	-3.377377	-3.562348
C	-1.386072	-2.563976	-2.270391
H	5.267291	1.810839	-3.640617
H	4.60558	-0.420618	-0.314818
H	6.284822	-0.052913	-0.765431
H	2.971263	-0.066449	-1.661711
H	3.034045	1.166929	-2.919556
H	2.261332	2.523351	1.459685
H	3.784524	6.180789	0.422283
H	4.751274	4.76596	0.243584
H	1.337521	5.769144	0.518494
H	0.881153	4.410618	1.524841
H	-4.662344	2.26898	-0.240391
H	1.434529	-3.182826	-0.676677
H	-6.359969	1.229523	-2.446327
H	-7.772169	3.327283	-2.112196
H	-6.084532	3.743264	-2.540573
H	-6.64273	3.88352	-0.846109
H	-7.072987	1.694059	0.513571
H	-8.237114	1.196028	-0.747073
H	-5.945313	-0.330969	0.01964
H	1.483531	-4.190865	2.114831
H	1.480617	-1.850041	3.04558
H	3.028184	-2.312984	2.230182
H	3.894528	-5.482213	-1.554717
H	4.500629	-3.811657	-1.443287
H	2.882659	-4.118956	-2.154149
H	-2.30581	-3.893597	-3.720399
H	-0.544579	-4.14367	-3.530714
H	-1.152294	-2.727934	-4.436135
H	-2.199259	-1.817202	-2.306688
H	-1.579515	-3.220696	-1.403897
H	-2.604954	1.817429	-0.156592
H	-1.710746	0.514634	-2.798846
H	-1.730207	2.273815	-2.421049
H	-2.797054	-3.027303	1.02553
H	-3.834632	-2.078778	2.988382
H	-2.268345	-2.843124	3.411174
H	-0.850874	3.276273	4.203444
H	0.210379	1.858992	4.492767
H	-1.54507	1.610168	4.327697
H	7.322658	2.677921	-2.93108
H	7.626798	0.981542	-2.468707
H	6.988669	2.152872	-1.259595
H	4.260421	1.593949	1.806147
H	5.029871	3.206907	1.997614
H	4.84586	2.458537	0.365352
H	-1.59743	-0.359272	3.011055
H	0.011813	2.752534	0.246076

Table S11. Optimized coordinates for NHaseBA, BP86, S=5/2.

Fe	-0.334069	-0.606507	0.125768
S	0.217292	-1.57386-1.994758	
S	0.018206	1.439446	-1.519705
S	2.001233	-0.724225	1.035264
O	0.026963	2.992434	-0.820335
O	2.513181	0.303771	2.046091
O	3.034311	-1.014842	-0.096573
N	-0.947893	-2.187567	1.127307
C	-0.359503	-3.419117	1.286609
O	-0.981044	-4.480632	1.468762
N	-2.241699	-0.235549	-0.17521
C	-3.079244	-0.947674	0.624922
O	-4.293094	-0.718323	0.812949
O	-0.527653	0.632298	1.940359
C	-0.253803	1.761762	2.453377
O	0.147155	2.7723	1.77604
C	-0.391422	1.90632	3.960599
C	6.951737	1.983043	-2.261466
N	5.568918	1.746343	-2.637043
C	4.812463	0.862107	-1.9561
N	5.32489	0.194818	-0.902896
N	3.526002	0.635433	-2.28941
C	4.38804	2.588925	1.445635
N	3.060697	3.173661	1.393281
C	2.844551	4.448368	1.066085
N	3.833952	5.199447	0.509824
N	1.646913	5.025718	1.319882
N	-4.969168	1.831151	-0.983913
C	-6.380268	1.940579	-1.357447
C	-6.716813	3.415681	-1.621666
C	-7.249297	1.304642	-0.245013
O	-6.986263	-0.074927	-0.053922
C	-2.697009	0.980456	-0.835807
C	-1.748234	1.322167	-2.00192
C	-4.111388	0.877743	-1.45734
O	-4.368247	0.086858	-2.377724
C	-2.414792	-2.144639	1.354415
C	-2.731609	-2.089863	2.867201
O	-2.275411	-0.897996	3.502134
N	1.744186	-3.785076	-0.028841
C	1.201585	-3.482581.299044	
C	1.93256	-2.304396	1.990113
C	3.588551	-4.325051	-1.549905
C	2.983027	-4.371497	-0.146992
O	3.580285	-4.883667	0.81608
C	-1.348359	-3.174913	-3.683722
C	-1.364518	-2.489602	-2.320275
H	5.243558	2.053249	-3.552431
H	4.661777	-0.410982	-0.369377
H	6.326474	0.030955	-0.830921
H	3.007129	-0.003474	-1.651875

H	2.994992	1.322582	-2.823116
H	2.255667	2.576115	1.635816
H	3.781412	6.211829	0.613745
H	4.776456	4.817212	0.471305
H	1.349063	5.798797	0.725075
H	0.904957	4.388556	1.664258
H	-4.683767	2.331749	-0.140281
H	1.413175	-3.194034	-0.809357
H	-6.494777	1.337923	-2.274923
H	-7.771962	3.529212	-1.928898
H	-6.076654	3.823699	-2.421937
H	-6.5646 4.027358	-0.712077	
H	-7.109114	1.895459	0.692508
H	-8.316237	1.390284	-0.524808
H	-6.033417	-0.176993	0.195606
H	1.4299 -4.367584	1.918607	
H	1.447816	-2.045192	2.946511
H	2.986515	-2.583171	2.168122
H	4.091884	-5.282019	-1.763315
H	4.350156	-3.525363	-1.573773
H	2.842405	-4.114098	-2.334444
H	-2.304254	-3.707507	-3.847316
H	-0.529362	-3.912408	-3.757818
H	-1.222403	-2.441389	-4.499901
H	-2.200849	-1.771665	-2.247729
H	-1.4958 -3.231124	-1.512884	
H	-2.671674	1.818432	-0.106286
H	-1.815925	0.545654	-2.781393
H	-2.008138	2.295003	-2.453106
H	-2.871288	-3.075739	0.961443
H	-3.828463	-2.139565	2.993626
H	-2.288983	-2.995915	3.333324
H	-0.454892.964585	4.256906	
H	0.49999 1.456347	4.434775	
H	-1.274553	1.352939	4.318714
H	7.325306	2.848766	-2.827866
H	7.608334	1.119721	-2.485535
H	7.019769	2.221213	-1.186428
H	4.265544	1.557802	1.804517
H	5.040817	3.149014	2.141233
H	4.861654	2.5496 0.4478	
H	-1.491852	-0.561892.996702	
H	-0.004276	2.851861	0.190565

Table S12. Optimized coordinates for NHaseBA, BP86+10%HF, S=1/2.

Fe	-0.310142	-0.511791	0.167365
S	-0.018273	-1.644096	-1.740608
S	0.160655	1.137496	-1.436034
S	1.803155	-0.701266	0.849611
O	0.421288	2.695231	-0.854425
O	2.278786	0.474314	1.68566
O	2.830626	-1.053472	-0.244554
N	-0.955461	-2.032641	1.139517

C	-0.373242	-3.250089	1.344086
O	-1.012226	-4.289401	1.567253
N	-2.172735	-0.19516-0.205068	
C	-3.041535	-0.839410.579112	
O	-4.250033	-0.558745	0.743029
O	-0.620822	0.62488	1.829165
C	-0.398869	1.774567	2.323746
O	-0.051438	2.796068	1.656544
C	-0.574968	1.912591	3.821369
C	6.962763	1.811883	-2.274918
N	5.577369	1.593738	-2.606318
C	4.811167	0.729057	-1.922191
N	5.276822	0.06394	-0.855674
N	3.540894	0.526357	-2.304873
C	4.461889	2.51378	1.458075
N	3.14596	3.105276	1.344177
C	2.941737	4.390966	1.071638
N	3.957739	5.179685	0.653177
N	1.721485	4.930068	1.250221
N	-4.832427	1.921146	-1.004574
C	-6.248942	1.99796	-1.356891
C	-6.671742	3.465904	-1.465168
C	-7.091976	1.224203	-0.322045
O	-6.807323	-0.15402-0.280139	
C	-2.562943	1.040387	-0.874201
C	-1.572041.306042		-2.008285
C	-3.969615	0.997639	-1.515466
O	-4.214538	0.272725	-2.484912
C	-2.424478	-2.034956	1.309231
C	-2.806982	-2.023742.798867	
O	-2.270562	-0.908313	3.50042
N	1.706723	-3.779454	0.089191
C	1.172554	-3.357634	1.381876
C	1.888788	-2.128208	1.963143
C	3.514025	-4.431732	-1.418871
C	2.960271	-4.317181	-0.003176
O	3.610677	-4.676435	0.987146
C	-1.434728	-3.237767	-3.500558
C	-1.593567	-2.486626	-2.180199
H	5.246177	1.883497	-3.521364
H	4.588415	-0.526209	-0.354225
H	6.269915	-0.088842	-0.721753
H	2.968505	-0.079936	-1.683759
H	3.064598	1.216193	-2.877762
H	2.355927	2.45177	1.437252
H	3.885348	6.184002	0.77909
H	4.897301	4.802552	0.60485
H	1.465147	5.761747	0.727377
H	0.960258	4.301633	1.551007
H	-4.575102	2.325322	-0.106028
H	1.327925	-3.293255	-0.73166
H	-6.344057	1.491065	-2.328533
H	-7.729435	3.546151	-1.760831
H	-6.062233	3.994358	-2.212831

H	-6.554429	3.984732	-0.498527
H	-6.960061	1.710877	0.670209
H	-8.157841	1.325803	-0.588694
H	-5.874588	-0.269676	0.026518
H	1.369412	-4.183267	2.081055
H	1.423171	-1.815271	2.908349
H	2.956854	-2.339899	2.11981
H	4.039553	-5.390607	-1.524942
H	4.245778	-3.623887	-1.573547
H	2.738169	-4.349198	-2.193175
H	-2.378716	-3.746629	-3.754564
H	-0.642556	-4.000639	-3.442029
H	-1.187166	-2.549963	-4.324269
H	-2.394006	-1.736359	-2.242939
H	-1.842551	-3.179335	-1.362728
H	-2.514349	1.88133	-0.153746
H	-1.724719	0.575861	-2.813731
H	-1.661047	2.321935	-2.418434
H	-2.856868	-2.954987	0.875656
H	-3.906613	-1.983329	2.866326
H	-2.462854	-2.972398	3.248537
H	-0.642064	2.967872	4.113003
H	0.296802	1.4579	4.318463
H	-1.468669	1.364347	4.149076
H	7.332751	2.66613	-2.854028
H	7.599641	0.940667	-2.506591
H	7.068234	2.059352	-1.208113
H	4.314585	1.480438	1.785661
H	5.075662	3.050682	2.199791
H	4.992163	2.492083	0.49215
H	-1.52345	-0.560541	2.961757
H	0.150711	2.719176	0.128892

Table S13. Optimized coordinates for NHaseBA, BP86+10%HF, S=3/2.

Fe	-0.334112	-0.536209	0.171343
S	0.18924	-1.658322	-1.971751
S	0.11915	1.196545	-1.384346
S	1.84113	-0.651055	1.002825
O	0.265162	2.742373	-0.741638
O	2.264746	0.503749	1.884796
O	2.865016	-0.940246	-0.104621
N	-0.933508	-2.048162	1.172409
C	-0.32552	-3.253901	1.396421
O	-0.949077	-4.295126	1.639825
N	-2.162848	-0.220696	-0.164144
C	-3.038943	-0.90652	0.607867
O	-4.248789	-0.654096	0.726443
O	-0.626115	0.789631	1.98076
C	-0.405585	1.929112	2.476111
O	-0.050212	2.9424	1.787437
C	-0.557707	2.099126	3.974562
C	6.948076	1.810325	-2.328733
N	5.580596	1.531088	-2.70172

C	4.804824	0.71112	-1.975646
N	5.276246	0.114175	-0.870111
N	3.531825	0.489137	-2.335067
C	4.399103	2.563913	1.361441
N	3.078592	3.155505	1.288722
C	2.86262	4.41506	0.918412
N	3.850756	5.14332	0.349005
N	1.671454	4.997063	1.149574
N	-4.883424	1.819315	-1.074731
C	-6.288821	1.871198	-1.47639
C	-6.711899	3.327385	-1.699937
C	-7.158405	1.163389	-0.417521
O	-6.869029	-0.209501	-0.279962
C	-2.600367	0.991821	-0.860516
C	-1.607621	1.274967	-1.986467
C	-3.997009	0.882581	-1.516757
O	-4.212076	0.098523	-2.445129
C	-2.397152	-2.065253	1.36755
C	-2.758993	-1.989204	2.86243
O	-2.356453	-0.777765	3.482063
N	1.764036	-3.706013	0.114331
C	1.221235	-3.337511	1.417719
C	1.923848	-2.124794	2.054086
C	3.559423	-4.451998	-1.374889
C	2.987496	-4.310878	0.031181
O	3.598486	-4.721931	1.027272
C	-1.379282	-3.358892	-3.534375
C	-1.405821	-2.543349	-2.248058
H	5.260139	1.788832	-3.629836
H	4.594883	-0.442187	-0.323653
H	6.269813	-0.045569	-0.747051
H	2.962485	-0.082732	-1.685545
H	3.050611	1.131133	-2.956992
H	2.288017	2.533751	1.500849
H	3.804265	6.15622	0.391344
H	4.775158	4.74186	0.242315
H	1.379063	5.761245	0.547721
H	0.926763	4.400114	1.546487
H	-4.645241	2.300675	-0.209806
H	1.401384	-3.190878	-0.701645
H	-6.35237	1.297883	-2.412838
H	-7.761233	3.380634	-2.028946
H	-6.083387	3.802834	-2.467059
H	-6.621485	3.913836	-0.7699
H	-7.055782	1.711779	0.545187
H	-8.216768	1.238212	-0.71844
H	-5.936163	-0.303226	0.023321
H	1.434673	-4.185879	2.083873
H	1.450133	-1.852192	3.008133
H	2.992013	-2.337957	2.209966
H	3.832935	-5.502972	-1.547316
H	4.480429	-3.853478	-1.440332
H	2.864193	-4.121693	-2.159635
H	-2.344583	-3.873451	-3.67745

H	-0.586188	-4.122656	-3.511169
H	-1.203396	-2.713968	-4.409567
H	-2.217637	-1.800767	-2.27703
H	-1.589363	-3.196906	-1.38135
H	-2.589883	1.837103	-0.14599
H	-1.709970.517306	-2.774398	
H	-1.740667	2.275132	-2.422692
H	-2.820618	-3.006837	0.975669
H	-3.854987	-2.065231	2.951697
H	-2.311069	-2.867593.363199	
H	-0.683258	3.155136	4.244987
H	0.354182	1.715583	4.459918
H	-1.405659	1.504965	4.340758
H	7.319134	2.634426	-2.949231
H	7.615182	0.944312	-2.479197
H	7.000677	2.126709	-1.276703
H	4.274408	1.564489	1.788749
H	5.061505	3.159439	2.010532
H	4.861113	2.451255	0.368329
H	-1.583827	-0.410927	2.989632
H	0.044668	2.745595	0.261736

Table S14. Optimized coordinates for NHaseBA, BP86+10%HF, S=5/2.

Fe	-0.355496	-0.589213	0.12718
S	0.194666	-1.566599	-1.987434
S	-0.010142	1.45975	-1.521118
S	1.993063	-0.720194	1.011849
O	-0.016826	2.992401	-0.820148
O	2.487381	0.324221	1.99927
O	3.02331	-1.012897	-0.101864
N	-0.952685	-2.176526	1.118509
C	-0.357261	-3.395761	1.286281
O	-0.968647	-4.453658	1.476916
N	-2.257094	-0.232972	-0.178639
C	-3.087076	-0.951067	0.613558
O	-4.295374	-0.730283	0.801529
O	-0.503857	0.62487	1.896396
C	-0.197011.733199	2.426377	
O	0.152744	2.759786	1.762459
C	-0.231744	1.819416	3.937087
C	6.949681	1.944065	-2.283478
N	5.566675	1.70578	-2.645462
C	4.807949	0.832317	-1.96191
N	5.316661	0.166247	-0.914677
N	3.52474	0.618522	-2.290893
C	4.415074	2.56387	1.441276
N	3.093396	3.155301	1.386195
C	2.876699	4.429393	1.071096
N	3.869453	5.186057	0.546868
N	1.676907	4.99292	1.305784
N	-4.970116	1.838119	-0.948418
C	-6.376041.958429	-1.314847	
C	-6.707858	3.43437	-1.547361

C	-7.242391.307054	-0.218708	
O	-6.977596	-0.068241	-0.050026
C	-2.707890.983762	-0.828953	
C	-1.767355	1.322996	-1.99619
C	-4.121101	0.894546	-1.43847
O	-4.385194	0.119298	-2.361643
C	-2.416274	-2.142995	1.335252
C	-2.733122	-2.084578	2.841223
O	-2.274732	-0.890524	3.459036
N	1.738834	-3.760468	-0.025424
C	1.199249	-3.453033	1.296445
C	1.926062	-2.272949	1.979019
C	3.565333	-4.349304	-1.542256
C	2.973632	-4.340393	-0.138411
O	3.577873	-4.821328	0.828943
C	-1.381847	-3.180385	-3.641794
C	-1.373332	-2.501926	-2.279564
H	5.235153	2.018286	-3.552828
H	4.65699	-0.429546	-0.375669
H	6.316381	0.027584	-0.822322
H	2.999829	-0.019624	-1.666482
H	3.007525	1.285316	-2.855309
H	2.293429	2.553795	1.616336
H	3.80455	6.19605	0.625763
H	4.808757	4.807697	0.49745
H	1.392801	5.790827	0.745278
H	0.934478	4.361614	1.644615
H	-4.676931	2.33241	-0.108588
H	1.397847	-3.189127	-0.810208
H	-6.495336	1.377648	-2.241276
H	-7.761552	3.558593	-1.842806
H	-6.073919	3.85375	-2.342083
H	-6.546049	4.025665	-0.630173
H	-7.104177	1.879227	0.725695
H	-8.305561	1.3964	-0.496938
H	-6.029996	-0.180011	0.196078
H	1.426395	-4.329771.920514	
H	1.438295	-2.006823	2.927919
H	2.975356	-2.549704	2.165461
H	3.927352	-5.360787	-1.775271
H	4.431895	-3.671257	-1.559012
H	2.851843	-4.027667	-2.314138
H	-2.330007	-3.725668	-3.782428
H	-0.555789	-3.90205-3.739498	
H	-1.288577	-2.443999	-4.455112
H	-2.214062	-1.79804-2.187418	
H	-1.477595	-3.246669	-1.476047
H	-2.672643	1.816665	-0.099671
H	-1.828258	0.541116	-2.765511
H	-2.036437	2.285799	-2.454563
H	-2.86769-3.069710.938138		
H	-3.825589	-2.129421	2.970811
H	-2.290885	-2.981859	3.314054
H	-0.260527	2.86228	4.276379

H	0.683423	1.341958	4.322226
H	-1.091761	1.262412	4.332974
H	7.31755	2.801187	-2.859419
H	7.600204	1.080333	-2.503677
H	7.027004	2.193203	-1.215132
H	4.284038	1.533959	1.788569
H	5.064569	3.111355	2.143959
H	4.892397	2.532742	0.448906
H	-1.479992	-0.578608	2.968209
H	-0.033545	2.858497	0.184932

Table S15. Optimized coordinates for NHaseBA, B3LYP, S=1/2.

Fe	-0.322731	-0.510566	0.169437
S	-0.006852	-1.622255	-1.779556
S	0.151133	1.229581	-1.420613
S	1.850095	-0.727270.878138	
O	0.378511	2.769152	-0.795937
O	2.324564	0.418431	1.743186
O	2.86718	-1.05812-0.220357	
N	-0.965945	-2.062474	1.122088
C	-0.386023	-3.278471.30094	
O	-1.018733	-4.320713	1.500799
N	-2.187112	-0.186937	-0.196858
C	-3.051419	-0.852078	0.570494
O	-4.256894	-0.583516	0.731174
O	-0.610941	0.598073	1.870882
C	-0.359622	1.725423	2.391409
O	0.005757	2.750808	1.751996
C	-0.519114	1.827602	3.894401
C	6.969131	1.839942	-2.271057
N	5.577965	1.63381	-2.595399
C	4.817329	0.753533	-1.93122
N	5.293107	0.062899	-0.891232
N	3.548351	0.55604	-2.309549
C	4.456022	2.519427	1.457815
N	3.141088	3.121217	1.373319
C	2.935767	4.397627	1.076955
N	3.940213	5.172051	0.616813
N	1.724366	4.944717	1.270886
N	-4.849732	1.927703	-0.996084
C	-6.262665	2.009517	-1.36088
C	-6.669044	3.479457	-1.495141
C	-7.120145	1.257724	-0.324253
O	-6.85049-0.12292-0.266777		
C	-2.581906	1.055417	-0.848617
C	-1.589934	1.362737	-1.973873
C	-3.981376	1.012154	-1.501632
O	-4.216349	0.290484	-2.469019
C	-2.435875	-2.059856	1.283438
C	-2.832042	-2.076687	2.769127
O	-2.326826	-0.961218	3.492142
N	1.704398	-3.807169	0.052466
C	1.162271	-3.395623	1.346452

C	1.890643	-2.185105	1.954172
C	3.522431	-4.410841	-1.460271
C	2.958783	-4.334945	-0.046197
O	3.607426	-4.705226	0.93456
C	-1.420655	-3.20908-3.550933	
C	-1.56886-2.496956	-2.209858	
H	5.241236	1.952104	-3.493526
H	4.623375	-0.528337	-0.38148
H	6.283299	-0.032175	-0.726263
H	2.98105	-0.062102	-1.713666
H	3.07515	1.231679	-2.893164
H	2.348442	2.494831	1.523691
H	3.863483	6.176261	0.697046
H	4.876215	4.800402	0.5609
H	1.462945	5.770136	0.749474
H	0.971808	4.332491	1.597034
H	-4.598766	2.339387	-0.105487
H	1.328051	-3.325452	-0.762447
H	-6.356474	1.495499	-2.321224
H	-7.719263	3.567031	-1.79532
H	-6.052949	3.984317	-2.245738
H	-6.549199	4.011164	-0.542232
H	-6.989654	1.744715	0.660086
H	-8.177441	1.364562	-0.59688
H	-5.928083	-0.255087	0.039542
H	1.341956	-4.226195	2.033263
H	1.422554	-1.885021	2.895332
H	2.946683	-2.416631	2.120664
H	4.175304	-5.283023	-1.540709
H	4.121414	-3.511247	-1.642915
H	2.741868	-4.461732	-2.225011
H	-2.352062	-3.733109	-3.797976
H	-0.612644	-3.949125	-3.529031
H	-1.208836	-2.498507	-4.35779
H	-2.382858	-1.769881	-2.241965
H	-1.785851	-3.214928	-1.41395
H	-2.551631	1.878795	-0.117566
H	-1.7213	0.650316	-2.789234
H	-1.706966	2.378024	-2.362175
H	-2.868011	-2.961983	0.830248
H	-3.925425	-2.056064	2.832871
H	-2.475391	-3.017588	3.208886
H	-0.519814	2.870326	4.21696
H	0.319925	1.305706	4.369279
H	-1.439807	1.329701	4.209165
H	7.341565	2.680874	-2.857563
H	7.589202	0.963623	-2.500168
H	7.08418	2.093405	-1.212553
H	4.312951	1.492016	1.784312
H	5.085405	3.050576	2.181877
H	4.96033	2.502979	0.484856
H	-1.557451	-0.615588	2.999553
H	0.147772	2.756276	0.184151

Table S16. Optimized coordinates for NHaseBA, B3LYP, S=3/2.

Fe	-0.344719	-0.528015	0.188677
S	0.18129	-1.61576	-2.006786
S	0.110054	1.30546	-1.349651
S	1.875147	-0.698906	1.012222
O	0.220549	2.828555	-0.658867
O	2.315936	0.417542	1.92396
O	2.883036	-0.963465	-0.105376
N	-0.948473	-2.081199	1.150581
C	-0.347915	-3.291501	1.340021
O	-0.972069	-4.331945	1.555844
N	-2.17917	-0.206951	-0.153285
C	-3.04969	-0.909184	0.602493
O	-4.254578	-0.660865	0.726847
O	-0.59684	0.752623	2.004222
C	-0.329041	1.853956	2.552514
O	0.05655	2.881678	1.91714
C	-0.457563	1.946304	4.059705
C	6.949908	1.843845	-2.326482
N	5.572527	1.589456	-2.684588
C	4.806244	0.741145	-1.987391
N	5.287121	0.095511	-0.91957
N	3.532633	0.534573	-2.343033
C	4.395182	2.561525	1.366853
N	3.080829	3.173906	1.347101
C	2.859286	4.416792	0.939408
N	3.822449	5.115593	0.303476
N	1.684968	5.016087	1.199292
N	-4.896408	1.841848	-1.055702
C	-6.298024	1.896921	-1.47064
C	-6.711103	3.354026	-1.704216
C	-7.180795	1.195838	-0.420482
O	-6.900405	-0.178361	-0.280599
C	-2.616191	1.019517	-0.822773
C	-1.622343	1.354157	-1.93552
C	-4.003892	0.915304	-1.495365
O	-4.206805	0.140049	-2.426686
C	-2.414317	-2.089516	1.33665
C	-2.785397	-2.051696	2.829716
O	-2.369686	-0.862711	3.484238
N	1.745014	-3.751389	0.057339
C	1.199974	-3.393176	1.364184
C	1.921472	-2.203828	2.022159
C	3.560234	-4.427606	-1.438611
C	2.97388	-4.337269	-0.035186
O	3.587997	-4.755613	0.949338
C	-1.375196	-3.313893	-3.595066
C	-1.419719	-2.488312	-2.31659
H	5.242099	1.886173	-3.592272
H	4.62152	-0.465432	-0.37252
H	6.278784	-0.01341	-0.772318
H	2.967192	-0.062401	-1.728831
H	3.05439	1.177471	-2.958549

H	2.291325	2.592076	1.628667
H	3.763971	6.123596	0.271724
H	4.739474	4.716051	0.175477
H	1.378382	5.766556	0.595118
H	0.955841	4.440904	1.630944
H	-4.667525	2.320404	-0.193122
H	1.384462	-3.238948	-0.750721
H	-6.357695	1.327536	-2.401845
H	-7.752768	3.412228	-2.03871
H	-6.077518	3.817645	-2.466666
H	-6.622306	3.943126	-0.782259
H	-7.084208	1.735267	0.539794
H	-8.230169	1.27409	-0.729188
H	-5.979268	-0.286617	0.031155
H	1.393056	-4.248061	2.016354
H	1.454481	-1.944779	2.976376
H	2.978596	-2.439242.17702	
H	4.037196	-5.403186	-1.563883
H	4.332891	-3.657366	-1.542529
H	2.814374	-4.277138	-2.223983
H	-2.337337	-3.818869	-3.752352
H	-0.593737	-4.080904	-3.55021
H	-1.177482	-2.682312	-4.468556
H	-2.22148-1.746568		-2.368392
H	-1.62661-3.135043		-1.458313
H	-2.624177	1.840992	-0.091282
H	-1.702480.624515		-2.742244
H	-1.781941	2.357325	-2.339954
H	-2.842287	-3.009112	0.918296
H	-3.87594-2.112209		2.913887
H	-2.353448	-2.940419	3.310218
H	-0.523642.985785		4.387266
H	0.433915	1.490888	4.507403
H	-1.327888	1.381332	4.402528
H	7.316942	2.675989	-2.928638
H	7.598764	0.978184	-2.511143
H	7.023668	2.129744	-1.272805
H	4.277791	1.57054	1.799643
H	5.090241	3.149142	1.978313
H	4.806747	2.444972	0.358848
H	-1.587663	-0.501373.020794	
H	0.060409	2.784504	0.33924

Table S17. Optimized coordinates for NHaseBA, B3LYP, S=5/2.

Fe	-0.349816	-0.584348	0.141327
S	0.191814	-1.518187	-2.017511
S	-0.031136	1.57909	-1.479232
S	2.038063	-0.780611.001759	
O	-0.068064	3.078761	-0.719306
O	2.546938	0.238593	1.99981
O	3.057429	-1.058686	-0.116003
N	-0.947044	-2.210431	1.085446
C	-0.35608-3.429894		1.234545

O	-0.969194	-4.483239	1.408666
N	-2.254607	-0.215906	-0.160945
C	-3.079122	-0.958358	0.60712
O	-4.283376	-0.746693	0.797728
O	-0.447562	0.577264	1.922626
C	-0.177281	1.673767	2.489152
O	0.153017	2.724822	1.869041
C	-0.236405	1.706412	4.001646
C	6.947211	2.008632	-2.265419
N	5.558498	1.781271	-2.616331
C	4.810743	0.882178	-1.960783
N	5.325588	0.181657	-0.945323
N	3.530431	0.673161	-2.291605
C	4.39385	2.586211	1.45334
N	3.070661	3.176342	1.404621
C	2.846177	4.445461	1.090562
N	3.819662	5.20351	0.544542
N	1.651955	5.004477	1.347993
N	-4.989705	1.832445	-0.936231
C	-6.392111	1.942332	-1.318163
C	-6.721673.414185	-1.574656	
C	-7.268519	1.303665	-0.224823
O	-7.005637	-0.069768	-0.040364
C	-2.722491.006976	-0.788256	
C	-1.788779	1.408154	-1.942521
C	-4.126763	0.899481	-1.411184
O	-4.372968	0.118676	-2.327732
C	-2.413548	-2.173511.295849	
C	-2.741083	-2.162466	2.799793
O	-2.26829-0.996328	3.459919	
N	1.741671	-3.81019-0.074537	
C	1.200635	-3.506551	1.249442
C	1.944503	-2.345442	1.946529
C	3.596679	-4.309563	-1.58451
C	2.986964	-4.361348	-0.191175
O	3.593313	-4.845161	0.766372
C	-1.348643	-3.15391-3.69576	
C	-1.391568	-2.419177	-2.363383
H	5.219944	2.121093	-3.50592
H	4.682918	-0.427175	-0.414854
H	6.322285	0.096719	-0.819735
H	3.009158	0.006748	-1.710883
H	3.017626	1.328386	-2.864618
H	2.277647	2.590663	1.665118
H	3.740572	6.21005	0.583013
H	4.75258	4.833088	0.445721
H	1.3564	5.804496	0.805508
H	0.922363	4.387095	1.712203
H	-4.710397	2.336893	-0.10445
H	1.399928	-3.245223	-0.852547
H	-6.503482	1.355413	-2.233672
H	-7.768398	3.535323	-1.876076
H	-6.085905	3.817124	-2.368971
H	-6.563253	4.017198	-0.671182

H	-7.139641	1.876467	0.712225
H	-8.323309	1.38781	-0.512031
H	-6.068017	-0.188334	0.21272
H	1.406234	-4.386169	1.864018
H	1.460436	-2.084766	2.891874
H	2.98159	-2.640047	2.135161
H	4.214777	-5.196858	-1.740655
H	4.239581	-3.424061	-1.644117
H	2.843687	-4.237925	-2.374746
H	-2.300526	-3.673274	-3.86448
H	-0.546523	-3.900089	-3.718068
H	-1.189211	-2.460114	-4.528759
H	-2.212402	-1.697081	-2.348544
H	-1.56045-3.126538	-1.546326	
H	-2.717755	1.817776	-0.043931
H	-1.825605	0.656837	-2.733674
H	-2.088152.371346	-2.365375	
H	-2.86861-3.075572	0.867411	
H	-3.827975	-2.190312.92298	
H	-2.317711	-3.072343	3.247984
H	-0.252392.732782	4.372946	
H	0.655581	1.199288	4.387812
H	-1.112294	1.158416	4.358535
H	7.31028	2.86584	-2.833793
H	7.585445	1.147697	-2.502032
H	7.03829	2.244613	-1.200996
H	4.267643	1.555408	1.779031
H	5.036653	3.122093	2.162028
H	4.870246	2.578812	0.466703
H	-1.453194	-0.700759	3.011085
H	-0.072127	2.915832	0.268092

Table S18. Optimized coordinates for NHaseAq, BP86, S=1/2.

Fe	0.275289	-0.518667	-0.279562
S	-0.025427	-1.337536	1.770538
S	-0.165782	1.581358	0.655631
S	-1.797473	-0.774655	-0.875237
O	-0.144644	2.756676	-0.473626
O	-2.206156	0.348804	-1.86846
O	-2.868269	-0.933775	0.232684
N	0.935931	-2.160533	-1.046611
C	0.342347	-3.384612	-1.17931
O	0.971065	-4.451665	-1.339775
N	2.141975	-0.120610.008882	
C	3.036559	-0.917541	-0.598488
O	4.272285	-0.700495	-0.707031
O	0.456936	0.346747	-2.193137
O	1.482889	2.731956	-2.495679
C	-6.979297	2.095942	2.078619
N	-5.583727	1.901691	2.451572
C	-4.784084	0.990671	1.855753
N	-5.2664	0.171504	0.901681
N	-3.484625	0.887885	2.204148

C	-4.558496	2.507429	-1.58325
N	-3.222965	3.053156	-1.402653
C	-2.989479	4.364819	-1.243095
N	-4.006021	5.250269	-1.46682
N	-1.809332	4.782812	-0.760364
N	4.851996	1.996603	0.791408
C	6.23128	2.089248	1.276508
C	6.675582	3.560766	1.257063
C	7.139385	1.175588	0.415241
O	6.789841	-0.193951	0.487132
C	2.575755	1.183996	0.515345
C	1.491254	1.745201	1.439193
C	3.898933	1.165668	1.32072
O	4.008726	0.571217	2.4045
C	2.412217	-2.177501	-1.216855
C	2.80272	-2.2769	-2.711929
O	2.40653	-1.143924	-3.48129
N	-1.744711	-3.774621	0.140395
C	-1.210273	-3.479895	-1.193203
C	-1.947718	-2.303682	-1.863379
C	-3.595709	-4.240273	1.672289
C	-3.006096	-4.30463	0.267241
O	-3.636319	-4.777055	-0.695915
C	1.342336	-2.88888	3.674486
C	1.560501	-2.095637	2.375616
H	2.303798	-3.321858	4.006574
H	0.625312	-3.71644	3.528561
H	0.961541	-2.242494	4.485788
H	2.307197	-1.294275	2.510402
H	1.925664	-2.763739	1.577373
H	-3.796906	6.244698	-1.394805
H	-4.705915	5.012657	-2.169079
H	-1.514683	5.745441	-0.917576
H	-1.083806	4.037067	-0.519058
H	-2.440708	2.379619	-1.36649
H	-4.483562	1.419072	-1.463602
H	-4.96038	2.711135	-2.595651
H	-5.255371	2.918981	-0.83233
H	-7.333516	3.03111	2.536447
H	-7.629737	1.271289	2.428359
H	-7.070258	2.191988	0.983217
H	-5.242254	2.33986	3.305551
H	-4.575105	-0.447065	0.428739
H	-6.258847	-0.049219	0.858029
H	-2.919842	0.223827	1.634023
H	-3.001746	1.692899	2.601296
H	-4.237382	-5.119785	1.840648
H	-4.224234	-3.334697	1.749475
H	-2.825234	-4.188466	2.460258
H	-1.368952	-3.207309	0.9149
H	-1.5206	-2.077846	-2.854837
H	-3.024544	-2.525417	-1.957868
H	-1.426679	-4.368344	-1.811093
H	2.84128	-3.067659	-0.714293

H	3.904413	-2.342678	-2.772027
H	2.374342	-3.218998	-3.113274
H	6.215785	1.694121	2.306923
H	7.702688	3.665778	1.650302
H	6.002589	4.180415	1.873565
H	6.668834	3.963849	0.226413
H	7.127934	1.561119	-0.633366
H	8.181616	1.261816	0.777808
H	2.696685	1.881544	-0.340974
H	1.46062	1.17101	2.380141
H	1.644613	2.813972	1.667101
H	5.878197	-0.303026	0.107198
H	4.713251	2.268215	-0.184257
H	1.528401	-0.845423	-3.13134
H	2.421761	2.856871	-2.249554
H	0.943976	2.880921	-1.638244
H	-0.473999	0.600092	-2.446798
H	1.030947	1.18201	-2.336926

Table S19. Optimized coordinates for NHaseAq, BP86, S=3/2.

Fe	0.310864	-0.521065	-0.243443
S	-0.209353	-1.312952.00367	
S	-0.113961	1.645418	0.536658
S	-1.855268	-0.751478	-0.969973
O	-0.066643	2.744758	-0.662232
O	-2.274545	0.332015	-1.984906
O	-2.889109	-0.896466	0.164976
N	0.937896	-2.165837	-1.025076
C	0.327887	-3.3859	-1.150705
O	0.947312	-4.458743	-1.294487
N	2.138654	-0.092323	-0.060422
C	3.040149	-0.933411	-0.639484
O	4.268334	-0.718683	-0.723085
O	0.382231	0.307963	-2.626782
O	1.502291	2.726848	-2.755488
C	-6.974482	2.134318	2.079808
N	-5.585961.923655		2.478726
C	-4.785489	1.011335	1.884956
N	-5.268834	0.211418	0.9151
N	-3.490060.890177		2.23952
C	-4.486145	2.558231	-1.53501
N	-3.144192	3.097617	-1.37871
C	-2.906764.398385		-1.149566
N	-3.943364	5.285028	-1.249168
N	-1.700495	4.801343	-0.725577
N	4.902853	1.959395	0.839308
C	6.25242	2.018099	1.409658
C	6.705198	3.483692	1.511778
C	7.206658	1.151637	0.551205
O	6.844723	-0.218128	0.518145
C	2.61864	1.205924	0.440731
C	1.533651	1.809484	1.333796
C	3.913259	1.125888	1.289427

O	3.968406	0.476909	2.344588
C	2.40703 -2.198305	-1.222512	
C	2.785817	-2.327964	-2.72225
O	2.469234	-1.181761	-3.500191
N	-1.77909-3.714898	0.163455	
C	-1.224978	-3.466807	-1.17013
C	-1.95145-2.31741-1.901687		
C	-3.640407	-4.228063	1.672353
C	-3.023094	-4.285837	0.279138
O	-3.618773	-4.800172	-0.685345
C	1.273381	-2.940116	3.773642
C	1.390118	-2.136693	2.47292
H	2.244772	-3.414243	4.011121
H	0.514166	-3.737768	3.686018
H	0.992276	-2.292 4.623176	
H	2.169315	-1.358082	2.554515
H	1.675104	-2.803597	1.640402
H	-3.727979	6.2764 -1.153443	
H	-4.687258	5.07777 -1.915313	
H	-1.436841	5.780707	-0.820894
H	-0.949409	4.049858	-0.595096
H	-2.355312.434283	-1.4118	
H	-4.396438	1.464331	-1.532825
H	-4.946209	2.860594	-2.496619
H	-5.140549	2.883016	-0.7072
H	-7.328754	3.067165	2.542186
H	-7.637791	1.311369	2.408398
H	-7.042885	2.242708	0.984173
H	-5.256682	2.351518	3.342758
H	-4.585563	-0.400870.425976	
H	-6.263491	0.004803	0.858786
H	-2.924025	0.227945	1.675311
H	-3.001851	1.673677	2.670621
H	-4.074447	-5.210701	1.921158
H	-4.463276	-3.491235	1.662379
H	-2.920481	-3.934537	2.454642
H	-1.42846-3.117556	0.931555	
H	-1.491106	-2.115843	-2.883925
H	-3.021413	-2.558252	-2.025939
H	-1.442001	-4.37532-1.758104	
H	2.839334	-3.076385	-0.702704
H	3.881254	-2.464201	-2.781829
H	2.302646	-3.248809	-3.110999
H	6.173089	1.560621	2.410749
H	7.710017	3.550798	1.965723
H	6.004149	4.066597	2.132743
H	6.755925	3.95558 0.512028	
H	7.263616	1.598219	-0.471014
H	8.224277	1.201625	0.982367
H	2.791637	1.880027	-0.424308
H	1.474704	1.250784	2.283665
H	1.705128	2.878938	1.544798
H	5.94419 -0.291418	0.113343	
H	4.804863	2.312615	-0.114745

H	1.551129	-0.881639	-3.257663
H	2.447116	2.621963	-2.522369
H	1.011365	2.819518	-1.868114
H	-0.572134	0.499363	-2.801011
H	0.881157	1.177258	-2.768908

Table S20. Optimized coordinates for NHaseAq, BP86, S=5/2.

Fe	0.317052	-0.589087	-0.216631
S	-0.242723	-1.280515	1.996327
S	-0.027177	1.87129	0.500479
S	-2.060875	-0.847995	-0.927078
O	0.093753	2.95677	-0.72231
O	-2.517222	0.169025	-2.002605
O	-3.127862	-1.074827	0.170296
N	0.980154	-2.276805	-0.997543
C	0.388286	-3.509286	-1.070869
O	1.001119	-4.588098	-1.176077
N	2.217719	-0.09242-0.068933	
C	3.088609	-0.9578	-0.655555
O	4.320241	-0.778177	-0.783823
O	0.205578	0.201301	-2.399449
O	1.463211	2.453045	-2.856151
C	-6.976321	2.253485	2.034441
N	-5.565648	2.090411	2.398029
C	-4.793279	1.11595	1.862246
N	-5.319039	0.269441	0.959372
N	-3.497037	0.970606	2.207691
C	-4.467263	2.626377	-1.57161
N	-3.121073.160828	-1.434553	
C	-2.879015	4.461026	-1.196512
N	-3.931614	5.33662	-1.243027
N	-1.661301	4.874864	-0.832074
N	4.976677	1.954202	0.797158
C	6.315147	2.040179	1.382954
C	6.713668	3.517147	1.523388
C	7.294132	1.219132	0.508411
O	6.94239	-0.151540.417035	
C	2.701442	1.202156	0.390794
C	1.629712	1.875582	1.273164
C	3.984345	1.130112	1.251573
O	4.035327	0.484556	2.309586
C	2.44336	-2.242992	-1.23102
C	2.758141	-2.338124	-2.746064
O	2.307644	-1.210821	-3.48857
N	-1.736745	-3.798745	0.24975
C	-1.176195	-3.573767	-1.087972
C	-1.934994	-2.448322	-1.841228
C	-3.677888	-4.132004	1.70828
C	-3.003506	-4.330606	0.35645
O	-3.57383-4.897904	-0.59189	
C	1.232626	-2.851617	3.821789
C	1.360919	-2.082291	2.503242
H	2.204606	-3.313024	4.079556

H	0.47966	-3.656074	3.746077
H	0.940174	-2.183829	4.651786
H	2.13334	-1.295881	2.569536
H	1.655913	-2.767085	1.689513
H	-3.716255	6.330475	-1.173765
H	-4.697599	5.12279	-1.881841
H	-1.446784	5.870338	-0.847976
H	-0.870316	4.147185	-0.70814
H	-2.333592.505136	-1.484526	
H	-4.377232	1.531751	-1.593264
H	-4.946656	2.948864	-2.51697
H	-5.101641	2.936584	-0.722801
H	-7.338975	3.189856	2.482398
H	-7.605494	1.422695	2.406565
H	-7.0794	2.330953	0.939089
H	-5.230984	2.543844	3.247044
H	-4.668907	-0.388052	0.474473
H	-6.324568	0.127564	0.901808
H	-2.979532	0.229868	1.705181
H	-2.962123	1.759667	2.567457
H	-4.337118	-4.988535	1.920936
H	-4.296604	-3.218758	1.652189
H	-2.956318	-4.003035	2.532956
H	-1.420315	-3.153391	0.992151
H	-1.444323	-2.201132	-2.797714
H	-2.97386-2.77539-2.025057		
H	-1.39484-4.494146	-1.656901	
H	2.92011	-3.115714	-0.740912
H	3.854162	-2.389977	-2.871702
H	2.320139	-3.288251	-3.119906
H	6.239588	1.556648	2.372119
H	7.714274	3.612053	1.982172
H	5.989308	4.056801	2.156407
H	6.748618	4.015267	0.535794
H	7.362425	1.705227	-0.495057
H	8.304524	1.259126	0.956673
H	2.890779	1.854436	-0.488801
H	1.523365	1.312068	2.217209
H	1.900168	2.920035	1.506452
H	6.033766	-0.215720.029009	
H	4.871033	2.329787	-0.14719
H	1.391332	-0.986733	-3.177142
H	2.410615	2.296615	-2.66474
H	1.040062	2.74553	-1.967751
H	-0.739140.397367	-2.628088	
H	0.754641	1.032296	-2.642362

Table S21. Optimized coordinates for NHaseAq, BP86+10% HF, S=1/2.

Fe	0.286871	-0.508983	-0.279727
S	-0.023985	-1.342845	1.771031
S	-0.161382	1.587609	0.670037
S	-1.809117	-0.769737	-0.872691
O	-0.143872.75158	-0.452904	

O	-2.210657	0.356809	-1.841869
O	-2.861066	-0.942970.229645	
N	0.941673	-2.144872	-1.045854
C	0.350546	-3.362871	-1.172373
O	0.972889	-4.426957	-1.322482
N	2.145996	-0.112197	0.008124
C	3.035383	-0.906096	-0.596495
O	4.26462	-0.691565	-0.707035
O	0.444093	0.366153	-2.154931
O	1.47829	2.734409	-2.477453
C	-6.974195	2.068416	2.088064
N	-5.577911.871809	2.447839	
C	-4.779497	0.965134	1.852458
N	-5.255853	0.161066	0.891603
N	-3.490550.855796	2.210519	
C	-4.564086	2.502637	-1.578123
N	-3.234646	3.050887	-1.382229
C	-2.994364.358597	-1.230985	
N	-4.001243	5.243821	-1.45329
N	-1.809665	4.768292	-0.771264
N	4.846102	1.992357	0.781882
C	6.221417	2.079529	1.264078
C	6.677827	3.542165	1.236932
C	7.12308	1.161694	0.412403
O	6.766609	-0.198858	0.486339
C	2.572286	1.18719	0.514725
C	1.489021	1.734869	1.443349
C	3.893647	1.171772	1.312831
O	4.003826	0.579314	2.390374
C	2.411751	-2.163067	-1.209001
C	2.801258	-2.263258	-2.69666
O	2.385494	-1.139502	-3.459495
N	-1.738136	-3.759691	0.125621
C	-1.197913	-3.456107	-1.196686
C	-1.931369	-2.281091	-1.86454
C	-3.59012-4.264236	1.633961	
C	-2.995035	-4.286946	0.23603
O	-3.620128	-4.730283	-0.736792
C	1.353189	-2.923535	3.630374
C	1.556975	-2.107537	2.348316
H	2.314701	-3.360659	3.944544
H	0.637206	-3.745644	3.474855
H	0.979762	-2.295037	4.454382
H	2.302194	-1.311918	2.493133
H	1.917302	-2.760359	1.540393
H	-3.795825	6.235788	-1.391082
H	-4.731287	4.995314	-2.113883
H	-1.530403	5.737304	-0.883633
H	-1.088631	4.028628	-0.520976
H	-2.455186	2.381205	-1.34689
H	-4.479415	1.415704	-1.489118
H	-4.965003	2.731091	-2.581216
H	-5.262266	2.884007	-0.817567
H	-7.325644	2.990185	2.565727

H	-7.616861	1.237223	2.423916
H	-7.072932.188275	0.999279	
H	-5.237443	2.300342	3.303099
H	-4.571469	-0.455359	0.417655
H	-6.24938-0.022421	0.809135	
H	-2.917232	0.195839	1.655642
H	-3.018759	1.630276	2.66639
H	-4.206803	-5.160894	1.780809
H	-4.241884	-3.380847	1.722214
H	-2.827558	-4.206508	2.423953
H	-1.360515	-3.211984	0.907752
H	-1.49328-2.045904	-2.845131	
H	-3.001511	-2.510117	-1.976517
H	-1.405736	-4.335958	-1.822329
H	2.840746	-3.046448	-0.70285
H	3.899363	-2.31347-2.762321	
H	2.383888	-3.205595	-3.097325
H	6.205058	1.692008	2.293345
H	7.706238	3.638478	1.618894
H	6.018264	4.167649	1.856095
H	6.664576	3.941426	0.208583
H	7.117277	1.540929	-0.634124
H	8.161137	1.244008	0.776474
H	2.686951	1.888161	-0.334294
H	1.458758	1.147589	2.371702
H	1.645625	2.795818	1.688239
H	5.860582	-0.310172	0.106643
H	4.70403 2.270172	-0.187257	
H	1.509037	-0.856266	-3.111088
H	2.411757	2.894653	-2.251784
H	0.944746	2.885238	-1.626003
H	-0.475346	0.633927	-2.410108
H	1.034249	1.178	-2.308246

Table S22. Optimized coordinates for NHaseAq, BP86+10% HF, S=3/2.

Fe	0.326281	-0.507507	-0.258845
S	-0.209664	-1.324874	1.992508
S	-0.103452	1.653578	0.562099
S	-1.865683	-0.743709	-0.96726
O	-0.045526	2.758479	-0.612175
O	-2.279420.34288	-1.964006	
O	-2.888963	-0.898990.156734	
N	0.944991	-2.152145	-1.032642
C	0.334358	-3.365142	-1.148295
O	0.946126	-4.435443	-1.280628
N	2.148532	-0.087557	-0.058835
C	3.043604	-0.927485	-0.633531
O	4.265855	-0.718876	-0.717492
O	0.368302	0.343421	-2.526119
O	1.502995	2.734489	-2.713069
C	-6.972743	2.100365	2.091692
N	-5.584561	1.884392	2.479724
C	-4.783290.981556	1.880943	

N	-5.257579	0.207	0.895149
N	-3.500518	0.848753	2.249501
C	-4.501252.547606	-1.531825	
N	-3.165076	3.090499	-1.365364
C	-2.922649	4.387751	-1.143278
N	-3.951025	5.272608	-1.238005
N	-1.713122	4.786051	-0.745815
N	4.897602	1.951677	0.827685
C	6.246004	2.011675	1.385918
C	6.702336	3.471705	1.470007
C	7.190968	1.140636	0.533646
O	6.826938	-0.221650.51209	
C	2.61709	1.203012	0.45081
C	1.534873	1.781429	1.358368
C	3.913723	1.126227	1.285452
O	3.975963	0.478527	2.333556
C	2.409465	-2.187913	-1.215623
C	2.786163	-2.309781	-2.70745
O	2.433391	-1.170389	-3.471651
N	-1.774057	-3.696384	0.145875
C	-1.21458-3.441148	-1.177423	
C	-1.935782	-2.291222	-1.904716
C	-3.631988	-4.255051.634352	
C	-3.014559	-4.261810.245797	
O	-3.607181	-4.746813	-0.728337
C	1.289397	-2.971894	3.720727
C	1.387925	-2.152712.432939	
H	2.260626	-3.446963.939393	
H	0.531378	-3.765791	3.631453
H	1.018214	-2.337229	4.579189
H	2.167645	-1.380277	2.516746
H	1.663749	-2.808936	1.593481
H	-3.743954	6.261248	-1.137273
H	-4.719018	5.057325	-1.866766
H	-1.462278	5.767756	-0.797397
H	-0.965554	4.044091	-0.592406
H	-2.381843	2.428093	-1.404259
H	-4.403401	1.458212	-1.54814
H	-4.960405	2.862985	-2.48511
H	-5.155742	2.850809	-0.70066
H	-7.326777	3.014674	2.581528
H	-7.628812	1.267668	2.396158
H	-7.045485	2.243481	1.003866
H	-5.258869	2.295724	3.34906
H	-4.579474	-0.401884	0.404571
H	-6.252584	0.0435	0.793258
H	-2.923984	0.193099	1.698781
H	-3.027922	1.587646	2.75951
H	-3.929643	-5.279094	1.902978
H	-4.544824	-3.641113	1.608561
H	-2.95873-3.856638	2.406465	
H	-1.419955	-3.116412	0.920623
H	-1.464191	-2.078074	-2.875049
H	-2.9984	-2.538883	-2.046754

H	-1.424834	-4.341184	-1.772991
H	2.838652	-3.061724	-0.694237
H	3.880086	-2.418506	-2.776644
H	2.322231	-3.234064	-3.09949
H	6.17461	1.565672	2.388618
H	7.711108	3.540655	1.906074
H	6.014063	4.059078	2.095123
H	6.737841	3.934315	0.469014
H	7.244043	1.576236	-0.488977
H	8.20719	1.192843	0.958236
H	2.775868	1.889537	-0.401927
H	1.472679	1.194261	2.286376
H	1.711987	2.839048	1.603476
H	5.929892	-0.303030.112901	
H	4.789176	2.311679	-0.118289
H	1.526928	-0.880083	-3.203659
H	2.452229	2.678931	-2.503005
H	1.024784	2.841846	-1.828116
H	-0.572369	0.551445	-2.727613
H	0.892996	1.187104	-2.687434

Table S23. Optimized coordinates for NHaseAq, BP86+10% HF, S=5/2.

Fe	0.325462	-0.569504	-0.216789
S	-0.224814	-1.281467	1.990789
S	-0.025506	1.871786	0.542366
S	-2.059026	-0.849216	-0.915296
O	0.087614	2.945616	-0.674224
O	-2.507911	0.181121	-1.964019
O	-3.115788	-1.082409	0.171978
N	0.974301	-2.258273	-1.002277
C	0.383967	-3.483537	-1.077379
O	0.992621	-4.557266	-1.176593
N	2.217561	-0.081226	-0.068199
C	3.081076	-0.943678	-0.655263
O	4.304926	-0.763905	-0.789526
O	0.191556	0.237075	-2.329389
O	1.44558	2.468795	-2.813602
C	-6.966109	2.229469	2.042516
N	-5.557493	2.05619	2.398041
C	-4.784117	1.091991	1.856438
N	-5.305768	0.255828	0.950243
N	-3.494529	0.948538	2.200537
C	-4.471119	2.618227	-1.571631
N	-3.129907	3.152638	-1.414134
C	-2.880794	4.450592	-1.194315
N	-3.920495	5.328176	-1.257783
N	-1.664299	4.859232	-0.840731
N	4.971061	1.9464	0.780466
C	6.308981	2.022742	1.354994
C	6.722228	3.490951	1.483358
C	7.27366	1.192396	0.48511
O	6.910992	-0.168881	0.403135
C	2.695636	1.207767	0.39649

C	1.632068	1.863722	1.293825
C	3.981952	1.1323	1.240553
O	4.039107	0.485535	2.290147
C	2.435294	-2.227399	-1.219693
C	2.753588	-2.327882	-2.725604
O	2.276767	-1.214605	-3.464169
N	-1.735811	-3.787310.227768	
C	-1.174837	-3.548894	-1.100804
C	-1.93087-2.421067	-1.843148	
C	-3.671201	-4.155545	1.673783
C	-2.998484	-4.314105	0.322257
O	-3.568314	-4.850475	-0.636547
C	1.248055	-2.886594	3.773824
C	1.370251	-2.097859	2.470724
H	2.216129	-3.355502	4.016476
H	0.49248	-3.682987	3.689495
H	0.96481	-2.233055	4.613694
H	2.145515	-1.320473	2.545839
H	1.655809	-2.770386	1.648186
H	-3.710926	6.318977	-1.185396
H	-4.702217	5.10497	-1.866839
H	-1.450156	5.850982	-0.844198
H	-0.882851	4.1347	-0.693125
H	-2.347357	2.496727	-1.458886
H	-4.378412	1.527548	-1.593345
H	-4.935393	2.941134	-2.519763
H	-5.115224	2.923424	-0.732952
H	-7.319413.16147	2.498004	
H	-7.595191.401678	2.409803	
H	-7.074152	2.316969	0.951891
H	-5.218075	2.511523	3.239875
H	-4.659843	-0.398749	0.466421
H	-6.308606	0.136047	0.868887
H	-2.972756	0.208226	1.711998
H	-2.969799	1.714719	2.609905
H	-4.32202-5.019453	1.862574	
H	-4.294417	-3.248666	1.639475
H	-2.952622	-4.042942	2.498547
H	-1.414394	-3.164799	0.980594
H	-1.434377	-2.161836	-2.78939
H	-2.963457	-2.748323	-2.040195
H	-1.385842	-4.459819	-1.679426
H	2.908983	-3.090665	-0.719131
H	3.846181	-2.356415	-2.853782
H	2.334157	-3.281592	-3.098159
H	6.236735	1.548199	2.34449
H	7.726444	3.578837	1.927038
H	6.013243	4.038652	2.121064
H	6.748531	3.98289	0.496206
H	7.341673	1.668721	-0.518409
H	8.282807	1.227734	0.927385
H	2.872783	1.867245	-0.474912
H	1.534768	1.287491	2.226866
H	1.903614	2.900876	1.541087

H	6.007209	-0.235108	0.016455
H	4.858828	2.326596	-0.156918
H	1.368916	-1.001984	-3.142692
H	2.398767	2.354593	-2.648454
H	1.029966	2.755874	-1.927557
H	-0.744556	0.442736	-2.564124
H	0.756309	1.044924	-2.585129

Table S24. Optimized coordinates for NHaseAq, B3LYP, S=1/2.

Fe	0.304004	-0.510011	-0.284212
S	-0.029827	-1.312537	1.805635
S	-0.158993	1.645469	0.613131
S	-1.854556	-0.80053-0.896414	
O	-0.140351	2.758056	-0.553797
O	-2.255464	0.295417	-1.884628
O	-2.897469	-0.968683	0.203232
N	0.966413	-2.175385	-1.018267
C	0.379008	-3.391643	-1.111918
O	0.995664	-4.458413	-1.218632
N	2.163062	-0.096297	-0.015489
C	3.051257	-0.903779	-0.596946
O	4.273886	-0.688034	-0.71116
O	0.430341	0.314842	-2.218068
O	1.505883	2.694346	-2.592095
C	-7.000574	2.085245	2.071778
N	-5.597082	1.90725 2.421347	
C	-4.803024	0.984219	1.852937
N	-5.281138	0.159479	0.916664
N	-3.518764	0.875906	2.215561
C	-4.564099	2.52 -1.576884	
N	-3.233522	3.076017	-1.397105
C	-2.998924	4.377822	-1.219263
N	-4.018399	5.258451	-1.375154
N	-1.801475	4.792989	-0.812601
N	4.852958	2.016897	0.786768
C	6.216967	2.105976	1.299983
C	6.655714	3.573246	1.320008
C	7.146907	1.218012	0.450602
O	6.806954	-0.148125	0.494801
C	2.586877	1.210775	0.470932
C	1.496632	1.791986	1.375064
C	3.892304	1.195641	1.291178
O	3.986091	0.592239	2.358019
C	2.438172	-2.186674	-1.168309
C	2.844651	-2.346108	-2.645066
O	2.415115	-1.264004	-3.459809
N	-1.731649	-3.788041	0.155446
C	-1.171705	-3.492859	-1.161268
C	-1.911387	-2.336818	-1.858278
C	-3.606428	-4.243994	1.646139
C	-2.989719	-4.305811	0.259894
O	-3.603591	-4.763775	-0.706385
C	1.320635	-2.896814	3.678012

C	1.532992	-2.119769	2.375369
H	2.266181	-3.356783	3.989941
H	0.5792	-3.693975	3.553189
H	0.981419	-2.239758	4.487144
H	2.299015	-1.34977	2.495192
H	1.8594	-2.800746	1.5854
H	-3.820444	6.24593	-1.294667
H	-4.780634	5.020541	-1.994391
H	-1.555684	5.770353	-0.879246
H	-1.066496	4.06878	-0.600768
H	-2.449376	2.4231	-1.389717
H	-4.462882	1.436877	-1.557353
H	-5.000141	2.803952	-2.543727
H	-5.234939	2.84428	-0.774474
H	-7.356524	2.998595	2.549563
H	-7.623515	1.249724	2.414262
H	-7.111293	2.201053	0.989419
H	-5.254155	2.361858	3.256161
H	-4.612156	-0.457535	0.438796
H	-6.272834	0.029885	0.788004
H	-2.944146	0.208348	1.690356
H	-3.052522	1.634372	2.692756
H	-4.26849	-5.101656	1.786192
H	-4.205895	-3.329008	1.717675
H	-2.857427	-4.222361	2.443346
H	-1.366822	-3.242669	0.934941
H	-1.455335	-2.105992	-2.824701
H	-2.966328	-2.590106	-1.997216
H	-1.356313	-4.375364	-1.778024
H	2.870501	-3.036322	-0.623448
H	3.937143	-2.377285	-2.7039
H	2.448814	-3.303121	-3.011502
H	6.185945	1.698519	2.313833
H	7.671106	3.671846	1.720309
H	5.980642	4.169284	1.941924
H	6.653563	3.999209	0.30828
H	7.15649	1.607112	-0.584661
H	8.170676	1.303992	0.834769
H	2.723376	1.8878	-0.384953
H	1.455994	1.236019	2.314255
H	1.666196	2.851422	1.589913
H	5.914998	-0.272842	0.106316
H	4.726454	2.317102	-0.17199
H	1.526233	-0.998763	-3.155083
H	2.430152	2.87243	-2.361897
H	0.967149	2.863656	-1.762227
H	-0.475205	0.593001	-2.474631
H	1.032004	1.093162	-2.397297

Table S25. Optimized coordinates for NHaseAq, B3LYP, S=3/2.

Fe	0.33333	-0.509424	-0.269289
S	-0.213412	-1.296372	2.021403
S	-0.090541	1.701157	0.517157

S	-1.900644	-0.768265	-0.991619
O	-0.033431	2.765252	-0.684848
O	-2.311756	0.292896	-2.005491
O	-2.92231	-0.910094	0.126174
N	0.949018	-2.179929	-1.018528
C	0.337883	-3.389799	-1.113775
O	0.943739	-4.459414	-1.224239
N	2.165617	-0.086906	-0.074747
C	3.051727	-0.943266	-0.628132
O	4.271164	-0.749203	-0.710089
O	0.35781	0.319018	-2.556963
O	1.544728	2.713604	-2.796843
C	-6.988634	2.12574	2.083399
N	-5.596531	1.917217	2.466048
C	-4.799468	1.004173	1.884865
N	-5.275052	0.210854	0.92047
N	-3.520022	0.877101	2.254927
C	-4.499557	2.564111	-1.529125
N	-3.16275	3.111746	-1.370412
C	-2.920768	4.403176	-1.13629
N	-3.951293	5.285068	-1.185323
N	-1.703129	4.80712	-0.784662
N	4.911409	1.956101	0.824216
C	6.249039	2.018912	1.408075
C	6.690739	3.480919	1.524016
C	7.216872	1.167136	0.565252
O	6.866103	-0.198145	0.524831
C	2.637044	1.209162	0.414628
C	1.55419	1.824353	1.300075
C	3.92051	1.13688	1.267043
O	3.96927	0.491784	2.311144
C	2.416392	-2.214626	-1.189551
C	2.8067	-2.369274	-2.673595
O	2.455809	-1.247368	-3.465675
N	-1.780464	-3.724091	0.167658
C	-1.21282	-3.472566	-1.15411
C	-1.93779	-2.336147	-1.899806
C	-3.652839	-4.234122	1.652583
C	-3.021411	-4.281619	0.271464
O	-3.61222	-4.77323	-0.69339
C	1.260061	-2.952871	3.760016
C	1.380129	-2.127159	2.480898
H	2.222509	-3.427111	3.992662
H	0.508144	-3.743027	3.65417
H	0.975097	-2.327496	4.613924
H	2.154511	-1.362135	2.583746
H	1.668185	-2.777666	1.649826
H	-3.744615	6.269919	-1.091692
H	-4.747748	5.070414	-1.7693
H	-1.477139	5.791199	-0.781364
H	-0.949221	4.082722	-0.658709
H	-2.378942	2.463066	-1.431392
H	-4.397099	1.482169	-1.577223
H	-4.972822	2.903922	-2.459852

H	-5.137347	2.839357	-0.683102
H	-7.345629	3.027294	2.582303
H	-7.631086	1.288037	2.381349
H	-7.067396	2.278715	1.003032
H	-5.266944	2.348383	3.318495
H	-4.608907	-0.393470.423119	
H	-6.266606	0.096041	0.778114
H	-2.941606	0.210632	1.736809
H	-3.0565 1.600594	2.785497	
H	-4.182698	-5.172021.837979	
H	-4.387222	-3.420604	1.670629
H	-2.925061	-4.056872	2.449393
H	-1.431765	-3.151713	0.939316
H	-1.463014	-2.13434-2.864074	
H	-2.989957	-2.595446	-2.049654
H	-1.406656	-4.372593	-1.741945
H	2.84198 -3.068979	-0.648086	
H	3.894928	-2.47238-2.7347	
H	2.351204	-3.294148	-3.053419
H	6.165452	1.564391	2.398558
H	7.685428	3.551476	1.978233
H	5.988401	4.047553	2.143116
H	6.73974 3.95927 0.537152		
H	7.28329 1.604719	-0.448054	
H	8.219334	1.222075	1.005865
H	2.814957	1.872148	-0.443919
H	1.484252	1.273077	2.241022
H	1.74521 2.880779	1.511409	
H	5.981767	-0.292924	0.117519
H	4.817119	2.323022	-0.114668
H	1.546478	-0.968059	-3.23202
H	2.488185	2.670213	-2.578712
H	1.062088	2.841908	-1.93047
H	-0.570229	0.536753	-2.770317
H	0.898078	1.132405	-2.742134

Table S26. Optimized coordinates for NHaseAq, B3LYP, S=5/2.

Fe	0.325362	-0.564311	-0.219798
S	-0.218351	-1.245522.017985	
S	-0.016781	1.90555 0.513121	
S	-2.07975-0.876654	-0.943996	
O	0.083357	2.929118	-0.739465
O	-2.519452	0.139046	-2.0009
O	-3.135747	-1.088834	0.138326
N	0.961799	-2.280414	-0.98613
C	0.369756	-3.500641	-1.060403
O	0.974128	-4.570897	-1.157401
N	2.223639	-0.082886	-0.08268
C	3.077709	-0.961935	-0.649301
O	4.298454	-0.796461	-0.778917
O	0.189757	0.229299	-2.345963
O	1.496181	2.4583 -2.876944	
C	-6.970443	2.261529	2.038821

N	-5.560794	2.087326	2.391094
C	-4.790349	1.118829	1.862697
N	-5.309870.267957		0.974959
N	-3.502458	0.987706	2.205118
C	-4.463833	2.633762	-1.568974
N	-3.121575	3.170182	-1.41226
C	-2.870772	4.463873	-1.192767
N	-3.906854	5.342531	-1.22837
N	-1.648825	4.876238	-0.876512
N	4.981479	1.948962	0.777376
C	6.312674	2.024129	1.369309
C	6.722172	3.491986	1.51659
C	7.292269	1.202871	0.51039
O	6.941075	-0.161042	0.423434
C	2.710968	1.209877	0.36484
C	1.652923	1.901904	1.243335
C	3.988242	1.135447	1.220827
O	4.035784	0.486784	2.263454
C	2.427615	-2.254208	-1.192583
C	2.7639 -2.389906		-2.690844
O	2.295632	-1.293363	-3.460894
N	-1.753658	-3.800551	0.243916
C	-1.190018	-3.576093	-1.087423
C	-1.947734	-2.461429	-1.848211
C	-3.688181	-4.131624	1.69966
C	-3.015504	-4.319462	0.352085
O	-3.588852	-4.869387	-0.589654
C	1.227515	-2.8667 3.810435	
C	1.36406 -2.091056		2.502384
H	2.18198 -3.349534.055554		
H	0.461227	-3.645962	3.733563
H	0.958493	-2.205386	4.641772
H	2.149262	-1.333665	2.573189
H	1.63355 -2.773884		1.691917
H	-3.696613	6.329217	-1.166415
H	-4.714145.117726		-1.793418
H	-1.447528	5.865367	-0.845479
H	-0.869834.163035		-0.751492
H	-2.338673	2.525146	-1.471794
H	-4.370433	1.549959	-1.612393
H	-4.933003	2.974589	-2.501208
H	-5.097636	2.919946	-0.723384
H	-7.322026	3.183672	2.502424
H	-7.591859	1.432796	2.399014
H	-7.082005	2.35787 0.954981	
H	-5.214632	2.564061	3.212226
H	-4.673509	-0.382193	0.488876
H	-6.307622	0.178106	0.861918
H	-2.971672	0.245457	1.748165
H	-2.992409	1.740883	2.644154
H	-4.365261	-4.968228	1.886767
H	-4.275326	-3.207469	1.659261
H	-2.972374	-4.042446	2.522391
H	-1.431371	-3.182367	0.988505

H	-1.450973	-2.217841	-2.791304
H	-2.972577	-2.790577	-2.045675
H	-1.387781	-4.489555	-1.652515
H	2.894431	-3.099052	-0.67028
H	3.85114	-2.417974	-2.807514
H	2.350908	-3.342671	-3.050557
H	6.232242	1.548173	2.349984
H	7.71519	3.576345	1.972493
H	6.006927	4.028552	2.147486
H	6.759826	3.992121	0.540148
H	7.367854	1.671942	-0.488112
H	8.290434	1.242807	0.961871
H	2.904473	1.845188	-0.511957
H	1.557584	1.364946	2.19163
H	1.935323	2.93857	1.450725
H	6.050325	-0.242983	0.027506
H	4.880345	2.337185	-0.152003
H	1.384307	-1.08585	-3.174854
H	2.444746	2.360088	-2.702157
H	1.072167	2.750877	-2.012199
H	-0.733325	0.442937	-2.593576
H	0.76801	1.00138	-2.618632

Table S27. Optimized coordinates for Acetonitrile-bound NHase, S=1/2.

Fe	-0.213393	-0.550705	0.215241
S	0.148469	-1.471012	-1.80308
S	0.194422	1.508565	-0.803283
S	1.892274	-0.723301	0.813629
O	0.138223	2.731582	0.251626
O	2.397099	0.439864	1.670802
O	2.910361	-1.026507	-0.325969
N	-0.858467	-2.203943	1.053453
C	-0.219752	-3.403923	1.207497
O	-0.807825	-4.499466	1.31626
N	-2.10057	-0.268237	-0.187605
C	-2.976366	-1.040693	0.467479
O	-4.214549	-0.830023	0.590647
N	-0.581339	0.407254	1.857398
C	-0.972511	0.979552	2.791155
O	-1.999296	3.753039	1.556186
C	-1.526083	1.692385	3.925291
C	7.026387	2.097593	-2.119759
N	5.6346	1.880508	-2.488491
C	4.850135	0.956811	-1.890932
N	5.31791	0.177184	-0.897973
N	3.568195	0.796252	-2.281714
C	4.529895	2.608202	1.478063
N	3.192153	3.116554	1.224632
C	2.930182	4.417171	1.033189
N	3.909951	5.338597	1.285605
N	1.762004	4.793313	0.489727
N	-4.824062	1.806648	-1.016791
C	-6.1948	1.850227	-1.533218

C	-6.668086	3.31007 -1.604008	
C	-7.102571	0.95994 -0.648539	
O	-6.723444	-0.404207	-0.650758
C	-2.538623	1.034259	-0.695461
C	-1.453465	1.59144 -1.622414	
C	-3.848570.982122		-1.51571
O	-3.938576	0.361947	-2.587222
C	-2.343465	-2.298145	1.09122
C	-2.864274	-2.541178	2.528652
O	-2.270892	-1.658968	3.492984
N	1.891686	-3.794835	-0.022033
C	1.331485	-3.432228	1.283573
C	2.019061	-2.193828	1.895342
C	3.763719	-4.285773	-1.512317
C	3.167142	-4.296821	-0.108405
O	3.799255	-4.707622	0.881436
C	-1.16208-3.12073-3.656757		
C	-1.416461	-2.263383	-2.408974
H	-2.113256	-3.571542	-3.995276
H	-0.450065	-3.939355	-3.449103
H	-0.756993	-2.51691-4.488996	
H	-2.163347	-1.474918	-2.604922
H	-1.804126	-2.891836	-1.590178
H	3.654696	6.323429	1.223322
H	4.582112	5.125277	2.022424
H	1.439244	5.751302	0.615115
H	1.056508	4.022358	0.2652
H	2.432832	2.414984	1.177271
H	4.475449	1.514949	1.404076
H	4.889735	2.864908	2.494712
H	5.247175	3.003126	0.736679
H	7.375129	3.018837	-2.608734
H	7.684474	1.265822	-2.437447
H	7.114747	2.234418	-1.028347
H	5.303068	2.270325	-3.369645
H	4.620941	-0.45427-0.44564	
H	6.313873	-0.007288	-0.800466
H	3.006974	0.12375 -1.71321	
H	3.071892	1.569328	-2.723109
H	4.437885	-5.14886-1.631637	
H	4.357612	-3.36199-1.633839	
H	2.996234	-4.303168	-2.304511
H	1.528319	-3.259351	-0.824702
H	1.552201	-1.911862.854158	
H	3.094414	-2.389905	2.048542
H	1.563423	-4.274821	1.958079
H	-2.678698	-3.1729 0.495698	
H	-3.950814	-2.351193	2.537845
H	-2.673291	-3.597957	2.798171
H	-6.147994	1.403749	-2.541299
H	-7.690828	3.371925	-2.017525
H	-5.997924	3.906629	-2.245969
H	-6.684042	3.77243 -0.598591	
H	-7.121333	1.392671	0.381531

H	-8.138615	1.006774	-1.035269
H	-2.666384	1.740033	0.154296
H	-1.387461	0.986087	-2.541683
H	-1.631045	2.647403	-1.890913
H	-5.812333	-0.475352	-0.259625
H	-4.706927	2.094246	-0.042371
H	-1.31763	-1.649074	3.249252
H	-2.563874	4.057486	0.818439
H	-1.228874	3.306106	1.087161
H	-0.737	1.965281	4.646632
H	-2.27064	1.043315	4.417625
H	-2.012523	2.605324	3.533086

Table S28. Optimized coordinates for Acetonitrile-bound NHase, S=3/2.

Fe	-0.205364	-0.571059	0.158665
S	0.425846	-1.493077	-1.997467
S	0.135424	1.576076	-0.742077
S	1.947311	-0.632082	0.967517
O	-0.047483	2.76218	0.343763
O	2.364677	0.521421	1.872942
O	2.980451	-0.867917	-0.16448
N	-0.790181	-2.207266	1.051892
C	-0.108548	-3.390713	1.190707
O	-0.658119	-4.507458	1.247871
N	-2.043958	-0.275348	-0.141325
C	-2.923692	-1.112815	0.470187
O	-4.157672	-0.928022	0.551091
N	-0.624749	0.57959	2.399604
C	-1.379596	1.107441	3.113647
O	-2.408238	3.62648	1.366228
C	-2.364143	1.76317	3.958914
C	7.065213	2.213204	-2.021137
N	5.702201	1.933922	-2.45667
C	4.909971	1.023698	-1.84883
N	5.370755	0.290245	-0.817986
N	3.637822	0.835578	-2.256053
C	4.441724	2.70505	1.487928
N	3.090619	3.191954	1.266773
C	2.815495	4.470275	0.969732
N	3.816225	5.400018	1.055474
N	1.608267	4.807179	0.494197
N	-4.842907	1.669656	-1.149146
C	-6.193994	1.663187	-1.715783
C	-6.663367	3.105587	-1.96204
C	-7.137698	0.874503	-0.775239
O	-6.768732	-0.485411	-0.620372
C	-2.554887	0.984754	-0.708253
C	-1.476652	1.560621	-1.627272
C	-3.841519	0.823235	-1.548759
O	-3.896249	0.100002	-2.555497
C	-2.266687	-2.366424	1.059101
C	-2.81389	-2.675451	2.470934
O	-2.380429	-1.728603	3.453446

N	2.049268	-3.680695	0.014014
C	1.440934	-3.369962	1.309645
C	2.071901	-2.136958	1.994149
C	3.934717	-4.238611	-1.442161
C	3.312673	-4.210889	-0.047306
O	3.91801	-4.632174	0.955824
C	-0.944609	-3.21531	-3.758268
C	-1.123778	-2.398426	-2.475408
H	-1.887734	-3.738218	-4.007394
H	-0.151403	-3.975769	-3.644478
H	-0.677095	-2.568243	-4.613072
H	-1.940181	-1.661819	-2.580949
H	-1.387978	-3.068411	-1.638827
H	3.567654	6.375504	0.897054
H	4.541003	5.257822	1.758804
H	1.30998	5.780272	0.531709
H	0.886521	4.027071	0.345389
H	2.33669	2.487007	1.31824
H	4.386913	1.609757	1.513189
H	4.855181	3.050007	2.456907
H	5.116231	3.031789	0.676844
H	7.411813	3.123131	-2.532067
H	7.7648	1.391348	-2.267431
H	7.088136	2.397001	-0.933622
H	5.39032	2.30851	-3.351128
H	4.679084	-0.324741	-0.339506
H	6.367626	0.127541	-0.69621
H	3.067463	0.18824	-1.673715
H	3.146833	1.57648	-2.754289
H	4.410677	-5.219191	-1.608789
H	4.725515	-3.469044	-1.492778
H	3.205614	-4.041526	-2.245736
H	1.691325	-3.150405	-0.798624
H	1.558364	-1.90167	2.941712
H	3.145607	-2.312481	2.181092
H	1.678562	-4.225754	1.965749
H	-2.557622	-3.224218	0.417636
H	-3.915859	-2.624005	2.432608
H	-2.509201	-3.703401	2.74697
H	-6.113101	1.111851	-2.668668
H	-7.666649	3.116188	-2.424467
H	-5.966236	3.632557	-2.635397
H	-6.724782	3.67425	-1.014621
H	-7.189326	1.41226	0.202699
H	-8.15915	0.879898	-1.200579
H	-2.737703	1.704285	0.116368
H	-1.343156	0.922203	-2.517338
H	-1.699758	2.594622	-1.942654
H	-5.852051	-0.517514	-0.247233
H	-4.731116	2.110966	-0.233232
H	-1.44349	-1.533391	3.225104
H	-2.678675	4.209171	0.628503
H	-1.534721	3.241791	1.044742
H	-1.904729	2.101717	4.90301

H -3.172398 1.046508 4.18272
H -2.766312.630079 3.40406

Table S29. Optimized coordinates for Acetonitrile-bound NHase, S=5/2.

Fe -0.297797 -0.658214 0.168435
S 0.244171 -1.49772-1.98205
S 0.06356 1.785094 -0.557443
S 2.104674 -0.781408 0.8663
O -0.043553 2.877998 0.652853
O 2.73063 0.252782 1.80384
O 3.06501 -1.163827 -0.298628
N -0.983186 -2.289877 1.060842
C -0.357506 -3.502058 1.178853
O -0.93892-4.599306 1.26293
N -2.19793-0.168038 -0.036281
C -3.080707 -1.008592 0.565096
O -4.312071 -0.812481 0.677053
N -0.268969 0.342734 2.330358
C -0.129866 1.207628 3.09853
O -2.326153 3.282327 2.073782
C 0.02542 2.284236 4.060899
C 6.992478 2.035303 -2.306478
N 5.582216 1.847612 -2.647126
C 4.811452 0.910015 -2.048584
N 5.325258 0.114603 -1.093467
N 3.513674 0.749747 -2.384124
C 4.478291 2.682405 1.257052
N 3.13248 3.212403 1.114771
C 2.886925 4.478976 0.738619
N 3.941091 5.343714 0.609492
N 1.64308 4.859826 0.429009
N -4.937842 1.826937 -1.063255
C -6.261034 1.85133 -1.688329
C -6.700677 3.306237 -1.909578
C -7.246008 1.044438 -0.806851
O -6.870382 -0.313888 -0.65017
C -2.675208 1.116004 -0.539089
C -1.571342 1.784633 -1.383833
C -3.919537 0.999978 -1.450184
O -3.929743 0.30899 -2.481317
C -2.462847 -2.300853 1.159627
C -2.914665 -2.471539 2.627459
O -2.410582 -1.431925 3.469971
N 1.775428 -3.864219 -0.060532
C 1.205858 -3.516668 1.245197
C 1.944945 -2.315032 1.895348
C 3.706458 -4.312363 -1.487885
C 3.044309 -4.393328 -0.118542
O 3.621716 -4.869392 0.875036
C -1.204097 -3.2106 -3.699738
C -1.357203 -2.320481 -2.461023
H -2.177677 -3.674452 -3.946744
H -0.472691 -4.019458 -3.525256

H	-0.870505	-2.628176	-4.577217
H	-2.113754	-1.530907	-2.616552
H	-1.68457-2.928302	-1.600069	
H	3.722906	6.33305 0.496617	
H	4.771216	5.166619	1.17471
H	1.441105	5.842877	0.258272
H	0.85147 4.121872	0.450548	
H	2.343266	2.561871	1.213104
H	4.379321	1.59867 1.409075	
H	4.99837 3.113089	2.136081	
H	5.080753	2.879829	0.352097
H	7.34298 2.95845 -2.790318		
H	7.627006	1.197575	-2.653745
H	7.105837	2.153386	-1.215639
H	5.239414	2.250121	-3.518346
H	4.659135	-0.516516	-0.591088
H	6.328133	-0.040783	-1.02515
H	2.991038	0.049489	-1.824017
H	2.98685 1.520479	-2.792212	
H	4.383189	-5.171055	-1.623372
H	4.303958	-3.384251	-1.526827
H	2.976434	-4.280138	-2.314446
H	1.465656	-3.2822 -0.855322	
H	1.437442	-1.981199	2.816233
H	2.979615	-2.621481	2.132406
H	1.433592	-4.375282	1.901264
H	-2.870242	-3.161363	0.589747
H	-4.01571-2.416942	2.669965	
H	-2.591847	-3.473991	2.975729
H	-6.144137	1.326331	-2.652212
H	-7.687168	3.345729	-2.405676
H	-5.972518	3.840852	-2.542638
H	-6.785929	3.848528	-0.94879
H	-7.351908	1.568865	0.173551
H	-8.244221	1.045394	-1.283877
H	-2.909715	1.786058	0.313457
H	-1.418362	1.214256	-2.318358
H	-1.835267	2.826004	-1.637427
H	-5.976025	-0.342129	-0.22561
H	-4.859317	2.259474	-0.140378
H	-1.474662	-1.302546	3.193762
H	-2.581156	2.397607	2.406395
H	-1.501385	3.091045	1.526695
H	1.055212	2.676839	4.020347
H	-0.184439	1.918339	5.080409
H	-0.6938 3.080238	3.792599	

Table S30. Optimized coordinates for H_2O attack on coordinated acetonitrile, transition state.

Fe	0.294889	0.497326	0.271357
S	-0.076904	1.421225	-1.816853
S	-0.149989	-1.395453	-0.966689
S	-1.824618	0.688123	0.839093
O	-0.196216	-2.798107	-0.042318

O	-2.362438	-0.474089	1.680619
O	-2.821161	1.000903	-0.317022
N	0.939316	2.179327	1.011766
C	0.300413	3.367653	1.210516
O	0.888557	4.458976	1.369874
N	2.172716	0.225818	-0.169363
C	3.063734	1.019034	0.441805
O	4.308713	0.837589	0.513128
N	0.591879	-0.433194	1.886689
C	0.831729	-1.315622.677839	
O	1.012555	-2.953153	2.066257
C	0.981114	-1.510184.148219	
C	-7.068551	-1.898251	-2.203732
N	-5.679268	-1.691883	-2.582029
C	-4.860757	-0.826029	-1.94853
N	-5.302995	-0.0496	-0.938873
N	-3.565756	-0.72533-2.312731	
C	-4.629307	-2.492274	1.420519
N	-3.313048	-3.036564	1.126562
C	-3.081624	-4.349105	0.989669
N	-4.066155	-5.237255	1.315554
N	-1.943477	-4.782329	0.419717
N	4.853747	-1.903769	-0.970774
C	6.221097	-2.043222	-1.470877
C	6.575228	-3.536277	-1.538961
C	7.174456	-1.220479	-0.569939
O	6.882997	0.165614	-0.571193
C	2.591111	-1.063706	-0.709579
C	1.529582	-1.566005	-1.693938
C	3.925397	-1.043328	-1.49089
O	4.059879	-0.426902	-2.559395
C	2.423437	2.261805	1.089075
C	2.895626	2.41735	2.551362
O	2.395468	1.376143	3.397112
N	-1.804446	3.750678	-0.0317
C	-1.251768	3.396863	1.277831
C	-1.943445	2.166508	1.906818
C	-3.616524.379754	-1.547476	
C	-3.047151	4.321252	-0.131991
O	-3.674469	4.756337	0.851315
C	1.294334	3.065793	-3.639619
C	1.507115	2.200599	-2.390407
H	2.257452	3.514271	-3.946537
H	0.579977	3.886651	-3.449164
H	0.911739	2.469074	-4.487579
H	2.253726	1.410074	-2.576294
H	1.881851	2.82138	-1.560088
H	-3.86121-6.232138	1.235997	
H	-4.708603	-4.989672.067475	
H	-1.62053-5.731273	0.600037	
H	-1.245865	-4.061010.102174	
H	-2.544586	-2.341533	1.098965
H	-4.580884	-1.411009	1.245306
H	-4.923563	-2.653523	2.477082

H	-5.392092	-2.948395	0.76526
H	-7.42687-2.821691		-2.682013
H	-7.723518	-1.065203	-2.524197
H	-7.149397	-2.021996	-1.110636
H	-5.34875-2.090978		-3.45927
H	-4.580372	0.542507	-0.474806
H	-6.285091	0.21087 -0.87548	
H	-2.975416	-0.096343	-1.722504
H	-3.108888	-1.507844	-2.7788
H	-4.433237	3.640889	-1.632199
H	-2.867472	4.161046	-2.326726
H	-4.049491	5.378236	-1.725185
H	-1.445691	3.205547	-0.830117
H	-1.468607	1.893168	2.864075
H	-3.017312.368888		2.063398
H	-1.485283	4.245783	1.943418
H	2.78188 3.158554		0.543563
H	3.999595	2.364192	2.56504
H	2.57956 3.414945		2.915731
H	6.21932 -1.5918 -2.477902		
H	7.594363	-3.682629	-1.940187
H	5.86568 -4.074472		-2.189852
H	6.541764	-3.999249	-0.534327
H	7.149706	-1.658279	0.458139
H	8.210917	-1.330982	-0.941597
H	2.678305	-1.801955	0.11944
H	1.554529	-0.962077	-2.614812
H	1.659089	-2.630947	-1.950205
H	5.958543	0.29353 -0.231444	
H	4.693702	-2.220109	-0.012289
H	1.538477	1.099934	2.991597
H	1.977628	-3.006968	1.885682
H	0.434511	-2.817343	0.940386
H	0.185505	-2.177441	4.522838
H	0.92051 -0.536986		4.660352
H	1.951287	-1.979055	4.387126

Table S31. Optimized coordinates for H_2O attack on coordinated acetonitrile, coordinated amidate tautomer + sulfenic acid (SOH).

Fe	0.302061	0.485533	0.258525
S	-0.144426	1.363456	-1.886673
S	-0.11944-1.246746		-1.169722
S	-1.791198	0.621077	0.88835
O	-0.167788	-2.838514	-0.349707
O	-2.279056	-0.532635	1.777208
O	-2.824830.870087		-0.255502
N	0.884186	2.20166 0.927424	
C	0.230045	3.36542 1.184321	
O	0.807272	4.448335	1.425348
N	2.176615	0.29296 -0.230394	
C	3.027626	1.03784 0.489277	
O	4.263198	0.838046	0.644197
N	0.725856	-0.408343	1.851878

C	1.123614	-1.410797	2.487325
O	1.34775	-2.683151.822282	
C	1.45493	-1.496512	3.964998
C	-7.071785	-2.033022	-2.079207
N	-5.696797	-1.798969	-2.492613
C	-4.878106	-0.922072	-1.872219
N	-5.302804	-0.180644	-0.830534
N	-3.601593	-0.776058	-2.282172
C	-4.581071	-2.516217	1.526473
N	-3.255174	-3.049721.263763	
C	-3.009935	-4.357354	1.114195
N	-4.005526	-5.259553	1.350653
N	-1.80501-4.765656		0.674133
N	4.871527	-1.825231	-0.98904
C	6.263702	-1.955566	-1.413923
C	6.602744	-3.447694	-1.546664
C	7.164767	-1.202357	-0.404762
O	6.879062	0.182463	-0.329686
C	2.612271	-0.98116-0.798059	
C	1.587013	-1.440829	-1.841713
C	3.976573	-0.93756-1.521215	
O	4.172289	-0.274214	-2.552054
C	2.347592	2.255342	1.148011
C	2.673402	2.35204	2.672952
O	1.705636	1.697438	3.497769
N	-1.897601	3.669977	-0.075295
C	-1.321497	3.364499	1.23543
C	-1.956907	2.126604	1.910357
C	-3.710548	4.309174	-1.590588
C	-3.132405	4.256812	-0.177253
O	-3.745832	4.720348	0.801808
C	1.195226	3.028708	-3.715119
C	1.425625	2.166578	-2.467279
H	2.149257	3.499278	-4.017489
H	0.462772	3.833711	-3.526213
H	0.829051	2.424812	-4.565144
H	2.190726	1.394116	-2.652737
H	1.785297	2.78433	-1.627802
H	-3.77502-6.251567		1.316765
H	-4.723704	-5.014732	2.032001
H	-1.625977	-5.758604	0.540078
H	-1.208185	-4.0741	0.160737
H	-2.501741	-2.333031.249164	
H	-4.506444	-1.425577	1.444338
H	-4.935736	-2.759546	2.548246
H	-5.313059	-2.899007	0.793583
H	-7.44072-2.932676		-2.593091
H	-7.741539	-1.189614	-2.335607
H	-7.118031	-2.214487	-0.991812
H	-5.388194	-2.17422-3.388162	
H	-4.577868	0.415593	-0.37418
H	-6.289749	0.038669	-0.713365
H	-3.000587	-0.163079	-1.684096
H	-3.148799	-1.522048	-2.80744

H	-4.038752	5.339239	-1.810156
H	-4.603412	3.660634	-1.634447
H	-2.998995	3.981218	-2.36672
H	-1.545092	3.115399	-0.8677
H	-1.454747	1.910547	2.868553
H	-3.037193	2.284515	2.073567
H	-1.578726	4.221246	1.881256
H	2.776379	3.162007	0.676065
H	3.692359	1.933635	2.819318
H	2.686969	3.415461	2.96917
H	6.329955	-1.447161	-2.391528
H	7.644207	-3.588336	-1.888528
H	5.930609	-3.935284	-2.272794
H	6.496429	-3.967047	-0.575224
H	7.074025	-1.708412	0.587866
H	8.221941	-1.296273	-0.718274
H	2.65892	-1.743798	0.007456
H	1.661735	-0.829058	-2.753996
H	1.699211	-2.505399	-2.104118
H	5.934243	0.296999	-0.043656
H	4.651004	-2.189317	-0.059925
H	1.253147	1.018661	2.917702
H	1.354115	-3.398153	2.496319
H	0.419021	-2.782666	0.468383
H	0.664388	-2.043687	4.514462
H	1.547979	-0.482984	4.383157
H	2.411373	-2.028544	4.123134

Table S32. Optimized coordinates for H_2O attack on coordinated acetonitrile, coordinated amide tautomer + sulfenate (Rotate, H^+ shift).

Fe	0.309714	0.432718	0.168601
S	0.030132	1.234749	-1.952313
S	-0.200204	-1.669241	-0.787478
S	-1.801009	0.755716	0.673708
O	-0.20924	-2.769563	0.408489
O	-2.424159	-0.318246	1.572838
O	-2.760323	1.039537	-0.525164
N	1.019705	2.096417	0.889292
C	0.429472	3.32915	0.945451
O	1.065384	4.405452	0.965409
N	2.1781	0.014887	-0.169768
C	3.091376	0.811444	0.389394
O	4.324275	0.575425	0.529041
N	0.43563	-0.636712	1.881213
C	0.27131	-0.469492	3.1527
O	0.391049	0.713359	3.779127
C	-0.123307	-1.582192	4.091734
C	1.47225	2.684295	-3.883607
C	1.654093	1.873623	-2.592228
C	-3.420995	4.249538	-1.926712
C	-2.862512	4.327206	-0.507642
O	-3.495517	4.844527	0.431148
N	-1.624605	3.756958	-0.348681

C	-1.118775	3.445399	0.993737
C	-1.892036	2.290933	1.659715
C	2.50521	2.159819	0.854864
C	3.142305	2.618589	2.169593
O	2.696783	1.749118	3.236074
C	2.557788	-1.335064	-0.591233
C	3.890371	-1.401658	-1.376183
O	4.040986	-0.844466	-2.475395
C	1.473696	-1.890225	-1.522156
N	4.808974	-2.249964	-0.814283
C	6.184866	-2.392856	-1.297324
C	6.583589	-3.87685	-1.291402
C	7.123888	-1.510709	-0.437971
O	6.838891	-0.127469	-0.532833
C	-4.613487	-2.377017	1.49628
N	-3.299072	-2.975545	1.348235
C	-3.105953	-4.294445	1.205853
N	-4.156855	-5.147786	1.401975
N	-1.918131	-4.749645	0.779312
C	-7.013977	-1.978609	-2.179931
N	-5.608269	-1.856533	-2.536425
C	-4.783505	-0.941532	-1.981045
N	-5.225375	-0.065753	-1.058461
N	-3.48355	-0.890775	-2.339836
H	2.455045	3.050406	-4.234726
H	0.817435	3.559292	-3.721814
H	1.029487	2.07134	-4.689798
H	2.345504	1.026139	-2.739992
H	2.086619	2.519573	-1.811648
H	-2.629979	4.206364	-2.694543
H	-4.072394	5.117998	-2.113727
H	-4.028665	3.330828	-2.014242
H	-1.271756	3.151456	-1.105924
H	-1.313875	4.342249	1.607543
H	-1.467581	2.043365	2.647527
H	-2.960044	2.551751	1.758128
H	2.823017	2.928308	0.118149
H	4.239449	2.537586	2.057131
H	2.853624	3.663126	2.370986
H	2.935219	2.170679	4.087157
H	2.625251	-1.994751	0.301223
H	1.475475	-1.339516	-2.477663
H	1.606497	-2.967929	-1.719556
H	7.083613	-1.881569	0.615803
H	8.165564	-1.648644	-0.786583
H	7.602016	-4.010419	-1.698613
H	5.884135	-4.470957	-1.903603
H	6.181434	-1.992708	-2.325946
H	4.646164	-2.509751	0.160927
H	6.577119	-4.288429	-0.26395
H	5.913372	0.026283	-0.203103
H	0.282004	-1.640235	1.604811
H	-1.675974	-5.731453	0.898516
H	-1.166033	-4.029971	0.530747

H	-3.966728	-6.148467	1.380825
H	-4.881518	-4.869189	2.062832
H	-2.505851	-2.315188	1.269794
H	-4.479132	-1.291945	1.402478
H	-5.055737	-2.584909	2.491411
H	-5.307355	-2.740530.716632	
H	-5.278418	-2.329647	-3.376253
H	-4.497402	0.553647	-0.636422
H	-6.207701	0.19619 -1.014419	
H	-2.896447	-0.210851	-1.804164
H	-3.025409	-1.724903	-2.704856
H	-7.393482	-2.93119-2.578159	
H	-7.630681	-1.15796-2.594876	
H	-7.125467	-1.996267	-1.082268
H	0.645861	-1.715371	4.87285
H	-0.273124	-2.531393	3.555105
H	-1.064799	-1.304841	4.599261
H	1.081487	1.275837	3.291475

Table S33. Optimized coordinates for free acetonitrile + 2 H_2O.

C	1.372344	-0.555851	0.032863
N	0.902322	-1.621283	0.115121
C	1.868316	0.806439	-0.077393
O	-1.997159	-0.950161	-0.170302
O	-1.180978	1.73122 -0.025367	
H	2.456115	0.929023	-1.002742
H	0.970461	1.46292 -0.105323	
H	2.500179	1.060174	0.790546
H	-2.338103	-1.166907	0.721972
H	-1.113221	-1.390766	-0.186184
H	-1.514321	0.793586	-0.121252
H	-1.296225	1.908956	0.929664

Table S34. Optimized coordinates for attack on free acetonitrile by 2 H_2O, transition state.

C	0.695618	-0.366784	-0.011524
N	-0.042525	-1.324226	-0.047763
C	2.121309	0.032541	0.040523
O	-2.186248	-0.077718	-0.076701
O	-0.174253	1.262867	0.082455
H	2.387734	0.640841	-0.841284
H	2.30942 0.643275	0.938644	
H	2.753152	-0.870512	0.060233
H	-2.590753	-0.131279	0.816908
H	-1.265382	-0.911433	-0.069865
H	-1.312615	0.751541	0.004855
H	-0.001428	1.671419	-0.795172

Table S35. Optimized coordinates for free acetamide tautomer + H_2O.

C	0.733482	-0.147337	-0.013016
N	0.169599	-1.288958	-0.04036
C	2.227946	0.040687	0.048068

O	-2.699488	-0.006962	-0.061345
O	-0.012331.031542	-0.036889	
H	2.515945	0.606672	0.953354
H	2.588196	0.606458	-0.831173
H	2.722067	-0.940727	0.067232
H	-2.854036	-0.078402	0.902934
H	-0.863493	-1.175035	-0.088333
H	-1.957307	0.638542	-0.114073
H	0.587417	1.808454	-0.031859

Table S36. Optimized coordinates for acetonitrile-bound NHase, no extra H_2O.

Fe	-0.27707-0.456499	0.22198	
S	0.023993	-1.292048	-1.842988
S	0.20263	1.665389	-0.637156
S	1.822444	-0.747139	0.782426
O	0.174085	2.776603	0.514634
O	2.387423	0.343549	1.694987
O	2.816913	-1.033213	-0.383244
N	-0.98652-2.126673	0.991781	
C	-0.399836	-3.358505	1.074786
O	-1.032388	-4.433324	1.134196
N	-2.156193	-0.075541	-0.136182
C	-3.053655	-0.837674	0.500486
O	-4.277566	-0.571553	0.663161
N	-0.563521	0.408102	1.923306
C	-0.841920.928375	2.925057	
C	-1.222391	1.529454	4.189917
C	7.01042	2.052295	-2.062499
N	5.607248	1.904505	-2.421678
C	4.798268	0.976311	-1.86544
N	5.254364	0.116215	-0.935562
N	3.501563	0.895428	-2.232351
C	4.560871	2.481482	1.57792
N	3.232857	3.0509	1.420626
C	3.020276	4.361956	1.230862
N	4.060207	5.23478	1.408131
N	1.833084	4.785215	0.775152
N	-4.807651	2.135949	-0.864802
C	-6.185343	2.230188	-1.35526
C	-6.630283.701188	-1.364474	
C	-7.101495	1.333024	-0.485435
O	-6.766497	-0.040628	-0.544216
C	-2.549383	1.266294	-0.574582
C	-1.440748	1.839303	-1.461404
C	-3.862106	1.295733	-1.394587
O	-3.979486	0.71188	-2.483946
C	-2.473094	-2.154861.047007	
C	-2.982716	-2.448439	2.47975
O	-2.304551	-1.669022	3.476976
N	1.685318	-3.780641	-0.193529
C	1.150078	-3.454385	1.13229
C	1.897682	-2.275424	1.788088
C	3.536583	-4.238464	-1.715801

C	2.945678	-4.313094	-0.312298
O	3.569805	-4.793296	0.651167
C	-1.363095	-2.810922	-3.759015
C	-1.575537	-1.992078	-2.47569
H	-2.333915	-3.207856	-4.108989
H	-0.685027	-3.665724	-3.586188
H	-0.935782	-2.192668	-4.569206
H	-2.287666	-1.163754	-2.634239
H	-1.987966	-2.635582	-1.680971
H	3.855072	6.231356	1.355246
H	4.776322	4.988828	2.091145
H	1.575626	5.766291	0.868383
H	1.087964	4.036885	0.559444
H	2.443042	2.385491	1.357591
H	4.447571	1.391271	1.519305
H	5.006174	2.729064	2.562352
H	5.241188	2.833422	0.7815
H	7.375703	3.006299	-2.4704
H	7.640479	1.237015	-2.467715
H	7.119474	2.083795	-0.964871
H	5.273097	2.364337	-3.267216
H	4.53982	-0.511286	-0.50467
H	6.242502	-0.12045	-0.880696
H	2.928904	0.20738	-1.694458
H	3.024965	1.722213	-2.590916
H	4.224709	-5.08442	-1.871472
H	4.11089	-3.298639	-1.804148
H	2.764978	-4.238567	-2.504398
H	1.336479	-3.196612	-0.968103
H	1.459368	-2.020052.767683	
H	2.96587	-2.523839	1.913339
H	1.35228	-4.335271.766621	
H	-2.858535	-2.979705	0.41178
H	-4.052854	-2.185006	2.528553
H	-2.86025	-3.529771	2.683044
H	-6.166949	1.818211	-2.378991
H	-7.654864	3.798362	-1.766415
H	-5.953487	4.310276	-1.987363
H	-6.629815	4.123417	-0.341396
H	-7.086315	1.728733	0.559689
H	-8.143146	1.426495	-0.848414
H	-2.660216	1.92768	0.312121
H	-1.383182	1.28015	-2.410741
H	-1.588967	2.912255	-1.673725
H	-5.853605	-0.15564	-0.166402
H	-4.665303	2.405808	0.110965
H	-1.363299	-1.696029	3.189596
H	-0.348989	1.613441	4.859416
H	-1.987788	0.89028	4.663397
H	-1.642537	2.535688	4.020945

Table S37. Optimized coordinates for attack by sulfenate on NHase-bound acetonitrile (no extra H_2O), transition state.

Fe	-0.294886	0.520925	-0.290048
S	0.015572	1.406794	1.78277
S	0.123442	-1.596286	0.661879
S	1.816533	0.735064	-0.835979
O	0.025936	-2.691736	-0.575966
O	2.375973	-0.418922	-1.677057
O	2.79779 1.048679	0.335223	
N	-0.939406	2.136528	-1.089805
C	-0.326506	3.341222	-1.30189
O	-0.947397	4.399163	-1.535113
N	-2.183521	0.187723	0.087109
C	-3.064256	0.956002	-0.567606
O	-4.303757	0.750655	-0.676644
N	-0.566342	-0.560307	-1.875259
C	-0.505737	-1.73803-2.105792	
C	-0.646116	-2.813181	-3.107425
C	6.953205	-1.958015	2.206278
N	5.550177	-1.775095	2.550875
C	4.753364	-0.873261.934699	
N	5.218618	-0.089343	0.943927
N	3.461259	-0.745404	2.300634
C	4.561046	-2.495881	-1.457742
N	3.228911	-3.049325	-1.28761
C	3.000065	-4.351536	-1.071814
N	4.040274	-5.233757	-1.13289
N	1.773097	-4.767973	-0.714681
N	-4.879997	-1.957738	0.836017
C	-6.253306	-2.055389	1.333769
C	-6.686597	-3.529524	1.336164
C	-7.1698 -1.152626	0.470351	
O	-6.816131	0.216924	0.524222
C	-2.613908	-1.131605	0.546115
C	-1.532824	-1.740401	1.442318
C	-3.925333	-1.1324 1.369302	
O	-4.027406	-0.555151	2.463165
C	-2.418658	2.16833 -1.258517	
C	-2.809236	2.187983	-2.754716
O	-2.425465	1.007506	-3.463406
N	1.726924	3.784589	0.030097
C	1.223773	3.42335 -1.299476	
C	1.958674	2.208664	-1.904912
C	3.535875	4.342351	1.582633
C	2.979995	4.332486	0.160704
O	3.628422	4.770701	-0.806896
C	-1.408395	3.023039	3.591418
C	-1.596894	2.135151	2.352583
H	-2.384739	3.443284	3.896354
H	-0.721583.863903	3.387208	
H	-1.003509	2.450022	4.445427
H	-2.312579	1.318527	2.546597
H	-1.996012.733902	1.516823	
H	3.828011	-6.228901	-1.080724
H	4.830806	-5.001033	-1.733123
H	1.536908	-5.757495	-0.738617

H	1.014158	-4.049083	-0.588766
H	2.460325	-2.354871	-1.291235
H	4.448302	-1.404574	-1.473095
H	5.021623	-2.811343	-2.415075
H	5.225649	-2.79183-0.626276	
H	7.314945	-2.872852.698065	
H	7.586568	-1.112398	2.537314
H	7.063294	-2.088608	1.116317
H	5.217524	-2.159703	3.434058
H	4.511708	0.523578	0.479398
H	6.209846	0.130125	0.8728
H	2.89255	-0.085818	1.722737
H	2.982942	-1.523132	2.752778
H	4.162007	5.237735	1.724257
H	4.172803	3.449521	1.717336
H	2.746176	4.316362	2.352439
H	1.354694	3.228085	0.815543
H	1.524886	1.928285	-2.879702
H	3.034736	2.42542	-2.019293
H	1.456665	4.279971	-1.954961
H	-2.824196	3.097433	-0.811073
H	-3.910126	2.264025	-2.817689
H	-2.368459	3.098166	-3.210896
H	-6.230751	-1.648947	2.359658
H	-7.710488	-3.638	1.737222
H	-6.004742	-4.135425	1.956627
H	-6.682442	-3.946434	0.311031
H	-7.168104	-1.550007	-0.573988
H	-8.208981	-1.232587	0.84304
H	-2.748391	-1.797509	-0.334426
H	-1.46233-1.183188	2.39273	
H	-1.717756	-2.806565	1.657278
H	-5.903576	0.32026	0.143135
H	-4.745063	-2.220467	-0.142773
H	-1.596489	0.670248	-3.043396
H	0.324322	-3.311279	-3.276182
H	-0.99914-2.379953	-4.05767	
H	-1.367966	-3.572248	-2.76243

Table S38. Optimized coordinates for attack by sulfenate on NHase-bound acetonitrile (no extra H_2O), cyclic intermediate.

Fe	0.294658	-0.507751	-0.336971
S	-0.066982	-1.318774	1.881038
S	-0.084817	1.240015	0.934614
S	-1.807421	-0.69847-0.908822	
O	0.015884	2.581289	-0.414184
O	-2.367261	0.451235	-1.756703
O	-2.783202	-0.985946	0.279247
N	0.902005	-2.117593	-1.168424
C	0.287368	-3.322402	-1.379679
O	0.896861	-4.376099	-1.657488
N	2.194602	-0.250303	0.072534
C	3.056407	-0.997737	-0.634833

O	4.300557	-0.824443	-0.728318
N	0.568247	0.702138	-1.750034
C	0.411211	1.951796	-1.62188
C	0.599796	2.974946	-2.721886
C	-6.935872	1.984011	2.191263
N	-5.538456	1.77835	2.544582
C	-4.744403	0.883912	1.914261
N	-5.205636	0.130517	0.898623
N	-3.458580.731523	2.29201	
C	-4.553117	2.498228	-1.482255
N	-3.220483	3.039639	-1.278777
C	-2.978258	4.344101	-1.105632
N	-3.989055.243566	-1.253876	
N	-1.764055	4.754876	-0.682822
N	4.92257 1.862767	0.792885	
C	6.304997	1.978896	1.258681
C	6.71113 3.461511	1.270058	
C	7.211652	1.096705	0.364332
O	6.867242	-0.276523	0.408913
C	2.647676	1.045032	0.582079
C	1.607718	1.599091	1.558355
C	3.989774	1.032711	1.354065
O	4.129295	0.45507	2.444097
C	2.368166	-2.128247	-1.415564
C	2.671742	-1.963078	-2.926943
O	2.368952	-0.673749	-3.451824
N	-1.7618 -3.739017	0.006661	
C	-1.26443-3.399467	-1.332654	
C	-1.991374	-2.186075	-1.949822
C	-3.563397	-4.340531	1.568774
C	-3.000118	-4.321890.147014	
O	-3.631928	-4.796061	-0.813868
C	1.396631	-3.050554	3.55743
C	1.566011	-2.046891	2.407784
H	2.384505	-3.475833.814407	
H	0.728836	-3.883609	3.275079
H	0.984871	-2.571007	4.463904
H	2.265248	-1.236991	2.675158
H	1.980864	-2.549856	1.519881
H	-3.775134	6.235946	-1.171708
H	-4.756912	5.014473	-1.883661
H	-1.470453	5.717183	-0.84132
H	-1.028751	4.035927	-0.518754
H	-2.466648	2.326447	-1.289055
H	-4.454953	1.406145	-1.467478
H	-4.974877	2.793549	-2.463485
H	-5.240941	2.827606	-0.683312
H	-7.295107	2.88644 2.707024	
H	-7.579739	1.134236	2.489858
H	-7.033862.148804	1.104741	
H	-5.209976	2.141005	3.438547
H	-4.499409	-0.479070.427632	
H	-6.199005	-0.069506	0.804704
H	-2.887309	0.092851	1.693123

H	-2.981857	1.482224	2.788813
H	-4.004729	-5.330214	1.771223
H	-4.371089	-3.590283	1.636626
H	-2.808936	-4.109483	2.339429
H	-1.411531	-3.160542	0.781694
H	-1.568261	-1.931762	-2.936306
H	-3.073252	-2.383571	-2.042227
H	-1.517238	-4.264242	-1.96918
H	2.791408	-3.102636	-1.104343
H	3.755285	-2.133644	-3.075138
H	2.124116	-2.766872	-3.46606
H	6.31307 1.561832		2.280491
H	7.741083	3.583692	1.65122
H	6.031429	4.046039	1.91289
H	6.678109	3.891403	0.250885
H	7.185094	1.506498	-0.674592
H	8.257693	1.178439	0.716398
H	2.741453	1.750124	-0.270946
H	1.689306	1.103867	2.539893
H	1.685836	2.690135	1.69357
H	5.946435	-0.379975	0.049833
H	4.755944	2.131729	-0.179223
H	1.604698	-0.285261	-2.939309
H	-0.355465	3.474961	-2.967368
H	0.97299 2.46688 -3.623225		
H	1.320969	3.755269	-2.418544

Table S39. Optimized coordinates for attack by water on the cyclic intermediate S, transition state.

Fe	0.289081	0.490409	0.292671
S	-0.057301	1.59807 -1.768238	
S	-0.057415	-0.694162	-1.640484
S	-1.788475	0.656223	0.939033
O	0.361793	-3.025366	1.146797
O	-2.340337	-0.473312	1.810267
O	-2.772684	0.955364	-0.246484
N	0.86334 2.095073		1.195208
C	0.261864	3.30339 1.391996	
O	0.881014	4.350448	1.681123
N	2.184599	0.334387	-0.17041
C	3.002503	0.931205	0.710779
O	4.218876	0.674141	0.909077
N	0.616989	-0.727911	1.634616
C	0.653762	-1.971181	1.944978
C	1.055883	-2.398668	3.353199
C	-6.942837	-2.019566	-2.071931
N	-5.555655	-1.801581	-2.442608
C	-4.763069	-0.890386	-1.83914
N	-5.227394	-0.080703	-0.872155
N	-3.467167	-0.783687	-2.209259
C	-4.46878-2.517662		1.543055
N	-3.129892	-3.041949	1.327291
C	-2.876816	-4.338239	1.119987
N	-3.902689	-5.192829	0.814658

N	-1.628409	-4.826067	1.225865
N	4.892951	-1.756327	-0.978569
C	6.330427	-1.833412	-1.26672
C	6.741195	-3.302007	-1.474523
C	7.124521	-1.147078	-0.126655
O	6.846433	0.234404	0.001595
C	2.623199	-0.852009	-0.904514
C	1.702602	-1.024238	-2.120641
C	4.066549	-0.780151	-1.468259
O	4.369285	0.00541	-2.382135
C	2.309485	2.050063	1.511711
C	2.529933	1.8764	3.039095
O	1.648723	0.934885	3.647631
N	-1.829756	3.758851	0.045299
C	-1.292995	3.384081	1.361953
C	-1.980152	2.148264	1.975852
C	-3.649756	4.34996	-1.493853
C	-3.071706	4.34503	-0.072834
O	-3.687326	4.819941	0.895661
C	1.281079	3.143009	-3.603813
C	1.523771	2.392944	-2.299153
H	2.202088	3.680265	-3.897428
H	0.473614	3.889554	-3.505786
H	1.014	2.452714	-4.423339
H	2.333088	1.649424	-2.375755
H	1.788991	3.070999	-1.469285
H	-3.768762	-6.175481.052165	
H	-4.85468-4.860578	0.960707	
H	-1.426461	-5.722959	0.785194
H	-0.817551	-4.152769	1.342143
H	-2.376312	-2.334732	1.417583
H	-4.349994	-1.458446	1.804792
H	-4.978515	-3.041786	2.374191
H	-5.09393-2.586328	0.63292	
H	-7.284664	-2.95783-2.533195	
H	-7.608471	-1.203986	-2.415086
H	-7.034138	-2.122282	-0.976897
H	-5.21068-2.218777	-3.305593	
H	-4.514470.509909	-0.389087	
H	-6.219664	0.127132	-0.785209
H	-2.903881	-0.144067	-1.602436
H	-2.992627	-1.61381-2.565132	
H	-4.302204	5.228551	-1.615476
H	-4.260837	3.439472	-1.631422
H	-2.868997	4.357912	-2.27352
H	-1.523749	3.184457	-0.745652
H	-1.528893	1.901412	2.95152
H	-3.066113	2.309434	2.089016
H	-1.534235	4.232186	2.025039
H	2.794123	3.005214	1.22784
H	3.596529	1.594359	3.179722
H	2.368662	2.85545	3.529272
H	6.480043	-1.250437	-2.191878
H	7.813325	-3.372358	-1.730625

H	6.158072	-3.760474	-2.291175
H	6.575228	-3.896136	-0.555829
H	6.933513	-1.707833	0.820257
H	8.206506	-1.239273	-0.341963
H	2.522688	-1.744945	-0.251673
H	1.977967	-0.317504	-2.919492
H	1.739888	-2.039543	-2.544878
H	5.893686	0.336052	0.265807
H	4.585897	-2.212349	-0.116273
H	1.271908	0.352038	2.9191
H	0.1805 -2.819878	3.883091	
H	1.450868	-1.548718	3.930791
H	1.821914	-3.193405	3.297065
O	-0.365175	-2.83942-1.166237	
H	0.029081	-2.849188	-0.075676
H	0.275368	-3.374634	-1.684205

Table S40. Optimized coordinates for attack by water on the cyclic intermediate S, amidate tautomer + sulfenic acid (SOH).

Fe	0.282589	0.475815	0.271062
S	-0.078675	1.618246	-1.753781
S	-0.095289	-0.849361	-1.581433
S	-1.790970.637343	0.929768	
O	0.563798	-3.080413	1.301265
O	-2.312526	-0.509016	1.804291
O	-2.800728	0.936951	-0.226996
N	0.860756	2.082843	1.173513
C	0.25454 3.288364	1.375989	
O	0.873125	4.339847	1.6494
N	2.175675	0.312485	-0.183029
C	3.000915	0.929861	0.674998
O	4.222385	0.684626	0.861269
N	0.62088 -0.725792	1.643696	
C	0.755944	-1.912204	2.062002
C	1.139815	-2.285711	3.480299
C	-6.951912	-1.998703	-2.090405
N	-5.56254-1.77958-2.463135		
C	-4.769562	-0.870603	-1.849713
N	-5.222811	-0.119702	-0.831968
N	-3.494606	-0.705794	-2.261432
C	-4.478788	-2.518506	1.523116
N	-3.140846	-3.041886	1.299645
C	-2.887201	-4.335612	1.089404
N	-3.902074	-5.197649	0.782252
N	-1.634405	-4.821779	1.191959
N	4.888147	-1.746614	-1.001757
C	6.321223	-1.815672	-1.305269
C	6.732274	-3.284976	-1.493002
C	7.124984	-1.108775	-0.185108
O	6.838308	0.271887	-0.071688
C	2.611627	-0.874327	-0.913322
C	1.667382	-1.070074	-2.105935
C	4.044266	-0.790311	-1.500418

O	4.326852	-0.017144	-2.430179
C	2.312785	2.059152	1.463243
C	2.562521	1.925424	2.988964
O	1.700867	0.984656	3.631138
N	-1.845905	3.742693	0.064158
C	-1.299378	3.360895	1.372628
C	-1.972699	2.116316	1.98556
C	-3.658096	4.367369	-1.47246
C	-3.099918	4.294614	-0.04598
O	-3.747664	4.701435	0.934706
C	1.273688	3.172336	-3.586285
C	1.492596	2.445723	-2.260689
H	2.19774 3.705945	-3.87765	
H	0.461017	3.91702 -3.516438	
H	1.020165	2.464714	-4.395832
H	2.310613	1.709986	-2.324156
H	1.747872	3.148026	-1.448199
H	-3.771511	-6.178811.026989	
H	-4.859352	-4.865240.884663	
H	-1.41611-5.708294	0.739491	
H	-0.834087	-4.175631	1.369606
H	-2.395228	-2.320625	1.377115
H	-4.356358	-1.468385	1.817719
H	-4.99348-3.066414	2.335165	
H	-5.099237	-2.555314	0.608645
H	-7.311382	-2.902081	-2.604471
H	-7.608383	-1.153785	-2.375442
H	-7.033658	-2.168535	-1.003089
H	-5.24715-2.131131	-3.366295	
H	-4.510471	0.47206 -0.347276	
H	-6.217507	0.046212	-0.697705
H	-2.917848	-0.086245	-1.646659
H	-3.020314	-1.48173-2.721644	
H	-4.169868	5.333582	-1.610842
H	-4.406753	3.565188	-1.600363
H	-2.883809	4.246168	-2.248663
H	-1.481808	3.22357 -0.744666	
H	-1.506714	1.864429	2.952968
H	-3.057134	2.274758	2.114229
H	-1.535599	4.199358	2.050219
H	2.782078	3.011888	1.146994
H	3.63213 1.653345	3.12046	
H	2.398828	2.913464	3.45945
H	6.45661 -1.246459	-2.241124	
H	7.80223 -3.360439	-1.757326	
H	6.142188	-3.757481	-2.296569
H	6.574756	-3.864122	-0.563211
H	6.949772	-1.658377	0.771599
H	8.204861	-1.196647	-0.412674
H	2.541754	-1.763471	-0.25014
H	1.882111	-0.324003	-2.886571
H	1.762063	-2.07173-2.557788	
H	5.886745	0.369019	0.199643
H	4.597487	-2.188203	-0.12653

H	1.279854	0.423365	2.915084
H	0.268033	-2.711853	4.01191
H	1.496734	-1.399006	4.025195
H	1.931504	-3.056116	3.47003
O	-0.324862	-2.692801	-1.080111
H	0.337888	-2.845126	0.330586
H	0.086001	-3.22994-1.794708	

Table S41. Optimized coordinates for acetonitrile-coordinated NHase, no oxidized thiolate ligands, S=1/2.

Fe	-0.039153	-0.355014	0.120693
S	0.19421 -1.606008		-1.790721
S	0.259683	1.71155 -0.994614	
S	2.239753	-0.413408	0.639673
N	-0.628572	-1.916291	1.187576
C	0.042908	-3.063243	1.477283
O	-0.510516	-4.149317	1.770949
N	-1.933287	-0.110949	-0.156785
C	-2.758884	-0.813191	0.635033
O	-3.982828	-0.562836	0.831967
N	-0.233281	0.7047 1.753983	
C	-0.529154	1.303186	2.709398
O	-1.154514	3.991647	1.093086
C	-0.971988	2.04631 3.87606	
C	6.803401	1.996322	-2.25388
N	5.412224	1.714108	-2.576364
C	4.669608	0.803719	-1.917251
N	5.201554	0.04506 -0.938366	
N	3.368124	0.636221	-2.236349
C	4.402997	2.562516	1.399943
N	3.093964	3.018933	0.985649
C	2.738002	4.308847	0.943831
N	3.538012	5.264174	1.472328
N	1.614187	4.657152	0.271985
N	-4.817658	1.618692	-0.978012
C	-6.216839	1.463718	-1.385252
C	-6.898912.837725		-1.352316
C	-6.935737	0.438867	-0.470279
O	-6.375727	-0.859341	-0.514346
C	-2.438366	1.111431	-0.797446
C	-1.398498	1.629891	-1.801096
C	-3.781315	0.919973	-1.548772
O	-3.861660.301342		-2.620859
C	-2.099867	-2.018498	1.315539
C	-2.54242-2.144116		2.795759
O	-1.848462	-1.219472	3.648526
N	2.100117	-3.525399	0.20824
C	1.593999	-3.042221	1.491107
C	2.272265	-1.725667	1.929627
C	3.749816	-4.451409	-1.325193
C	3.309126	-4.150784	0.104394
O	4.005209	-4.472433	1.087703
C	-1.270739	-3.502938	-3.353455

C	-1.328465	-2.648485	-2.069065
H	-2.211956	-4.073771	-3.457215
H	-0.432771	-4.222825	-3.326088
H	-1.151547	-2.869471	-4.2507
H	-2.187043	-1.956324	-2.109528
H	-1.455939	-3.302817	-1.189398
H	3.26263	6.241046	1.385628
H	4.177339	5.034218	2.231131
H	1.019375	5.394876	0.653857
H	1.074777	3.877976	-0.148769
H	2.395996	2.287307	0.783268
H	4.473379	1.492992	1.158075
H	4.560875	2.673406	2.490672
H	5.195561	3.11526	0.865093
H	7.084841	2.94794	-2.727849
H	7.489389	1.209262	-2.620488
H	6.924071	2.103771	-1.162913
H	5.026387	2.088905	-3.442245
H	4.545707	-0.527154	-0.383092
H	6.194771	-0.177422	-0.915016
H	2.82329	-0.017915	-1.651664
H	2.814736	1.418077	-2.59514
H	3.842915	-5.544259	-1.455081
H	4.751762	-4.019284	-1.493636
H	3.056284	-4.061959	-2.089714
H	1.644482	-3.126385	-0.630679
H	1.78132	-1.346298	2.844653
H	3.327626	-1.943636	2.170139
H	1.875248	-3.807177	2.237777
H	-2.473603	-2.931358	0.80324
H	-3.619204	-1.9125	2.860384
H	-2.364032	-3.184403	3.126964
H	-6.189368	1.056881	-2.410769
H	-7.951354	2.765946	-1.682159
H	-6.377293	3.550706	-2.013318
H	-6.897648	3.255374	-0.327198
H	-6.957786	0.852198	0.567264
H	-7.988714	0.351615	-0.802317
H	-2.585787	1.886589	-0.010699
H	-1.344327	0.927764	-2.649776
H	-1.684161	2.628125	-2.177414
H	-5.481214	-0.803332	-0.079746
H	-4.679267	1.837304	0.01192
H	-0.932365	-1.222945	3.283091
H	-2.019599	4.160384	0.666592
H	-0.814163	3.196161	0.600471
H	-0.134869	2.229214	4.571742
H	-1.75114	1.458384	4.391458
H	-1.383316	3.012545	3.534547

Table S42. Optimized coordinates for acetonitrile-coordinated NHase, no oxidized thiolate ligands, S=3/2.

Fe	-0.07634	-0.558491	0.015814

S	0.212518	-2.230399	-1.731023
S	0.117575	1.422032	-1.167227
S	2.070648	-0.286582	0.685643
N	-0.555786	-1.657594	1.540185
C	0.106237	-2.738887	2.068549
O	-0.458827	-3.654526	2.698512
N	-1.956329	-0.184493	-0.055259
C	-2.716545	-0.635238	0.967866
O	-3.91043	-0.301685	1.184026
N	1.70891	3.801669	3.514625
C	0.563958	3.572976	3.509583
O	-0.874679	4.225055	0.168923
C	-0.865249	3.309246	3.453344
C	6.564554	1.452411	-2.822567
N	5.225662	0.963361	-3.121685
C	4.465939	0.293759	-2.235727
N	4.971244	-0.126438	-1.057972
N	3.168403	0.051369	-2.510295
C	4.16382	2.670702	0.668954
N	3.130556	3.10008	-0.266461
C	2.448727	4.245759	-0.110334
N	2.671273	5.0394	0.962018
N	1.586234	4.675213	-1.076557
N	-4.9599	1.19466	-1.246845
C	-6.348766	0.839064	-1.574963
C	-7.152298	2.090724	-1.976129
C	-6.999955	0.10985	-0.370718
O	-6.33642	-1.082198	0.006391
C	-2.551271	0.85522	-0.921255
C	-1.521116	1.21411	-1.997962
C	-3.878812	0.420092	-1.604435
O	-3.915737	-0.470928	-2.466455
C	-1.984708	-1.581789	1.916327
C	-2.102369	-1.067117	3.374297
O	-1.256085	0.076024	3.583828
N	2.089354	-3.504886	0.756517
C	1.649889	-2.788315	1.953179
C	2.33863	-1.420771	2.112778
C	3.696447	-4.760595	-0.601284
C	3.302845	-4.138563	0.736587
O	4.041674	-4.222718	1.73722
C	-1.356788	-4.368967	-2.729831
C	-1.38219	-3.195698	-1.739645
H	-2.322577	-4.908917	-2.699847
H	-0.553478	-5.085839	-2.48226
H	-1.193016	-4.015437	-3.763564
H	-2.197803	-2.494669	-1.985114
H	-1.55354	-3.569779	-0.714978
H	2.164798	5.925994	0.973184
H	2.766774	4.595939	1.88837
H	0.630999	4.881721	-0.703511
H	1.524422	4.050191	-1.886229
H	2.649477	2.37133	-0.806602
H	4.780606	1.902389	0.184374

H	3.72823	2.23949	1.587451
H	4.795763	3.536729	0.925938
H	6.818705	2.237534	-3.549477
H	7.327984	0.655242	-2.891317
H	6.58958	1.892426	-1.812188
H	4.87238	1.052336	-4.073538
H	4.297306	-0.49394-0.36536	
H	5.958858	-0.356613	-0.9585
H	2.598719	-0.473409	-1.833574
H	2.650679	0.617989	-3.18137
H	3.786977	-5.854184	-0.477517
H	4.691017	-4.383366	-0.895882
H	2.974472	-4.553144	-1.409297
H	1.573604	-3.317731	-0.123526
H	1.987539	-0.930201	3.03809
H	3.424929	-1.588651	2.210456
H	1.971112	-3.407088	2.810355
H	-2.464498	-2.579034	1.869437
H	-3.140943	-0.752914	3.570018
H	-1.827828	-1.886724.066398	
H	-6.279782	0.132319	-2.41985
H	-8.184257	1.814139	-2.256818
H	-6.683867	2.600644	-2.835123
H	-7.211833	2.81061	-1.137957
H	-7.076965	0.833148	0.47673
H	-8.034497	-0.169512	-0.648165
H	-2.751081.753247	-0.296882	
H	-1.452527	0.387868	-2.725232
H	-1.802555	2.140516	-2.52922
H	-5.459142	-0.822957	0.394502
H	-4.839961	1.71494	-0.373844
H	-0.425342	-0.161452	3.105517
H	-1.722834.395022	-0.291569	
H	-0.642209	3.282308	-0.091865
H	-1.384493	3.859732	4.256615
H	-1.070529	2.224542	3.55852
H	-1.235567	3.650752	2.467643

Table S43. Optimized coordinates for acetonitrile-coordinated NHase, no oxidized thiolate ligands, S=5/2.

Fe	-0.027363	-0.67459-0.08075	
S	0.273343	-2.236533	-1.799289
S	0.094559	1.572757	-1.154267
S	2.193792	-0.344259	0.757536
N	-0.577551	-1.739946	1.550914
C	0.043299	-2.858407	2.047179
O	-0.53628-3.794488	2.629253	
N	-1.995188	-0.187767	-0.06187
C	-2.724591	-0.615725	0.98715
O	-3.904134	-0.259601	1.256678
N	1.71609	3.803239	3.565593
C	0.575743	3.552251	3.557674
O	-0.913772	4.312232	0.26481

C	-0.847577	3.261052	3.497888
C	6.552592	1.557744	-2.802358
N	5.219159	1.059573	-3.112993
C	4.462772	0.37386 -2.235963	
N	4.96452 -0.064083		-1.066388
N	3.164927	0.129276	-2.516612
C	4.129173	2.715649	0.693826
N	3.100607	3.150885	-0.24463
C	2.402877	4.284227	-0.073543
N	2.623444	5.067073	1.008988
N	1.534205	4.71926 -1.030563	
N	-4.978096	1.195746	-1.236422
C	-6.362059	0.827084	-1.568347
C	-7.172175	2.065149	-1.997371
C	-7.008085	0.11186 -0.35317	
O	-6.319407	-1.056308	0.053341
C	-2.571156	0.867599	-0.89937
C	-1.538335	1.269201	-1.970304
C	-3.888609	0.441258	-1.606359
O	-3.914331	-0.436184	-2.483594
C	-1.998346	-1.590742	1.929029
C	-2.073208	-1.072739	3.388291
O	-1.180914	0.037014	3.581776
N	2.051756	-3.603082	0.741866
C	1.595145	-2.907952	1.946317
C	2.314968	-1.556659	2.153514
C	3.732303	-4.704831	-0.66051
C	3.288312	-4.194445	0.708806
O	4.010357	-4.313144	1.716617
C	-1.316878	-4.334252	-2.802414
C	-1.36288-3.130684		-1.855683
H	-2.301441	-4.838975	-2.812729
H	-0.556957	-5.068782	-2.481915
H	-1.080578	-4.023092	-3.835584
H	-2.132885	-2.402507	-2.162349
H	-1.598149	-3.459913	-0.829204
H	2.097188	5.941729	1.038098
H	2.726503	4.611181	1.928689
H	0.589349	4.942539	-0.640956
H	1.437192	4.077591	-1.825048
H	2.648903	2.437442	-0.82664
H	4.764484	1.969009	0.199676
H	3.689835	2.255677	1.596297
H	4.744583	3.585423	0.977172
H	6.805016	2.348593	-3.523628
H	7.322814	0.76709 -2.870166	
H	6.567735	1.993689	-1.789968
H	4.870632	1.151886	-4.066332
H	4.288968	-0.406968	-0.353549
H	5.959655	-0.246234	-0.947608
H	2.605167	-0.419505	-1.85335
H	2.639972	0.711924	-3.168114
H	3.963883	-5.781606	-0.588578
H	4.665102	-4.18915-0.948669	

H	2.978345	-4.551477	-1.451
H	1.565168	-3.386876	-0.143501
H	1.933361	-1.070237	3.068411
H	3.385693	-1.770417	2.306476
H	1.902898	-3.546093	2.794498
H	-2.518968	-2.568256	1.89111
H	-3.095132	-0.722839	3.606866
H	-1.814245	-1.903873	4.074111
H	-6.282385	0.106507	-2.400638
H	-8.201699	1.77637	-2.275095
H	-6.704372	2.55939	-2.865818
H	-7.238234	2.802501	-1.175113
H	-7.106786	0.851387	0.477783
H	-8.03412-0.196529	-0.631188	
H	-2.781153	1.752516	-0.259277
H	-1.440395	0.45296	-2.705967
H	-1.867141	2.180832	-2.498572
H	-5.452127	-0.770513	0.44729
H	-4.861778	1.710485	-0.359504
H	-0.375733	-0.223763.07198	
H	-1.766264.492589	-0.183961	
H	-0.684417	3.375948	-0.025713
H	-1.373559	3.767415	4.32549
H	-1.027777	2.168181	3.561108
H	-1.230815	3.63427	2.528584

Table S44. Optimized coordinates for five-coordinate NHase, S=1/2.

Fe	0.269568	-0.578427	-0.178528
S	-0.118865	-1.372139	1.812896
S	-0.133176	1.559416	0.530439
S	-1.800757	-0.72104-0.857673	
O	-0.065163	2.557561	-0.739848
O	-2.189734	0.431856	-1.784869
O	-2.877772	-0.940765	0.244461
N	0.861422	-2.117443	-1.157891
C	0.259939	-3.356867	-1.254228
O	0.887098	-4.421274	-1.388938
N	2.090041	-0.105366	-0.137738
C	2.965096	-0.889293	-0.820954
O	4.175654	-0.635191	-1.009339
O	2.21987	3.587642	-1.853724
C	-6.874775	2.291274	1.957386
N	-5.478924	2.076651	2.315393
C	-4.719199	1.106668	1.757385
N	-5.218591	0.294593	0.809554
N	-3.439598	0.935447	2.154106
C	-4.390258	2.634801	-1.668446
N	-3.038918	3.134608	-1.480074
C	-2.758094.430811	-1.294648	
N	-3.740675	5.365163	-1.459683
N	-1.540983	4.784654	-0.839737
N	4.903205	1.852539	0.744537
C	6.284467	1.821955	1.236435

C	6.834945	3.253726	1.331279
C	7.145923	0.921201	0.315186
O	6.710703	-0.426385	0.273253
C	2.592527	1.172978	0.402865
C	1.516293	1.746236	1.326925
C	3.903896	1.040185	1.212614
O	3.983369	0.351599	2.24359
C	2.321643	-2.14592	-1.402536
C	2.603157	-2.220981	-2.924317
O	1.9688	-1.15833	-3.638947
N	-1.831894	-3.758808	0.055111
C	-1.29007	-3.431093	-1.268681
C	-1.983399	-2.21174	-1.901741
C	-3.722643	-4.163483	1.555511
C	-3.116065	-4.244812	0.15824
O	-3.745617	-4.688298	-0.817501
C	1.233844	-3.008262	3.633527
C	1.440375	-2.253389	2.311239
H	2.171264	-3.522198	3.914468
H	0.438083	-3.769227	3.545352
H	0.964313	-2.319297	4.45396
H	2.249001	-1.505368	2.388479
H	1.700032	-2.955627	1.50085
H	-3.478555	6.347127	-1.386036
H	-4.478382	5.17142	-2.136517
H	-1.194295	5.722638	-1.036939
H	-0.844699	3.988418	-0.709898
H	-2.272623	2.435959	-1.454347
H	-4.341803	1.540772	-1.59639
H	-4.795249	2.896171	-2.666127
H	-5.068838	3.031846	-0.892521
H	-7.206601	3.23815	2.407619
H	-7.536057	1.482167	2.32327
H	-6.978334	2.378171	0.862261
H	-5.139035	2.488128	3.183489
H	-4.541825	-0.359291	0.358329
H	-6.221133	0.150494	0.714069
H	-2.903937	0.229239	1.603985
H	-2.919681	1.729351	2.52713
H	-4.417357	-5.005271	1.703845
H	-4.294838	-3.221506	1.632966
H	-2.961919	-4.165648	2.354578
H	-1.472556	-3.197022	0.840075
H	-1.54641	-1.964988	-2.883295
H	-3.066701	-2.391243	-2.007952
H	-1.52211	-4.296262	-1.913654
H	2.781604	-3.036579	-0.930849
H	3.688509	-2.110849	-3.086711
H	2.280223	-3.215077	-3.294898
H	6.235725	1.353286	2.234451
H	7.865613	3.249578	1.728781
H	6.209126	3.871585	1.997523
H	6.860552	3.736982	0.336085
H	7.178954	1.385069	-0.699727

H	8.18389	0.910254	0.699177
H	2.74561	1.874811	-0.442434
H	1.471004	1.176574	2.271683
H	1.679156	2.814562	1.550885
H	5.808594	-0.440513	-0.137679
H	4.767486	2.233622	-0.195095
H	1.032428	-1.175353	-3.339558
H	2.353405	4.225581	-1.123869
H	1.395479	3.103091	-1.556062

Table S45. Optimized coordinates for five-coordinate NHase, S=3/2.

Fe	0.254363	-0.569064	-0.187056
S	-0.21406	-1.390128	1.958666
S	-0.080813	1.643903	0.423356
S	-1.832101	-0.651761	-1.066371
O	0.026538	2.599276	-0.876011
O	-2.159881	0.451546	-2.067121
O	-2.872543	-0.765115	0.073319
N	0.819428	-2.145025	-1.159222
C	0.184209	-3.359202	-1.279501
O	0.781734	-4.437357	-1.451674
N	2.094748	-0.147579	-0.122077
C	2.954654	-0.975601	-0.773401
O	4.180174	-0.773513	-0.913442
O	2.377032	3.473505	-2.00124
C	-6.853798	2.304895	2.017652
N	-5.47451	2.046868	2.418533
C	-4.702904	1.111173	1.821785
N	-5.216729	0.323816	0.859514
N	-3.409643	0.955054	2.175022
C	-4.367287	2.627161	-1.608855
N	-3.009285	3.129	-1.479021
C	-2.728136	4.417834	-1.240289
N	-3.738455	5.339264	-1.279257
N	-1.48913	4.773284	-0.864717
N	4.938233	1.811467	0.734542
C	6.303325	1.782014	1.272762
C	6.859691	3.211206	1.391081
C	7.18652	0.873288	0.38153
O	6.746833	-0.473462	0.339527
C	2.626465	1.145052	0.354598
C	1.560325	1.790149	1.239039
C	3.920177	1.018598	1.197359
O	3.965718	0.362944	2.250409
C	2.284386	-2.209522	-1.376756
C	2.601758	-2.282243	-2.890642
O	2.018556	-1.194734	-3.613849
N	-1.906458	-3.649606	0.069928
C	-1.369052	-3.401318	-1.270716
C	-2.047628	-2.214404	-1.985232
C	-3.727116	-4.163485	1.63679
C	-3.168187	-4.174103	0.214369
O	-3.821106	-4.621325	-0.746111

C	1.233519	-3.021263.712059	
C	1.381725	-2.217674	2.419545
H	2.194821	-3.506484	3.967252
H	0.467928	-3.810783	3.60928
H	0.944487	-2.371881	4.557736
H	2.161083	-1.440709	2.509333
H	1.664914	-2.881243	1.58422
H	-3.481565	6.321716	-1.192289
H	-4.515032	5.164823	-1.917267
H	-1.192788	5.742357	-0.968473
H	-0.764517	3.989989	-0.801227
H	-2.240022	2.441109	-1.559442
H	-4.309341.53148	-1.606711	
H	-4.838542.942725	-2.560999	
H	-4.996272	2.969446	-0.768153
H	-7.182147	3.240861	2.492332
H	-7.542991.497883	2.332341	
H	-6.915594	2.430631	0.923321
H	-5.130982	2.466569	3.280838
H	-4.55287-0.292651	0.349128	
H	-6.219220.164499	0.793346	
H	-2.864697	0.299222	1.581027
H	-2.9021 1.738752	2.584071	
H	-4.148045	-5.156132	1.867993
H	-4.551886	-3.430714	1.68666
H	-2.975012	-3.896957	2.39818
H	-1.494704	-3.108661	0.847438
H	-1.618609	-2.055315	-2.988811
H	-3.135196	-2.383509	-2.064292
H	-1.623417	-4.294179	-1.867898
H	2.708919	-3.114204	-0.899285
H	3.693511	-2.20355-3.026236	
H	2.259122	-3.262644	-3.27808
H	6.218767	1.314063	2.268652
H	7.872933	3.197464	1.830755
H	6.211616	3.831826	2.032985
H	6.929758	3.696436	0.399112
H	7.247347	1.329026	-0.635957
H	8.214216	0.860068	0.791642
H	2.81268 1.79973	-0.521738	
H	1.472947	1.250916	2.198942
H	1.759356	2.858259	1.433937
H	5.843618	-0.488875	-0.066829
H	4.832314	2.171911	-0.217103
H	1.066627	-1.209859	-3.370976
H	2.517062	4.162838	-1.320994
H	1.52195 3.051927	-1.696972	

Table S46. Optimized coordinates for five-coordinate NHase, S=5/2.

Fe	0.251577	-0.620690.024286	
S	-0.245831	-1.398252.100028	
S	-0.089875	1.840282	0.312372
S	-1.923659	-0.670718	-1.130432

O	0.012749	2.576995	-1.128446
O	-2.353203	0.3451	-2.183617
O	-2.917456	-0.793773	0.060839
N	0.812928	-2.138891	-1.158156
C	0.2383	-3.383683	-1.272483
O	0.861998	-4.438466	-1.480374
N	2.137882	-0.069657	-0.090478
C	2.95471	-0.878089	-0.808782
O	4.17611	-0.690126	-1.012261
O	2.168438	1.993712	-2.817646
C	-6.783378	2.372601	2.038579
N	-5.393637	2.132808	2.424956
C	-4.639361	1.163923	1.858457
N	-5.179845	0.335612	0.949339
N	-3.338443	1.007105	2.190834
C	-4.271072	2.67227	-1.572155
N	-2.910439	3.176603	-1.490132
C	-2.622322	4.452358	-1.195608
N	-3.644979	5.354737	-1.065079
N	-1.354676	4.815186	-0.953355
N	4.974362	1.870366	0.760303
C	6.316966	1.784811	1.349417
C	6.939843	3.184269	1.500079
C	7.184532	0.83751	0.482816
O	6.670851	-0.481396	0.406292
C	2.655184	1.243696	0.302371
C	1.565451	1.995153	1.088313
C	3.900958	1.154623	1.220755
O	3.873545	0.560864	2.31129
C	2.261163	-2.084058	-1.472778
C	2.449307	-1.96854	-3.004985
O	1.738921	-0.839668	-3.544877
N	-1.849528	-3.683632	0.122063
C	-1.321539	-3.457094	-1.228484
C	-2.044841	-2.305426	-1.966164
C	-3.698598	-4.117579	1.68785
C	-3.115896	-4.201799	0.276933
O	-3.757216	-4.688593	-0.671019
C	1.25617	-3.006082	3.793722
C	1.399108	-2.141687	2.546892
H	2.234924	-3.44723	4.061741
H	0.542327	-3.83245	3.629441
H	0.903097	-2.413204	4.656248
H	2.123606	-1.320931	2.685277
H	1.73853	-2.750688	1.691855
H	-3.386335	6.337832	-0.989883
H	-4.488013	5.193169	-1.616286
H	-1.0979	5.800361	-0.951551
H	-0.617891	4.04035	-1.012058
H	-2.127128	2.518594	-1.640112
H	-4.20229	1.581574	-1.676386
H	-4.807104	3.069243	-2.457504
H	-4.844796	2.92838	-0.663705
H	-7.108354	3.319448	2.493383

H	-7.461464	1.570368	2.386872
H	-6.862722	2.469027	0.942465
H	-5.035622.58565	3.264315	
H	-4.531167	-0.280209	0.412753
H	-6.187447	0.223245	0.871274
H	-2.823892	0.325677	1.602247
H	-2.808506	1.81127 2.52612	
H	-4.193692	-5.071103	1.934019
H	-4.4667 -3.324035		1.702063
H	-2.942967	-3.883856	2.456754
H	-1.450364	-3.122821	0.889539
H	-1.631795	-2.162809	-2.979288
H	-3.123979	-2.529346	-2.035674
H	-1.567488	-4.368827	-1.800332
H	2.761506	-3.01397-1.140227	
H	3.514421	-1.809286	-3.235388
H	2.106601	-2.907594	-3.481683
H	6.1737 1.316714		2.338615
H	7.933017	3.11482 1.97867	
H	6.299453	3.832656	2.12178
H	7.072594	3.672186	0.516
H	7.313232	1.298928	-0.525654
H	8.191716	0.761926	0.93464
H	2.888595	1.797644	-0.631851
H	1.451519	1.55437 2.09729	
H	1.806871	3.067196	1.191559
H	5.79423 -0.445422		-0.055462
H	4.922852	2.188401	-0.210471
H	0.812094	-0.950922	-3.233079
H	2.112751	1.030065	-3.02907
H	1.357435	2.160643	-2.264161

Table S47. Optimized coordinates for acetonitrile-coordinated NHase, backbone amidates substituted for histidines, S=1/2.

Fe	1.104545	0.029371	-0.136387
S	1.025848	-1.095646	1.736231
S	-0.637361	1.423657	0.725996
S	-0.633086	-1.200656	-0.928941
O	-0.998415	2.607611	-0.3036
O	-1.352809	-0.302684	-1.96286
O	-1.598497	-1.789647	0.104742
N	2.422001	-1.184174	-1.080726
N	2.627789	1.232419	0.343725
O	0.953856	1.051723	-1.85885
O	1.019495	3.618797	-1.553784
C	-6.841996	-1.811761	1.742632
N	-5.573907	-1.331366	2.269412
C	-4.382444	-1.612781.711496	
N	-4.271923	-2.523075	0.723496
N	-3.27241-0.967102		2.135222
C	-4.70671-0.236613		-1.778485
N	-3.867945	0.930532	-1.572002
C	-4.362243	2.169804	-1.435058

N	-5.694781	2.387094	-1.625855
N	-3.559782	3.163316	-1.021921
C	-0.093403	2.356455	2.202753
C	-0.169006	-2.67639-1.898398	
C	2.698157	-2.560894	3.400512
C	2.598763	-1.188571	2.721653
C	3.507115	1.678918	-0.565562
N	4.444461	2.464597	0.029444
C	4.179402	2.537062	1.391302
C	3.044618	1.765721	1.562252
C	5.021759	3.306824	2.359932
C	2.828226	-1.021131	-2.348381
N	3.721463	-1.993935	-2.677199
C	3.911479	-2.82876-1.583465	
C	3.090509	-2.305057	-0.600439
C	4.83187 -4.008819		-1.593299
H	3.603198	-2.600097	4.033909
H	2.758444	-3.380343	2.662766
H	1.821809	-2.751547	4.043929
H	2.579071	-0.394974	3.48882
H	3.462474	-0.998378	2.062448
H	-6.042819	3.34192 -1.552567	
H	-6.195677	1.801209	-2.293057
H	-3.858568	4.132244	-1.114369
H	-2.569553	2.957531	-0.713887
H	-2.845303	0.766641	-1.582569
H	-4.094834	-1.126749	-1.585054
H	-5.088644	-0.301993	-2.816727
H	-5.562661	-0.233692	-1.081879
H	-7.646414	-1.170363	2.131287
H	-7.056155	-2.854332	2.044786
H	-6.846296	-1.745519	0.641636
H	-5.577029	-0.7788 3.125554	
H	-3.341178	-2.631924	0.292711
H	-4.946207	-3.280986	0.63685
H	-2.410404	-1.110603	1.574253
H	-3.380691	-0.048269	2.563488
H	-1.118858	-3.126753	-2.227537
H	0.389559	-3.365426	-1.24711
H	-0.961944	2.949334	2.532112
H	0.748818	3.023502	1.960703
H	1.708367	3.880536	-0.907455
H	0.191516	3.424852	-0.981355
H	0.036864	0.889161	-2.216903
H	1.096079	2.079104	-1.79279
H	0.188857	1.63784 2.988967	
H	0.43895 -2.369985		-2.761883
H	3.500617	1.457603	-1.628566
H	5.222258	2.914346	-0.454005
H	4.596227	3.225589	3.372608
H	5.071836	4.37929 2.097645	
H	6.057038	2.921567	2.395642
H	2.522974	1.555454	2.490539
H	2.474595	-0.248175	-3.02653

H	4.172164	-2.090214	-3.587529
H	2.923464	-2.679133	0.406448
H	4.78974 -4.52311-0.620398		
H	5.880435	-3.708333	-1.772752
H	4.552929	-4.737916	-2.375709

Table S48. Optimized coordinates for acetonitrile-coordinated NHase, backbone amidates substituted for histidines, S=3/2.

Fe	1.16782 0.061039	-0.119074	
S	1.065496	-1.097734	1.744686
S	-0.813756	1.604893	0.707511
S	-0.67296-1.255658	-0.912226	
O	-1.076775	2.869608	-0.278542
O	-1.354556	-0.363329	-1.971972
O	-1.647687	-1.835413	0.114842
N	2.434088	-1.24352-1.236123	
N	2.681026	1.254987	0.354732
O	0.96281 1.063091	-1.858121	
O	0.920673	3.615573	-1.685133
C	-6.846831	-1.835086	1.738537
N	-5.586272	-1.333489	2.265162
C	-4.387758	-1.628151	1.728411
N	-4.269467	-2.581502	0.783993
N	-3.286743	-0.956639	2.137127
C	-4.682764	-0.301098	-1.783142
N	-3.845858	0.873896	-1.597661
C	-4.349406	2.111026	-1.469449
N	-5.699409	2.288204	-1.613161
N	-3.567392	3.145169	-1.139148
C	-0.116812.362083	2.206347	
C	-0.136085	-2.72663-1.825707	
C	2.679226	-2.528745	3.498488
C	2.607756	-1.173328	2.781383
C	3.539516	1.733888	-0.559789
N	4.459575	2.524821	0.04453
C	4.194521	2.567286	1.410156
C	3.086359	1.768874	1.579068
C	4.993468	3.332082	2.399842
C	2.912848	-1.109636	-2.476864
N	3.838369	-2.079074	-2.722804
C	3.960753	-2.873362	-1.588697
C	3.076883	-2.335364	-0.681641
C	4.865963	-4.037642	-1.454146
H	3.571463	-2.556012	4.150299
H	2.747988	-3.365794	2.782181
H	1.788982	-2.694249	4.129509
H	2.575165	-0.359097	3.525187
H	3.490802	-1.013498	2.139592
H	-6.057185	3.242005	-1.576106
H	-6.1984 1.684231	-2.266091	
H	-3.953701	4.087015	-1.163881
H	-2.567623.017611	-0.782458	
H	-2.824929	0.715665	-1.640891

H	-4.061004	-1.186678	-1.599318
H	-5.086371	-0.373018	-2.812732
H	-5.523337	-0.29994-1.068316	
H	-7.66011-1.200582	2.11933	
H	-7.048691	-2.877082.050646	
H	-6.849166	-1.780966	0.63671
H	-5.601647	-0.740865	3.093908
H	-3.343755	-2.696396	0.345486
H	-4.943132	-3.342748	0.72617
H	-2.425308	-1.120061	1.583234
H	-3.407559	-0.009073	2.495403
H	-1.057763	-3.233189	-2.156498
H	0.439232	-3.369191	-1.142672
H	-0.871553.031018	2.65214	
H	0.791566	2.93587	1.965004
H	1.621584	3.979851	-1.105483
H	0.106081	3.469556	-1.061545
H	0.072464	0.840241	-2.242879
H	1.054498	2.107566	-1.836758
H	0.118511	1.548902	2.913714
H	0.474204	-2.42107-2.68676	
H	3.525561	1.51851	-1.624631
H	5.229144	2.998286	-0.430755
H	4.55904	3.209478	3.404458
H	5.012503	4.413094	2.169328
H	6.042057	2.984205	2.441204
H	2.567408	1.538283	2.503559
H	2.604623	-0.353018	-3.195603
H	4.355967	-2.198042	-3.594555
H	2.852401	-2.682049	0.32482
H	4.785181	-4.454705	-0.437767
H	5.924601	-3.764336	-1.621762
H	4.619456	-4.844139	-2.169908

Table S49. Optimized coordinates for acetonitrile-coordinated NHase, backbone amidates substituted for histidines, S=5/2.

Fe	1.262517	0.060839	-0.091362
S	0.95881	-1.191767	1.835903
S	-0.756979	1.551783	0.795392
S	-0.76093-1.21699-1.046343		
O	-1.007562	2.873177	-0.100362
O	-1.402878	-0.331856	-2.144529
O	-1.784079	-1.79535-0.058226	
N	2.459585	-1.24168-1.207255	
N	2.731492	1.437031	0.239416
O	0.92701	1.092759	-1.955777
O	0.802371	3.685044	-1.746002
C	-6.873184	-1.829446	1.665962
N	-5.605115	-1.378109	2.222747
C	-4.410569	-1.669671.672077	
N	-4.316949	-2.560641	0.669209
N	-3.289556	-1.064012.12983	
C	-4.667593	-0.174361	-1.774213

N	-3.807349	0.978231	-1.553082
C	-4.289414	2.213577	-1.347035
N	-5.640927	2.410793	-1.431778
N	-3.480322	3.225011	-1.008916
C	-0.065809	2.210779	2.347797
C	-0.168028	-2.682498	-1.918259
C	2.631536	-2.788141	3.416863
C	2.539911	-1.391517	2.797082
C	3.548179	2.097782	-0.590505
N	4.481217	2.771579	0.13167
C	4.2623 2.530671	1.48319	
C	3.171614	1.698428	1.524868
C	5.061364	3.07198 2.600108	
C	2.946156	-1.056121	-2.440542
N	3.835275	-2.040951	-2.735609
C	3.93284 -2.903893	-1.646826	
C	3.067434	-2.384785	-0.70888
C	4.806668	-4.105606	-1.595173
H	3.556353	-2.867434	4.017845
H	2.65508 -3.576571	2.644479	
H	1.771547	-2.986534	4.078738
H	2.530381	-0.626275	3.593667
H	3.405978	-1.187624	2.144223
H	-5.986788	3.365808	-1.345734
H	-6.169031.836282	-2.088706	
H	-3.865121	4.165422	-0.940869
H	-2.492121	3.060183	-0.645119
H	-2.792650.80654 -1.667679		
H	-4.043751	-1.07294-1.688785	
H	-5.128366	-0.168406	-2.782189
H	-5.467516	-0.214756	-1.015743
H	-7.675862	-1.194997	2.069419
H	-7.099412	-2.880044	1.929404
H	-6.865993	-1.724158	0.568497
H	-5.611299	-0.865433.103129	
H	-3.409607	-2.636691	0.181431
H	-5.032424	-3.269540.52519	
H	-2.442053	-1.205321	1.549749
H	-3.383582	-0.138032.547839	
H	-1.053074	-3.201886	-2.323243
H	0.358211	-3.323736	-1.195237
H	-0.813611	2.887309	2.793695
H	0.868212	2.763098	2.159283
H	1.562982	4.069253	-1.263733
H	0.099067	3.505345	-1.020915
H	0.06202 0.813803	-2.360311	
H	0.953617	2.123674	-1.939787
H	0.116476	1.360963	3.025589
H	0.504919	-2.376449	-2.731978
H	3.501411	2.093153	-1.676859
H	5.228605	3.346806	-0.259476
H	4.655893	2.708029	3.557227
H	5.046753	4.177415	2.627092
H	6.121122	2.760773	2.54356

H	2.69201 1.271931		2.401056
H	2.662561	-0.247353	-3.110972
H	4.342861	-2.13091-3.616889	
H	2.830684	-2.774003	0.279496
H	4.668999	-4.62225-0.632504	
H	5.876946	-3.844323	-1.690272
H	4.568944	-4.82441-2.40097	

Table S50. Optimized coordinates for acetonitrile-coordinated NHase, backbone amidates substituted for acetates, S=1/2.

Fe	-1.457493	-0.138283	0.173685
S	-1.423411	-1.801006	-1.347901
S	0.099805	0.847534	-1.327655
S	0.188601	-1.065333	1.272029
O	0.782393	2.194051	-0.715052
O	1.007591	0.032913	1.981875
O	1.117105	-1.997802	0.451
O	-2.82015-1.007821		1.261804
C	-3.274403	-0.833633	2.475593
O	-2.694865	-0.295105	3.433
O	-3.061947	0.661329	-0.707498
C	-3.700373	1.749864	-0.941189
O	-3.266484	2.93618 -0.927169	
O	-1.267782	1.392731	1.393923
O	-1.094776	3.726336	0.383579
C	6.406518	-2.298784	-0.701298
N	5.176123	-2.001261	-1.411448
C	3.953092	-2.071273	-0.844032
N	3.79066 -2.552831		0.406275
N	2.876489	-1.635396	-1.526338
C	4.235925	0.726217	1.660464
N	3.438678	1.665628	0.891023
C	3.965757	2.72356 0.258369	
N	5.248157	3.105374	0.519045
N	3.242782	3.316621	-0.711344
C	-0.918657	1.519657	-2.711247
C	-0.312956	-2.189349	2.615703
C	-3.075251	-3.132465	-3.160732
C	-3.02469-1.872413		-2.276801
C	-5.182385	1.543854	-1.286074
C	-4.69515-1.399431		2.642462
H	-4.037555	-3.176481	-3.704901
H	-2.980862	-4.050038	-2.553443
H	-2.259455	-3.133142	-3.906031
H	-3.140107	-0.960653	-2.889291
H	-3.838331	-1.870577	-1.533346
H	5.594566	3.948855	0.064086
H	5.615939	2.942167	1.455892
H	3.428816	4.28902 -0.95333	
H	2.276245	2.916955	-0.895409
H	2.432397	1.470865	0.755921
H	3.599365	-0.140921.880417	
H	4.569028	1.155174	2.625963

H	5.124041	0.402649	1.08895
H	7.249188	-1.867647	-1.261712
H	6.58437	-3.386419	-0.596701
H	6.386506	-1.841540.302771	
H	5.223566	-1.787231	-2.405513
H	2.807559	-2.541577	0.753557
H	4.435285	-3.255453	0.764752
H	1.968347	-1.628078	-1.005567
H	2.998738	-0.960495	-2.280002
H	0.623652	-2.544261	3.075719
H	-0.879672	-3.022689	2.172709
H	-0.222599	1.932906	-3.460321
H	-1.595966	2.296027	-2.324569
H	-1.900977	3.541336	-0.19831
H	-0.328826	3.314633	-0.137113
H	-0.425948	1.274513	1.903931
H	-1.2832	2.385936	1.053934
H	-1.497136	0.689636	-3.144544
H	-0.936626	-1.619528	3.321601
H	-5.559372	2.378777	-1.89869
H	-4.945987	-1.500273.710632	
H	-4.793573	-2.373989	2.134956
H	-5.416829	-0.708347	2.170043
H	-5.763097	1.518535	-0.345433
H	-5.340052	0.583328	-1.802492

Table S51. Optimized coordinates for acetonitrile-coordinated NHase, backbone amidates substituted for acetates, S=3/2.

Fe	-1.576172	-0.154776	0.156255
S	-1.486462	-1.683428	-1.471495
S	0.281811	1.126575	-1.345561
S	0.253243	-1.236267	1.165662
O	0.815594	2.51782	-0.648587
O	1.106651	-0.166929	1.874523
O	1.113778	-2.190982	0.302033
O	-2.708797	-1.229593	1.356407
C	-3.269144	-1.090704	2.528883
O	-2.715295	-0.698496	3.569133
O	-3.056124	0.795844	-0.666852
C	-3.719396	1.889515	-0.832729
O	-3.281105	3.065819	-0.777153
O	-1.295172	1.266001	1.50886
O	-1.121676	3.645834	0.701188
C	6.392168	-2.274563	-0.822563
N	5.174455	-1.834798	-1.481228
C	3.940659	-2.025439	-0.962349
N	3.772884	-2.676240.204824	
N	2.864501	-1.543612	-1.616929
C	4.224362	0.604483	1.717466
N	3.430876	1.600806	1.017467
C	3.971365	2.688881	0.444834
N	5.274624	3.004622	0.721355
N	3.27068	3.377427	-0.466121

C	-0.903506	1.703261	-2.619464
C	-0.343794	-2.342207	2.461974
C	-3.080745	-2.899114	-3.376338
C	-3.071564	-1.696372	-2.422273
C	-5.174381.658688	-1.216372	
C	-4.722103	-1.532522	2.517979
H	-4.024994	-2.923993	-3.952709
H	-2.992867	-3.848519	-2.819338
H	-2.243185	-2.848391	-4.09517
H	-3.184019	-0.748439	-2.977265
H	-3.903402	-1.759555	-1.701306
H	5.627362	3.8859	0.349859
H	5.631791	2.769766	1.647619
H	3.552962	4.330979	-0.689021
H	2.26117	3.046563	-0.688845
H	2.423773	1.423822	0.894453
H	3.57335	-0.261091.899639	
H	4.582881	0.971675	2.699569
H	5.096133	0.29911	1.111848
H	7.247614	-1.765788	-1.291065
H	6.551249	-3.367239	-0.9055
H	6.367469	-1.994101	0.244063
H	5.229453	-1.538148	-2.454007
H	2.790999	-2.722703	0.550898
H	4.451461	-3.372035	0.507995
H	1.953101	-1.638111	-1.114582
H	2.973147	-0.740488	-2.236166
H	0.553486	-2.781393	2.93215
H	-0.957859	-3.116391.979522	
H	-0.355355	2.209861	-3.432512
H	-1.627541	2.389127	-2.154707
H	-1.910911	3.55874	0.080427
H	-0.331333.343001	0.124334	
H	-0.452072	1.092439	1.993478
H	-1.290275	2.300022	1.241835
H	-1.418742	0.813714	-3.012807
H	-0.945568	-1.754526	3.173692
H	-5.736123	2.605757	-1.224516
H	-5.170761	-1.466633.522856	
H	-4.795716	-2.571075	2.149422
H	-5.300595	-0.902145	1.819124
H	-5.645908	0.94735	-0.517444
H	-5.219684	1.203633	-2.222437

Table S52. Optimized coordinates for acetonitrile-coordinated NHase, backbone amidates substituted for acetates, S=5/2.

Fe	-1.651286	-0.111804	0.112075
S	-1.326997	-1.799843	-1.531773
S	0.220247	1.074483	-1.362262
S	0.367607	-1.212072	1.255505
O	0.718061	2.498902	-0.720931
O	1.20605	-0.158742	2.019413
O	1.251522	-2.176730.41794	

O	-2.731667	-1.139481.336688	
C	-3.285731	-1.061388	2.525585
O	-2.741322	-0.641674	3.553836
O	-3.016742	0.99074	-0.606356
C	-3.851339	1.955796	-0.849189
O	-3.580954	3.170398	-0.765381
O	-1.118113	1.320768	1.54561
O	-1.215431	3.712456	0.598691
C	6.437384	-2.187025	-0.778106
N	5.211713	-1.788639	-1.454625
C	3.981019	-1.998651	-0.927067
N	3.849838	-2.610498	0.262967
N	2.887032	-1.5821	-1.596209
C	4.198875	0.704796	1.684902
N	3.384629	1.670516	0.968549
C	3.899761	2.749535	0.359041
N	5.200978	3.093803	0.60641
N	3.173123	3.402494	-0.558727
C	-0.945498	1.572838	-2.684663
C	-0.300934	-2.327342.499269	
C	-3.013969	-3.096775	-3.326056
C	-3.028478	-2.038528	-2.21748
C	-5.216651.464815	-1.285861	
C	-4.696949	-1.618529	2.529446
H	-4.033616	-3.249991	-3.729746
H	-2.646973	-4.065032	-2.942525
H	-2.35638-2.790978	-4.159171	
H	-3.402293	-1.072547	-2.602811
H	-3.694914	-2.344233	-1.391972
H	5.535031	3.969717	0.206208
H	5.577768	2.8851	1.531032
H	3.433903	4.354581	-0.811539
H	2.170712	3.048894	-0.763646
H	2.380144	1.47363	0.866196
H	3.551461	-0.151447	1.917698
H	4.580934	1.111213	2.642442
H	5.056003	0.378602	1.068871
H	7.287608	-1.708517	-1.28621
H	6.59794	-3.282715	-0.794197
H	6.421859	-1.839075	0.268666
H	5.260767	-1.553233	-2.444382
H	2.876224	-2.655028	0.642255
H	4.571103	-3.244032	0.6005
H	1.988418	-1.679084	-1.070036
H	2.96865	-0.799489	-2.245015
H	0.552818	-2.806534	3.010166
H	-0.911989	-3.075694	1.972776
H	-0.384566	2.03764	-3.513836
H	-1.675461	2.285952	-2.274635
H	-2.051211	3.589579	0.058953
H	-0.468893.391074	-0.013803	
H	-0.237313	1.15207	1.96213
H	-1.186863	2.323199	1.270746
H	-1.453193	0.664125	-3.040801

H	-0.921404	-1.741623.195733	
H	-5.906474	2.303887	-1.468179
H	-5.156696	-1.531634	3.527127
H	-4.676236	-2.681352	2.228645
H	-5.320108	-1.086793	1.789209
H	-5.637562	0.801621	-0.510662
H	-5.116835	0.864535	-2.207212

Table S53. Optimized coordinates for acetonitrile.

C	0.000000	0.000000	0.280082
C	0.000000	0.000000	-1.176055
H	0.000000	1.028442	-1.550789
H	0.890657	-0.514221	-1.550789
H	-0.890657	-0.514221	-1.550789
N	0.000000	0.000000	1.432601

Table S54. Optimized coordinates for acetamide.

N	-0.8306	1.1720	-0.0035
C	-0.0896	-0.0482	0.0033
O	-0.6828	-1.1317	0.0098
C	1.4105	-0.0108	0.0019
H	-0.3344	2.0737	-0.0083
H	1.7677	-0.9448	0.0072
H	1.7331	0.4735	0.8151
H	1.7318	0.4637	-0.8177
H	-1.8590	1.1494	-0.0022

Table S55. Optimized coordinates for water.

O	0.000000	0.000000	0.122103
H	0.000000	0.764275	-0.488412
H	0.000000	-0.764275	-0.488412

Supporting References

1. Nojiri, M.; Yohda, M.; Odaka, M.; Matsushita, Y.; Tsujimura, M.; Yoshida, T.; Dohmae, N.; Takio, K.; Endo, I. *J. Biochem.* **1999,** *125,* 696-704.
2. Nilges, M. J., Mattson, K., and Belford, R. L. *Spectrosc. Membr. Biophys.* **2007,** *27,* 261-281. 27
3. Gaussian 09, Revision **C.01**, Frisch, M. J.; Trucks, G. W.; Schlegel, H. B.; Scuseria, G. E.; Robb, M. A.; Cheeseman, J. R.; Scalmani, G.; Barone, V.; Mennucci, B.; Petersson, G. A.; Nakatsuji, H.; Caricato, M.; Li, X.; Hratchian, H. P.; Izmaylov, A. F.; Bloino, J.; Zheng, G.; Sonnenberg, J. L.; Hada, M.; Ehara, M.; Toyota, K.; Fukuda, R.; Hasegawa, J.; Ishida, M.; Nakajima, T.;

Honda, Y.; Kitao, O.; Nakai, H.; Vreven, T.; Montgomery, J. A., Jr.; Peralta, J. E.; Ogliaro, F.; Bearpark, M.; Heyd, J. J.; Brothers, E.; Kudin, K. N.; Staroverov, V. N.; Kobayashi, R.; Normand, J.; Raghavachari, K.; Rendell, A.; Burant, J. C.; Iyengar, S. S.; Tomasi, J.; Cossi, M.; Rega, N.; Millam, M. J.; Klene, M.; Knox, J. E.; Cross, J. B.; Bakken, V.; Adamo, C.; Jaramillo, J.; Gomperts, R.; Stratmann, R. E.; Yazyev, O.; Austin, A. J.; Cammi, R.; Pomelli, C.; Ochterski, J. W.; Martin, R. L.; Morokuma, K.; Zakrzewski, V. G.; Voth, G. A.; Salvador, P.; Dannenberg, J. J.; Dapprich, S.; Daniels, A. D.; Farkas, Ö.; Foresman, J. B.; Ortiz, J. V.; Cioslowski, J.; Fox, D. J. Gaussian, Inc., Wallingford CT, 2009.

4. Grimme, S.; Antony, J.; Ehrlich, S.; Krieg, H. *J. Chem. Phys.* **2010**, *132*, 154104.
5. Neese, F. *Orca version 2.9.1*; Max-Plank Institute für Chemishe Energie Konversion: Mülheim/Ruhr, Germany.
6. Tomasi, J.; Mennucci, B.; Cammi, R. *Chem. Rev.* **2005**, *105*, 2999–3093.
7. *LUMO version 1.0.1;* Matthew T. Kieber-Emmons: Ephrata, PA, 2012.
8. Tenderholt, Adam L. *QMForge*, Version 2.3.2, http://qmforge.sourceforge.net.
